# 1,2-Oxidative Trifluoromethylation of Olefin with Ag(O_2_CCF_2_SO_2_F) and O_2_: Synthesis of α-Trifluoromethyl Ketones

**DOI:** 10.3390/molecules29235622

**Published:** 2024-11-27

**Authors:** Shengxue Zhang, Wangchuan Xiao, Jingjing Wu, Fanhong Wu, Houjin Huang, Xiaoyu Ma, Yafei Shi, Chao Liu

**Affiliations:** 1 School of Chemical and Environmental Engineering, Shanghai Institute of Technology, 100 Haiquan Road, Shanghai 201418, China; 2 Shanghai-Sanming Engineering Research Center of Green Fluoropharmaceutical Technology, 25 Jingdong Road, Sanming 365004, China; 3 Key Laboratory of Fluorine and Nitrogen Chemistry and Advanced Materials, Shanghai Institute of Organic Chemistry, University of Chinese Academy of Sciences, Chinese Academy of Sciences, 345 Lingling Road, Shanghai 200032, China

**Keywords:** Ag(O_2_CCF_2_SO_2_F), trifluoromethylation, α-trifluoromethyl ketones

## Abstract

A novel and efficient 1,2-oxidative trifluoromethylation of olefins employing Ag(O_2_CCF_2_SO_2_F) as a trifluoromethyl source is described with O_2_ as the oxidant, which provides access to a variety of valuable α-trifluoromethyl-substituted ketones. The broad substrate scope, feasibility of large-scale operation, and derivatization reactions of α-trifluoromethyl ketones demonstrate the promising utility of this protocol.

## 1. Introduction

α-Trifluoromethyl ketones are an important class of organofluorine compounds with good lipophilicity, metabolic stability, and strong electron-withdrawing ability. These distinctive properties have facilitated their widespread application in the realm of drug design and synthesis [1,2]. Furthermore, due to their multifunctional nature and the unique reactivity of their carbonyl groups, α-trifluoromethyl ketones might serve as valuable building blocks for synthesizing various CF_3_-containing complex moieties as well as carbonyl-containing compounds [3,4,5,6]. Conventional approaches for the preparation of α-trifluoromethylated ketones involve the radical or electrophilic trifluoromethylation of enol acetates, metal enolates, or silyl enol ethers [7,8,9,10,11,12,13] derived from the corresponding aldehydes, ketones, esters, and amides. Recently, several methods for the preparation of α-trifluoromethyl ketones have been developed, including the nucleophilic trifluoromethylation of α-haloketones with fluoroform-derived CuCF_3_ reagent [14], radical desulfurization cleavage and reconstruction of enol triflates [15], and fluoroaroylation of *gem*-difluoroalkenes with aryl fluorides [16]. However, these methods necessitate the utilization of preformed substrates as raw materials, which leads to inefficiencies and elevated costs.

**Scheme 1:**
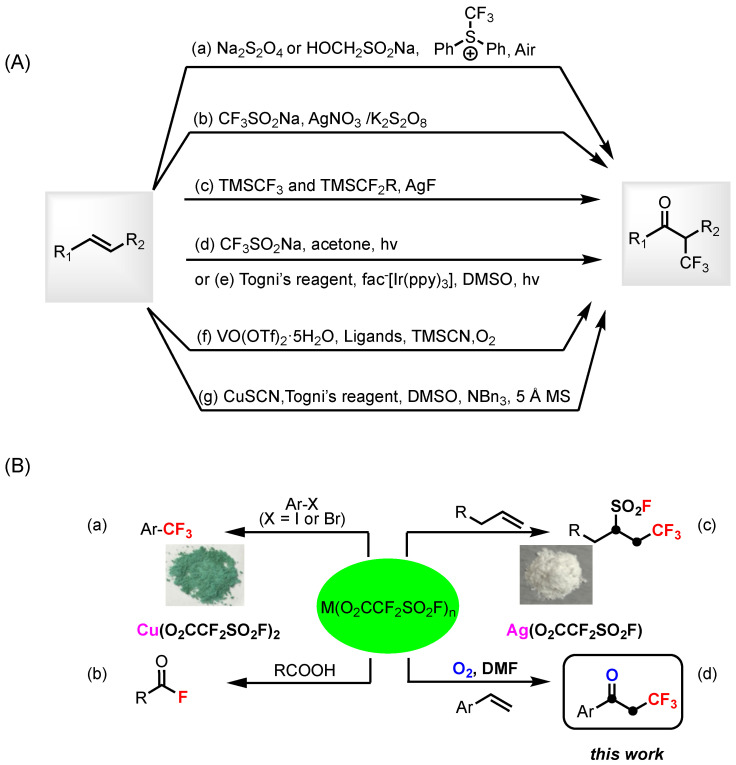
(**A**) Synthetic approaches of α-trifluoromethyl-substituted ketones; (**B**) Application of Chen’s reagent derivatives.

Conversely, olefins are cost-effective, plentiful, and readily available raw materials. The direct 1,2-oxidative trifluoromethylation of olefins represents a more immediate and promising approach to synthesizing α-trifluoromethylated ketones. In 2011, Xiao and co-workers [17] achieved the direct 1,2-oxidative cross-couplings of olefins with in situ generated CF_3_ radical by mixing S-(trifluoromethyl)diphenylsulfonium triflate with Na_2_S_2_O_4_ or HOCH_2_SO_2_Na. However, the yields were only 20–40% (Scheme 1(Aa)). Subsequently, Maiti’s group [18] disclosed the oxidative trifluoromethylation of unactivated olefins with an inexpensive Langlois reagent (CF_3_SO_2_Na) as the CF_3_ source in the presence of a catalytic amount of AgNO_3_/K_2_S_2_O_8_ to access α-CF_3_ -substituted ketones in excellent yields (Scheme 1(Ab)). Cai and co-workers [19] conducted the radical addition of olefins with TMSCF_3_ and TMSCF_2_R (R = COOEt or CF_3_) to deliver various α-trifluoromethylated ketones and α-fluoroolefinated ketones (Scheme 1(Ac)). Moreover, the visible-light-induced oxidative trifluoromethylation of alkenes and alkynes by using CF_3_SO_2_Na or the Togni reagent as the CF_3_ reagent was reported by Akita [20] and Wang [21] (Scheme 1(Ad,Ae)). In 2020, Mori’s group [22] used vanadyl species to catalyze the 1,2-oxidative trifluoromethylation of unactivated olefins with the Togni’s reagent as the CF_3_ radical source under an oxygen atmosphere (Scheme 1(Af)). Most recently, the group of Qi [23] presented the copper-catalyzed oxidative trifluoromethylation of aromatic alkenes, which avoided the use of precious metals (Scheme 1(Ag)). Despite these invaluable advances, the development of general and efficient methods for α-trifluoromethy ketones synthesis is still in high demand.

Our group focused on pioneering novel applications and reactions for Chen’s reagent (FSO_2_CF_2_COOMe). We demonstrated that replacing the methyl ester group in Chen’s reagent with various metal ions results in derivatives exhibiting significantly enhanced activity and a broader reactivity, such as copper salt Cu(O_2_CCF_2_SO_2_F)_2_ and silver compound Ag(O_2_CCF_2_SO_2_F) (Scheme 1B). During our investigations, it was found that Cu(O_2_CCF_2_SO_2_F)_2_ can produce CuCF_3_ species under mild conditions and then rapidly undergo trifluoromethylation reactions with aryl iodide or benzyl bromide substrates (Scheme 1(Ba)) [24,25]. And it can be utilized as a novel dehydroxyfluorination agent, enabling the swift conversion of diverse carboxylic acid compounds into significant acyl fluorinated derivatives (Scheme 1(Bb)) [26]. Ag(O_2_CCF_2_SO_2_F), similarly to Cu(O_2_CCF_2_SO_2_F)_2_, can decompose to produce trifluoromethyl species (AgCF_3_), which show different reactivity compared to CuCF_3_. Although AgCF_3_ complexes have been synthesized for a long time, their reactivity has not been intensely studied. Our findings indicate that Ag(O_2_CCF_2_SO_2_F) serves as an effective CF_3_ radical source, enabling the direct olefinic intermolecular radical trifluoromethylfluorosulfonylation reaction (Scheme 1(Bc)). Notably, the in situ generated SO_2_ from Ag(O_2_CCF_2_SO_2_F) decomposition is efficiently utilized, underscoring the potential of this approach [27]. Building upon our ongoing research into the applications of Chen’s reagent and its derivatives, we sought to investigate the potential for 1,2-oxidative trifluoromethylation of olefins using our Ag(O_2_CCF_2_SO_2_F) as a CF_3_ species in conjunction with an oxidant. This approach offers a means of accessing a range of structurally diverse and valuable α-trifluoromethyl-substituted ketones (Scheme 1(Bd)).

## 2. Results and Discussion

We initiated our attempts at 1,2-oxidative trifluoromethylation with Ag(O_2_CCF_2_SO_2_F) and biphenyl ethylene (**1a**) as the model substrates, and extensive optimization of reaction conditions, including temperature, solvents, etc., was conducted (for details, please see the SI). Ultimately, under optimized conditions, the target product **3a** can be obtained in 87% yield at room temperature for 7 h by the employment of DMF as the reaction solvent, O_2_ as the oxidant, and 4.0 equiv of Ag(O_2_CCF_2_SO_2_F) as the trifluoromethyl reagent (Table 1, entry 1). Replacement of DMF with DMA_C_, NMP, or MeCN resulted in a lower yield of the desired product (entry 2). The decomposition of Ag(O_2_CCF_2_SO_2_F) in these solvents, such as EtOAc, THF, or DCE, is challenging, resulting in the generation of no target products (entry 3). In an effort to enhance the yield of the target compounds, Cu(O_2_CCF_2_SO_2_F)_2_ was employed as a trifluoromethylation reagent in place of Ag(O_2_CCF_2_SO_2_F). However, despite these modifications, the target product remained elusive (entry 4). Furthermore, an attempt to enhance the yield of the desired product by employing the TMSCF_3_/AgF system for the on-site generation of AgCF_3_ as a trifluoromethylation reagent proved inefficient (entry 5). The impact of varying the loading of Ag(O_2_CCF_2_SO_2_F) was also assessed (entries 6–8). The findings indicate that reducing the equivalent of Ag(O_2_CCF_2_SO_2_F) results in a decline in yield, whereas increasing the equivalent does not yield a notable alteration in yield. In consideration of our prior research, Ag(O_2_CCF_2_SO_2_F) decomposes explosively in DMF. Consequently, it is essential to introduce the reactants at low temperatures to regulate their rapid decomposition. An endeavor to enhance the yield of the desired product by modifying the reaction temperature was unsuccessful (entries 9–12). Furthermore, the role of the oxidant in the reaction is noteworthy, as the absence of O_2_ or other oxidants led to a notable reduction in the yield of **3a** (entries 13–15).

**Scheme 2:**
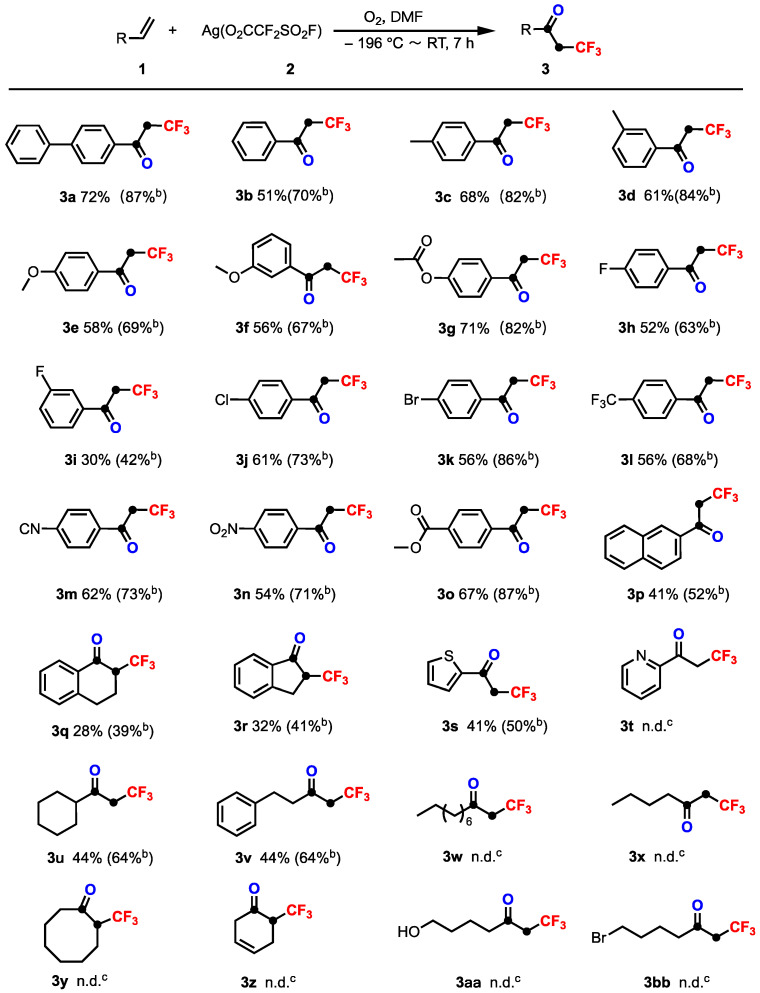
Reaction scope. General reaction conditions: **1** (0.2 mmol, 1.0 equiv), Ag(O_2_CCF_2_SO_2_F) (0.8 mmol, 4.0 equiv), DMF (4 mL), O_2_ atmosphere, room temperature, 7 h. Yields of isolated products were reported. ^b^ Yields were determined by ^19^F NMR spectroscopy using (0.2 mmol, 1.0 equiv) 1-methoxy-4-(trifluoromethoxy)benzene as an internal standard. ^c^ n.d. = not detected.

Having successfully established the optimal reaction conditions, we proceeded to explore the substrate scope of olefin. As illustrated in Scheme 2, the present methodology enables the synthesis of a diverse range of α-trifluoromethyl ketones from both electron-rich and electron-deficient olefins. Initially, the tolerance of olefin functional groups was examined, and the results demonstrated that styrene-bearing aryl, methyl, methoxy, fluorine, chlorine, and bromine functional groups afforded the desired products **3a**–**3k** in modest to good yields (30–72%). The yield was found to be similar when styrene was substituted at the para position (**3c** and **3d**, **3e** and **3f**, and **3h** and **3i**) in comparison to the substituents in the meta position, indicating that the steric and electronic effects observed for meta- and para-substituents were minimal. It is noteworthy that the presence of AcO groups on styrene facilitates the separation of the product (**3g**). Moreover, the reaction was compatible with valuable and sensitive functional groups, including trifluoromethyl (**3l**), cyano (**3m**), nitro (**3n**), and ester groups (**3o**), under the current conditions, providing the desired products in 54–67% isolated yields. It was gratifying to observe that 2-naphthyl (**1p**), cyclic olefins (**1q** and **1r**) with varying ring sizes (5- or 6-membered), and heterocyclic olefins (**1s**) exhibited favorable reactivity. However, vinyl pyridine (**1t**) is unsuitable for this condition probably due to its alkaline properties. Furthermore, other olefins were employed, including vinylcyclohexane (**1u**), 4-phenyl-1-butene (**1v**), 1-decene (**1w**), 1-hexene (**1x**), cyclooctene (**1y**), cyclohexa-1,4-diene (**1z**), 5-hexen-1-ol (**1aa**) and 6-bromo-1-hexene (**1bb**). The aforementioned olefins were employed as raw materials under standard conditions. It was observed that only vinylcyclohexane and 4-phenyl-1-butene yielded the corresponding α-trifluoromethyl ketones in satisfactory yields. It is conceivable that the reactivity of aliphatic olefins is less pronounced, which restricts the applicability of this condition.

**Scheme 3:**
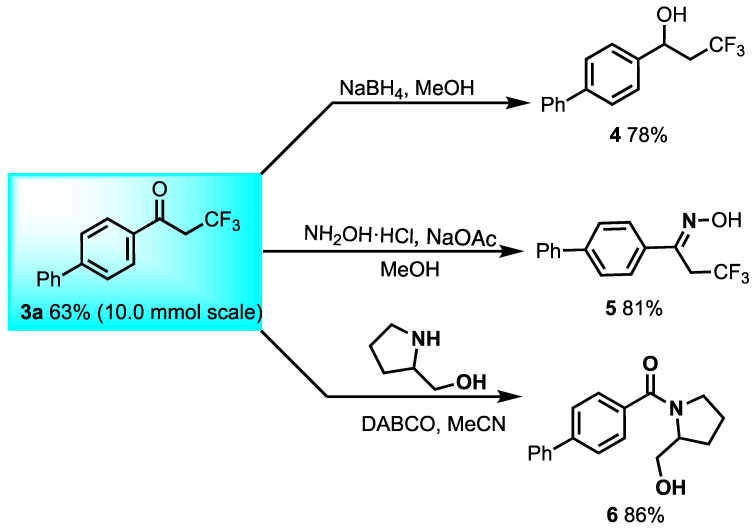
Scale-up and derivatization reactions of the α-trifluoromethyl ketones.

To further examine the potential synthetic utility of this protocol, gram-scale synthesis of α-trifluoromethyl ketones **3a** and its associated derivatization reactions were conducted. As illustrated in Scheme 3, the target product **3a** was isolated in 61% yield on a 10.0 mmol scale under standard reaction conditions. Subsequently, the valuable transformations of **3a** into different valuable structures were conducted. Initially, the reaction of **3a** with NaBH_4_ in MeOH at room temperature for 2 h yielded α-trifluoromethyl alcohol **4** in 78% isolated yield [28]. Secondly, **3a** can react with hydroxylamine hydrochloride to generate the corresponding α-trifluoromethyl oxime (**5**) [29], which can be further converted into ester compounds. Furthermore, an effort was made to preserve the carbonyl group and convert the trifluoromethyl group into alternative functional groups. The synthesis resulted in the isolation of amide (**6**) compounds in high yields [4].

**Scheme 4:**
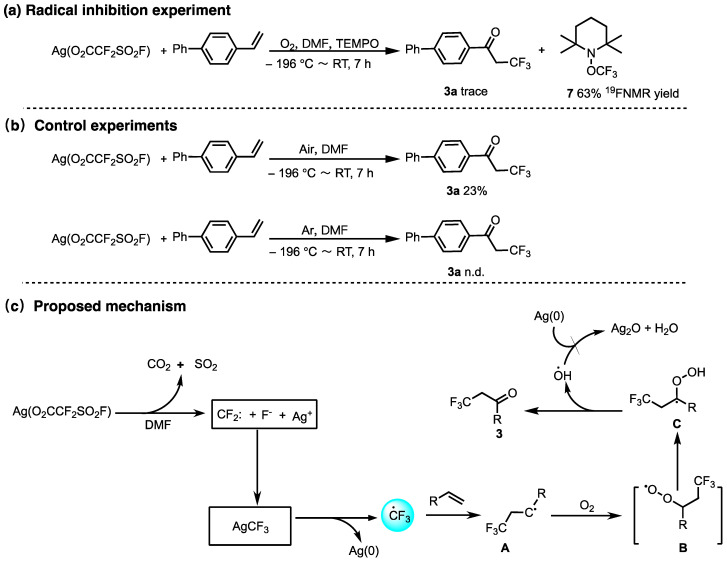
Control experiments and the proposed mechanism.

Next, several preliminary control experiments were performed to shed light on the mechanism of this transformation. The addition of a radical-trapping reagent TEMPO (2.0 equiv) to the reaction system led to a suppression of the formation of **3a** (Scheme 4a), while TEMPO-CF_3_ adduct **7** was detected by ^19^F NMR in 63% yield (see ESI, Figure S1). These observations suggested that the reaction might proceed via a free radical pathway. Subsequently, the role of O_2_ was investigated. As illustrated in Scheme 4b, the replacement of O_2_ with Air resulted in the desired product being obtained in a 23% ^19^F NMR yield. However, no target compound was generated in an Ar atmosphere. These results indicated that O_2_ was the source of the oxygen atom of the ketone product **3a**. In light of the aforementioned results and related mechanistic studies in the literature [22,23,27], it is plausible to propose the following reaction mechanism, as illustrated in Scheme 4c. The reaction of Ag(O_2_CCF_2_SO_2_F) in DMF results in the generation of difluorocarbene, fluoride ions, silver ions, and the release of CO_2_ and SO_2_. The generation of AgCF_3_ is achieved through the combination of difluorocarbene and fluoride ions, facilitated by the action of silver ions. This subsequently leads to the homolytic cleavage of the molecule, resulting in the formation of trifluoromethyl radicals. The addition of the CF_3_ radical to olefin results in the formation of benzylic radical **A**, which can be trapped by molecular oxygen to yield product **3** via intermediates **B** and **C**.

## 3. Materials and Methods

### 3.1. General Information of Materials and Instruments

The NMR spectra were obtained on a 400 MHz spectrometer (Bruker, Bremen, Germany) using CDCl_3_ or Methanol-*d*_4_ as a deuterated solvent, with ^1^H, ^19^F, and ^13^C NMR at 400 MHz, 100 MHz, and 376 MHz, respectively. Chemical shifts were reported in parts per million (ppm) relative to residual chloroform (7.26 ppm) or TMS as an internal standard (δ_TMS_ = 0 ppm) for ^1^H and ^13^C NMR spectra and 1-methoxy-4-(trifluoromethoxy) benzene as an external standard (negative for upfield) for ^19^F NMR spectra. The following abbreviations were used to explain the multiplicities: s = singlet, d = doublet, t = triplet, q = quartet, and m = multiplet. The NMR yield was determined by ^19^F NMR using (^19^F NMR: δ −58.4 ppm) as an internal standard before working up the reaction. GC-MS (EI) data were determined on an Agilent 5975C (Santa Clara, CA, USA). All reagents were purchased from TCI (Shanghai, China), or Shanghai Aladdin Biochemical Technology Co. Ltd, or prepared as described in the literature. Solvents were freshly dried and degassed according to the purification handbook Purification of Laboratory Chemicals before use. Flash column chromatography was carried out using 230–400 mesh silica gel (SiliCycle, Quebec, QC, Canada).

### 3.2. General Procedure for the Synthesis of Compounds (***3a***–***3t***)

4-Vinylbiphenyl (36 mg, 0.2 mmol) and Ag(O_2_CCF_2_SO_2_F) (227.9 mg, 0.8 mmol) were added to an oven-dried sealed tube equipped with a magnetic stir bar under the O_2_ atmosphere. The mixture was cooled to −196 °C, and ultra-dry *N*,*N*-dimethylformamide (4.0 mL) was added via syringe under O_2_ atmosphere. The mixture was warmed to room temperature and stirred for 7 h. 1-Methoxy-4-(trifluoromethoxy) benzene was added to the reaction mixture as an internal standard, and the yield of the desired product was measured by ^19^F NMR. The reaction mixture was then subjected to filtration. The filtrate was washed with water (20 mL) and saturated sodium chloride aqueous solution (20 mL), which was then extracted with EtOAc. The organic layer was dried over anhydrous Na_2_SO_4_ and then filtered. The filtrate was evaporated under reduced pressure. The resulting crude material was purified by flash column chromatography on silica gel (PE/EA) to afford the corresponding product.

## 4. Conclusions

In conclusion, a novel methodology for the synthesis of a diverse array of synthetically crucial α-CF_3_ ketones was devised. The successful use of Ag(O_2_CCF_2_SO_2_F) as a trifluoromethyl source and O_2_ as an oxidant in 1,2-oxidative trifluoromethylation of activated or unactivated olefins was achieved under mild and practical conditions. This reaction is distinguished by its broad functional-group tolerance, mild reaction conditions, and the unique reactivity of the Ag(O_2_CCF_2_SO_2_F) agent. Further studies are currently underway with the objective of expanding the applicability of the present reaction system and developing other multi-component reactions through the radical cross-over mechanism.

## Figures and Tables

**Table 1:** Optimization of reaction conditions *^a^*.

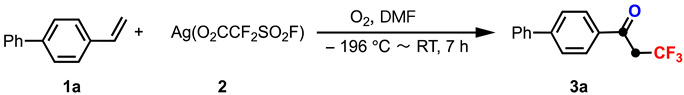		
Entry	Variation from Standard Conditions	Yield *^b^* (%)
1	None	87
2	MeCN, NMP, DMAc instead of DMF	<81
3	DCE, EtOAc, THF instead of DMF	n.d. *^c^*
4	Cu(O_2_CCF_2_SO_2_F)_2_ instead of Ag(O_2_CCF_2_SO_2_F)	n.d. *^c^*
5	TMSCF_3_/AgF system instead of Ag(O_2_CCF_2_SO_2_F)	33
6	2.0 equiv of Ag(O_2_CCF_2_SO_2_F)	40
7	3.0 equiv of Ag(O_2_CCF_2_SO_2_F)	49
8	5.0 equiv of Ag(O_2_CCF_2_SO_2_F)	89
9	−15 °C	23
10	0 °C	76
11	40 °C	56
12	60 °C	47
13	*m*-CPBA, NaIO_4_, K_2_S_2_O_8_, MnO_2_, DMP instead of O_2_	<45
14	Air	23
15	Ar	n.d. *^c^*

*^a^* General reaction conditions: **1a** (0.2 mmol, 1.0 equiv), Ag(O_2_CCF_2_SO_2_F) (0.8 mmol, 4.0 equiv), DMF (4 mL), O_2_ atmosphere, RT, 7 h. *^b^* Yields were determined by ^19^F NMR spectroscopy using (0.2 mmol, 1.0 equiv) 1-methoxy-4-(trifluoromethoxy)benzene as an internal standard. *^c^* n.d. = not detected.

## Data Availability

The data underlying this study are available in the published article and its Supporting Information.

## Supplementary Materials

The following supporting information can be downloaded at: https://www.mdpi.com/article/10.3390/molecules29235622/s1, including the full experimental details, characterization data, and copies of NMR spectra for new compounds (PDF format). References [5,15,19,28,29,30,31,32] are cited in the Supplementary Materials.
