# Supplementary material for: 1,2-Oxidative Trifluoromethylation of Olefin with Ag(O2CCF2SO2F) and O2: Synthesis of α-Trifluoromethyl Ketones
Source: Molecules. 2024 Nov 27;29(23):5622. doi: 10.3390/molecules29235622 (PMC11643604; doi:10.3390/molecules29235622)

1,2-Oxidative Trifluoromethylation of Olefin with $Ag(O_2CCF_2SO_2F)$ and O_2: Synthesis of α-Trifluoromethyl Ketones

Shengxue Zhang,[a] Wangchuan Xiao,[b] Jingjing Wu,[a,b] Fanhong Wu,[a,b] Houjin Huang,[a] Xiaoyu Ma,[a,b,*] Yafei Shi,[a,] Chao Liu[a,b,c,*]

[a]School of Chemical and Environmental Engineering, Shanghai Institute of Technology, 100 Haiquan Road, Shanghai 201418, China

[b]Shanghai-Sanming Engineering Research Center of Green Fluoropharmaceutical Technology, 25 Jingdong Road, Sanming City, Fujian 365004, China

[c]Key Laboratory of Fluorine and Nitrogen Chemistry and Advanced Materials, Shanghai Institute of Organic Chemistry, University of Chinese Academy of Sciences, Chinese Academy of Sciences, 345 Lingling Road, Shanghai 200032, China

* Xiaoyu Ma (X.Y. M.): maxiaoyu@sit.edu.cn; Chao Liu (C. L.): chaoliu@sit.edu.cn

Table of contents

I. Screening reaction conditions S2

II. General Procedure for the Synthesis of $Ag(O_2CCF_2SO_2F)$ S5

III. Analytical data for compounds 3a-3t S6

IV. Scale-up and derivatization reactions of trifluoromethyl ketone (3a) S15

V. Preliminary mechanistic studies S19

VI. References S21

VII. Copies of ^1H, ^{19}F and ^{13}C NMR spectra of products S22

I. Screening reaction conditions

Table S1. Screening various oxidant[a]

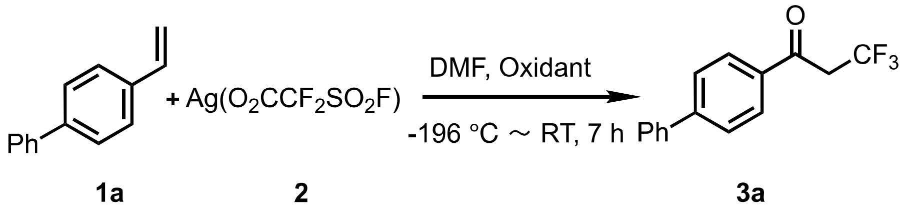

**Entry**	**Oxidant**	**Yield** (%)[b]
1	PIFA	13
2	$PhI(OAC)_2$	49
3	mCPBA	20
4	$NaIO_4$	ND.
5	$K_2S_2O_8$	32
6	MnO_2	28
7	DMP	58
8	O_2	87

[a]General reaction conditions: 1a (0.2 mmol, 1.0 equiv), $Ag(O_2CCF_2SO_2F)$ (0.8 mmol, 4.0 equiv), Oxidant (0.4 mmol, 2.0 equiv), DMF (4 mL), air atmosphere, RT, 7 h. [b]Yields were determined by ^{19}F NMR spectroscopy using 1-methoxy-4-(trifluoromethoxy) benzene as an internal standard.

Table S2. Screening various solvent[a]

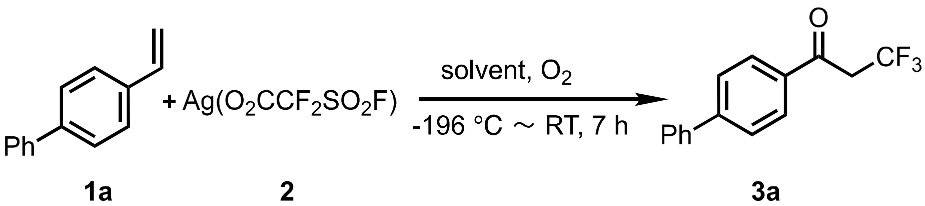

**Entry**	**Solvent**	**Yield (%)**[b]
1	THF	n.d.
2	EA	n.d.
3	DCE	n.d.
4	DMF	87
5	MeCN	46

6	NMP	74
7	DMAC	81

[a]General reaction conditions: 1a (0.2 mmol, 1.0 equiv), $Ag(O_2CCF_2SO_2F)$ (0.8 mmol, 4.0 equiv), solvents (4 mL), O_2 atmosphere, RT, 7 h. [b]Yields were determined by ^{19}F NMR spectroscopy using 1-methoxy-4-(trifluoromethoxy) benzene as an internal standard. n.d. = not detected.

Table S3. Screening various temperature[a]

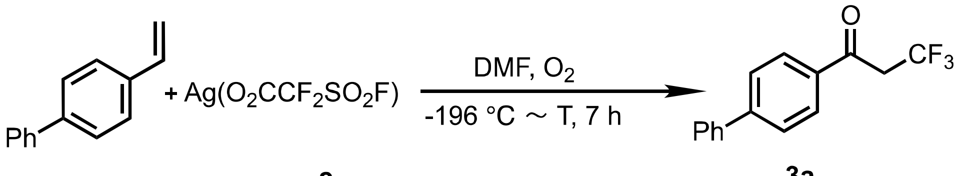

**Entry**	**Temperature (℃)**	**Yield** (%)[b]
1	-15	49
2	0	76
3	RT	87
4	30	68
5	40	56
6	60	47

[a]General reaction conditions: 1a (0.2 mmol, 1.0 equiv), $Ag(O_2CCF_2SO_2F)$ (0.8 mmol, 4.0 equiv), DMF (4 mL), O_2 atmosphere, temperature, 7 h. [b]Yields were determined by ^{19}F NMR spectroscopy using 1-methoxy-4-(trifluoromethoxy) benzene as an internal standard.

Table S4. Screening on the equivalent of $Ag(O_2CCF_2SO_2F)$ [a]

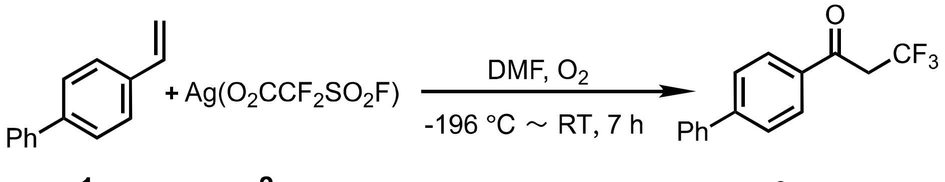

Entry	1a	2	**Yield** (%)[b]
1	1.0 eq. 0.2 mmol	2.0 eq. 0.4 mmol	40
2	1.0 eq. 0.2 mmol	3.0 eq. 0.6 mmol	49
3	1.0 eq. 0.2 mmol	4.0 eq. 0.8 mmol	87
4	1.0 eq. 0.2 mmol	5.0 eq. 1.0 mmol	89

[a]General reaction conditions: **1a** (0.2 mmol, 1.0 equiv), $Ag(O_2CCF_2SO_2F)$, DMF (4 mL), O_2 atmosphere, RT, 7 h. [b]Yields were determined by ^{19}F NMR spectroscopy using 1-methoxy-4-(trifluoromethoxy)benzene as an internal standard.

Table S5. Screening reaction time[a]

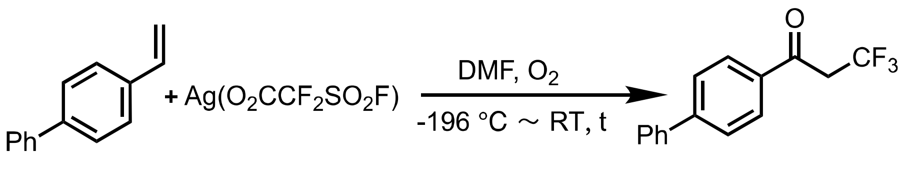

Entry	Time (h)	**Yield** (%)[b]
1	3	79
2	5	84
3	7	87
4	9	75
5	11	54

[a]General reaction conditions: **1a** (0.2 mmol, 1.0 equiv), $Ag(O_2CCF_2SO_2F)$ (0.8 mmol, 4.0 equiv), DMF (4 mL), O_2 atmosphere, RT, Time. [b]Yields were determined by ^{19}F NMR spectroscopy using 1-methoxy-4-(trifluoromethoxy)benzene as an internal standard.

II. General Procedure for the Synthesis of $Ag(O_2CCF_2SO_2F)$

$$FSO_2CF_2COOH + Ag_2CO_3 \xrightarrow[\text{Overnight}]{Et_2O,\ RT} Ag(FSO_2CF_2COO)$$

To a 100 mL two-necked bottle containing a magnetic stir bar were added Ag_2CO_3 (10 g, 36.26 mmol) and diethyl ether (30 mL). The two-necked bottle was installed with a cold finger condenser and a constant pressure funnel. Freshly distilled $FSO_2CF_2CO_2H$ (12.92 g, 72.52 mmol) was added dropwise via constant pressure funnel. The mixture was stirred vigorously at room temperature overnight. The mixture was filtered and washed with Et_2O (3 × 5 mL). The Et_2O layers were concentrated in vacuo to give a white solid (19.7 g, 95% yield). $Ag(O_2CCF_2SO_2F)$ was stored under nitrogen in the dark.

III. Analytical data for compounds 3a-3t

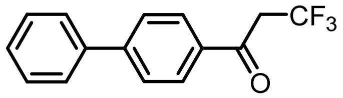

1-([1,1'-biphenyl]-4-yl)-3,3,3-trifluoropropan-1-one (**3a**): Obtained as a white solid in 72% yield (114.0 mg) by silica gel flash column chromatography eluted with PE/EA=50:1 (v/v). **^{1}H NMR** (400 MHz, Chloroform-*d, δ* ppm) 8.01 (d, *J* = 8.2 Hz, 2H), 7.73 (d, *J* = 8.2 Hz, 2H), 7.64 (d, *J* = 7.5 Hz, 2H), 7.48 (d, *J* = 4.5 Hz, 2H), 7.45 – 7.39 (m, 1H), 3.83 (q, 2H); **^{19}F NMR** (376 MHz, Chloroform-*d, δ* ppm) -61.89 (t, *J* = 10.0 Hz); **^{13}C NMR** (101 MHz, Chloroform-*d, δ* ppm) 189.3, 146.9, 139.4, 134.5, 129.1, 129.0, 128.6, 127.5, 127.3, 124.1 (q, *J* = 278.7. Hz), 42.2 (q, *J* = 28.2 Hz); **GCMS** (EI) $[M]^+$(m/z): calcd. for $[C_{15}H_{11}F_3O]^+$, 264.0, found 264.0. The analytical data are consistent with literature values [1].

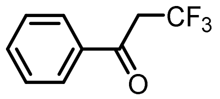

3,3,3-trifluoro-1-phenylpropan-1-one (**3b**): Obtained as a white solid in 51% yield (57.5 mg) by silica gel flash column chromatography eluted with PE/EA=50:1 (v/v). **^{1}H NMR** (400 MHz, Chloroform-*d, δ* ppm) 7.93 (d, *J* = 7.8 Hz, 2H), 7.64 (t, *J* = 7.6 Hz, 1H), 7.51 (t, *J* = 6.7 Hz, 2H), 3.80 (q, *J* = 10.0 Hz, 2H); **^{19}F NMR** (376 MHz, Chloroform-*d, δ* ppm) -62.03 (t, *J* = 10.0 Hz); **^{13}C NMR** (101 MHz, Chloroform-*d, δ* ppm) 189.7, 135.8, 134.2, 128.9, 128.3, 124.0 (q, *J* = 277.75 Hz), 42.1 (q, *J* = 28.28 Hz); **GCMS** (EI) $[M]^+$(m/z): calcd. for $[C_9H_7F_3O]^+$, 188.0, found 188.0. The analytical data are consistent with literature values [1].

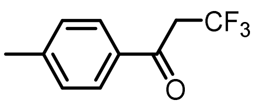

3,3,3-trifluoro-1-(p-tolyl)propan-1-one (**3c**): Obtained as a White solid in 68 % yield (82.4 mg) by silica gel flash column chromatography eluted with PE/EA=25:1 (v/v). **^{1}H NMR** (400 MHz, Chloroform-*d, δ* ppm) 7.83 (d, *J* = 8.0 Hz, 2H), 7.30 (d, *J* = 8.0 Hz, 2H),3.81- 3.72 (m, 2H), 2.43 (s, 3H); **^{19}F NMR** (376 MHz, Chloroform-*d, δ* ppm) -61.99 (t, *J* = 10.0 Hz); **^{13}C NMR** (101 MHz, Chloroform-*d, δ* ppm) 189.5, 145.3, 133.6, 129.6, 128.5, 124.1 (q, *J* = 277.75Hz), 42.4 (q, *J* = 28.2 Hz), 21.7; **GCMS** (EI) $[M]^+$(m/z): calcd. for $[C_{10}H_9F_3O]^+$, 202.0, found 202.0. The analytical data are consistent with literature values[1].

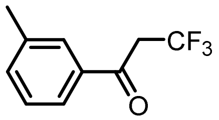

3,3,3-trifluoro-1-(m-tolyl)propan-1-one (**3d**): Obtained as a yellow oil in 61% yield (73.9 mg) by silica gel flash column chromatography eluted with PE/EA=50:1 (v/v). **^{1}H NMR** (400 MHz, Chloroform-*d, δ* ppm) 7.79 – 7.66 (m, 2H), 7.44 (d, *J* = 8.0 Hz, 1H), 7.40 (t, *J* = 7.6 Hz, 1H), 3.78 (q, *J* = 10.0 Hz, 2H), 2.43 (s, 3H); **^{19}F NMR** (376 MHz, Chloroform-*d, δ* ppm) -62.03 (t, J = 10.1 Hz); **^{13}C NMR** (101 MHz, Chloroform-*d, δ* ppm) 190.2, 138.9, 135.9, 135.0, 134.3, 128.8 (d, *J* = 4.0 Hz), 125.6, 124.0 (q, *J* = 276.74 Hz), 42.16 (q, *J* = 29.29 Hz), 21.3; **GCMS** (EI) $[M]^+$(m/z): calcd. for $[C_{10}H_9F_3O]^+$, 202.0, found 202.0. The analytical data are consistent with literature values[1].

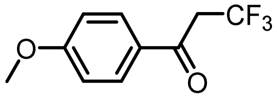

3,3,3-trifluoro-1-(4-methoxyphenyl)propan-1-one (**3e**): Obtained as a yellow solid in 62% yield (81.0 mg) by silica gel flash column chromatography eluted with PE/EA=20:1 (v/v). **^{1}H NMR** (400 MHz, Chloroform-*d, δ* ppm) 7.91 (d, *J* = 8.8 Hz, 2H), 8.01 – 7.79 (m, 2H), 6.96 (d, *J* = 8.8 Hz, 2H), 3.89 (s, 3H), 3.73 (q, *J* = 10.1 Hz, 2H); **^{19}F NMR** (376 MHz, Chloroform-*d, δ* ppm) -61.94 (t, *J* = 10.1 Hz); **^{13}C NMR** (101 MHz, Chloroform-*d, δ* ppm) 188.1, 164.4, 140.0, 130.8, 124.1 (q, *J* = 277.75Hz), 114.1, 55.6, 41.8 (q, *J* = 28.28 Hz); **GCMS** (EI) $[M]^+$(m/z): calcd. for $[C_{10}H_9F_3O_2]^+$, 218.0, found 218.0. The analytical data are consistent with literature values[2].

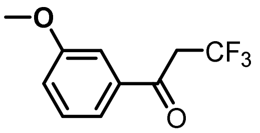

3,3,3-trifluoro-1-(3-methoxyphenyl)propan-1-one (**3f**): Obtained as a yellow oil in 56% yield (73.2 mg) by silica gel flash column chromatography eluted with PE/EA=50:1 (v/v). **^{1}H NMR** (400 MHz, Chloroform-*d, δ* ppm) δ 7.48 (d, *J* = 8.4 Hz, 2H), 7.41 (t, *J* = 8.0 Hz, 1H), 7.17 (d, *J* = 6.8 Hz, 1H), 3.86 (s, 3H), 3.78 (q, *J* = 10.1 Hz, 2H) **^{19}F NMR** (376 MHz, Chloroform-*d, δ* ppm) -61.95 (t, *J* = 10.1 Hz); **^{13}C NMR** (101 MHz, Chloroform-*d, δ* ppm) 189.6, 160.2, 137.3, 130.0, 124.1 (q, *J* = 277.75 Hz), 121.0, 120.8, 112.6, 55.6, 42.3 (q, *J* = 28.28 Hz); **GCMS** (EI) $[M]^+$(m/z): calcd. for $[C_{10}H_9F_3O_2]^+$, 218.0, found 218.0. The analytical data are consistent with literature values[3].

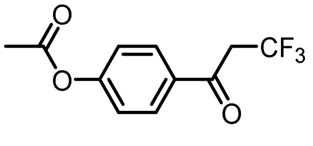

4-(3,3,3-trifluoropropanoyl)phenyl acetate (**3g**): Obtained as a White solid in 71 % yield (104.8 mg) by silica gel flash column chromatography eluted with PE/EA=25:3

(v/v). ^{1}**H NMR (**400 MHz, Chloroform-*d, δ* ppm) 8.01 (d, *J* = 8.6 Hz, 2H), 7.29 (d, *J* = 8.6 Hz, 2H), 3.81 (q, *J* = 10.0 Hz, 2H), 2.37 (s, 3H); ^{19}**F NMR** (376 MHz, Chloroform-*d, δ* ppm) -61.98 (t, *J* = 9.9 Hz); ^{13}**C NMR** (101 MHz, Chloroform-*d, δ* ppm) 188.8, 168.9, 155.5, 133.6, 130.3, 124.2 (q, *J* = 277.75 Hz), 122.4, 42.3 (q, *J* = 29.6 Hz), 21.3; **GCMS** (EI) $[M]^+$(m/z): calcd. for $[C_{11}H_9F_3O_3]^+$, 246.0, found 246.0. The analytical data are consistent with literature values [2].

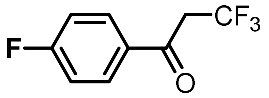

3,3,3-trifluoro-1-(4-fluorophenyl)propan-1-one (**3h**)**:** Obtained as a colorless oil in 52% yield (64.3 mg) by silica gel flash column chromatography eluted with PE/EA=50:1 (v/v). ^{1}**H NMR** (400 MHz, Chloroform-*d, δ* ppm) 7.97 (dd, *J* = 8.6, 5.3 Hz, 2H), 7.19 (t, *J* = 8.4 Hz, 2H), 3.77 (q, *J* = 10.0 Hz, 2H); ^{19}**F NMR** (376 MHz, Chloroform-*d, δ* ppm) -62.00 (t, *J* = 10.0 Hz), -102.88 (s); ^{13}**C NMR** (101 MHz, Chloroform-*d, δ* ppm) 188.2, 166.4 (d, *J* = 255.0 Hz), 132.3, 131.2 (d, *J* = 3.1 Hz), 124.1 (q, *J* = 270.6 Hz), 116.2 (d, *J* = 22.2 Hz), 42.1 (q, *J* = 28.1 Hz); **GCMS** (EI) $[M]^+$(m/z): calcd. for $[C_9H_6F_4O]^+$, 206.0, found 206.0. The analytical data are consistent with literature values[1].

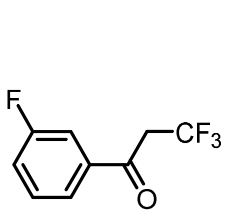

3,3,3-trifluoro-1-(3-fluorophenyl)propan-1-one (**3i**): Obtained as a colorless oil in 30 % yield (37.0 mg) by silica gel flash column chromatography eluted with PE/EA=50:1 (v/v). ^{1}**H NMR** (400 MHz, Chloroform-*d, δ* ppm) 7.71 (d, *J* = 7.8 Hz, 1H), 7.63 (d, *J* = 9.3 Hz, 1H), 7.56 – 7.45 (m, 1H), 7.34 (t, *J* = 8.2 Hz, 1H), 3.78 (q, *J* = 9.9

Hz, 2H); **^{19}F NMR** (376 MHz, Chloroform-*d,* *δ* ppm) -62.05 (t, *J* = 9.9 Hz), -110.85 (td, *J* = 8.7, 5.4 Hz); **^{13}C NMR** (101 MHz, Chloroform-*d,* *δ* ppm) 188.5, 161.7, 137.7, 130.7, (d, *J* = 8.08 Hz), 124.1 (d, *J* = 4.04 Hz), 123.8 (q, *J* = 277.75 Hz), 121.3 (d, *J* = 21.21 Hz), 115.1 (d, *J* = 22.22 Hz), 42.3 (q, *J* = 28.28 Hz); **GCMS** (EI) $[M]^+$(m/z): calcd. for $[C_9H_6F_4O]^+$, 206.0, found 206.0. The analytical data are consistent with literature values[3].

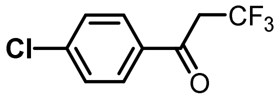

1-(4-chlorophenyl)-3,3,3-trifluoropropan-1-one (**3j**): Obtained as a White solid in 61% yield (81.2 mg) by silica gel flash column chromatography eluted with PE/EA=50:1 (v/v). **^{1}H NMR** (400 MHz, Chloroform-*d,* *δ* ppm) 7.87 (d, *J* = 6.6 Hz, 2H), 7.48 (d, *J* = 6.6 Hz, 2H), 3.77 (q, *J* = 9.8, Hz, 2H); **^{19}F NMR** (376 MHz, Chloroform-*d,* *δ* ppm) -61.97 (t, *J* = 9.9 Hz); **^{13}C NMR** (101 MHz, Chloroform-*d,* *δ* ppm) 188.7, 141.0, 134.2, 129.9, 129.4, 124.0 (q, *J* = 277.75 Hz), 42.3 (q, *J* = 28.28 Hz); **GCMS** (EI) $[M]^+$(m/z): calcd. for $[C_9H_6ClF_3O]^+$, 222.0, found 222.0. The analytical data are consistent with literature values[1].

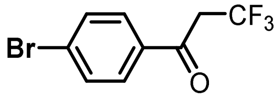

1-(4-bromophenyl)-3,3,3-trifluoropropan-1-one (**3k**): Obtained as a colorless oil in 56 % yield (89.4 mg) by silica gel flash column chromatography eluted with PE/EA=50:1 (v/v). **^{1}H NMR** (400 MHz, Chloroform-*d,* *δ* ppm) 7.79 (d, *J* = 8.6 Hz, 2H), 7.65 (d, *J* = 8.6 Hz, 2H), 3.76 (q, *J* = 9.9 Hz, 2H); **^{19}F NMR (**376 MHz, Chloroform-*d,* *δ* ppm) -61.97 (t, *J* = 9.9 Hz); **^{13}C NMR** (101 MHz, Chloroform-*d,* *δ* ppm) 188.8, 134.5, 132.3, 129.8, 129.7, 123.8 (q, *J* = 277.75 Hz), 42.1 (q, *J* = 28.28 Hz); **GCMS** (EI)

$[M]^+$(m/z): calcd. for $[C_9H_6BrF_3O]^+$, 266.0, found 266.0. The analytical data are consistent with literature values[1].

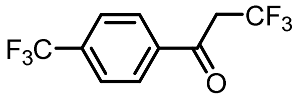

3,3,3-trifluoro-1-(4-(trifluoromethyl)phenyl)propan-1-one (**3l**): Obtained as a colorless oil in 56 % yield (86.0 mg) by silica gel flash column chromatography eluted with PE/EA=50:1 v/v. **^{1}H NMR** (400 MHz, Chloroform-*d, δ* ppm) 8.05 (d, *J* = 8.2 Hz, 2H), 7.78 (d, *J* = 8.2 Hz, 2H), 3.83 (q, *J* = 9.8 Hz, 2H); **^{19}F NMR** (376 MHz, Chloroform-*d, δ* ppm) -62.03 (t, *J* = 9.8 Hz), -63.35 (s); **^{13}C NMR** (101 MHz, Chloroform-*d, δ* ppm) 188.8, 138.3, 135.5 (q, *J* = 33.3 Hz), 128.7, 126.1 (q, *J* = 4.04 Hz), 123.7 (q, *J* = 277.75Hz), 123.4 (q, *J* = 275.75 Hz), 42.4 (q, *J* = 28.28 Hz); **GCMS** (EI) $[M]^+$(m/z): calcd. for $[C_{10}H_6F_6O]^+$, 256.0, found 256.0. The analytical data are consistent with literature values[1].

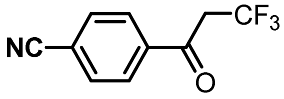

4-(3,3,3-trifluoropropanoyl)benzonitrile (**3m**)**:** Obtained as a white solid in 62 % yield (79.2 mg) by silica gel flash column chromatography eluted with PE/EA=10:1 (v/v). **^{1}H NMR** (400 MHz, Chloroform-*d, δ* ppm) 8.03 (d, *J* = 8.3 Hz, 2H), 7.82 (d, *J* = 8.2 Hz, 2H), 3.83 (q, *J* = 9.8 Hz, 2H); **^{19}F NMR** (376 MHz, Chloroform-*d, δ* ppm) -61.93 (t, *J* = 9.7 Hz); **^{13}C NMR** (101 MHz, Chloroform-*d, δ* ppm) 188.6, 138.5, 132.8, 128.8, 123.6 (q, *J* = 278.76Hz), 117.5, 116.0. 42.5 (q, *J* = 28.28 Hz); **GCMS** (EI) $[M]^+$(m/z): calcd. for $[C_{10}H_6F_3NO]^+$, 213.0, found 213.0. The analytical data are consistent with literature values[1].

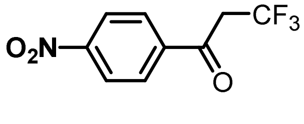

3,3,3-trifluoro-1-(4-nitrophenyl)propan-1-one (**3n**): Obtained as a yellow solid in 54 % yield (75.49 mg) by silica gel flash column chromatography eluted with PE/EA=10:1 (v/v). **^{1}H NMR** (400 MHz, Chloroform-*d, δ* ppm) 8.35 (d, *J* = 7.5 Hz, 2H), 8.11 (d, *J* = 7.8 Hz, 2H), 3.86 (q, *J* = 9.3 Hz, 2H); **^{19}F NMR (**376 MHz, Chloroform-*d, δ* ppm) -61.98 (q, *J* = 8.2 Hz); **^{13}C NMR** (101 MHz, Chloroform-*d, δ* ppm) 188.4, 150.9, 140.1, 129.4, 124.1, 123.2 (q, *J* = 276.7 Hz), 42.6 (q, *J* = 30.3 Hz); **GCMS** (EI) $[M]^+$(m/z): calcd. for $[C_9H_6F_3NO_3]^+$, 233.0, found 233.0. The analytical data are consistent with literature values[1].

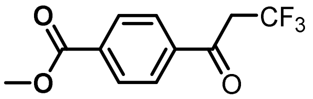

methyl 4-(3,3,3-trifluoropropanoyl)benzoate (**3o**): Obtained as a White solid in 67 % yield (98.9 mg) by silica gel flash column chromatography eluted with PE/EA=50:3 (v/v). **^{1}H NMR** (400 MHz, Chloroform-*d, δ* ppm) 8.16 (d, *J* = 8.6 Hz, 2H), 7.98 (d, *J* = 8.4 Hz, 2H), 3.96 (s, 3H), 3.83 (q, *J* = 9.9 Hz, 2H); **^{19}F NMR** (376 MHz, Chloroform-*d, δ* ppm) -62.02 (t, *J* = 9.7 Hz); **^{13}C NMR** (101 MHz, Chloroform-*d, δ* ppm) 189.4, 165.9, 138.8, 134.9, 130.1, 128.3, 123.8 (q, *J* = 277.75 Hz), 52.6, 42.4 (q, *J* = 28.28 Hz); **GCMS** (EI) $[M]^+$(m/z): calcd. for $[C_{11}H_9F_3O_3]^+$, 246.0, found 246.0. The analytical data are consistent with literature values[3].

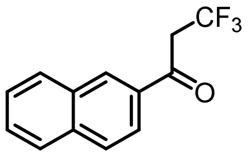

3,3,3-trifluoro-1-(naphthalen-2-yl)propan-1-one (**3p**): Obtained as a White solid in 41% yield (58.5 mg) by silica gel flash column chromatography eluted with PE/EA=10:1 (v/v). **^{1}H NMR** (400 MHz, Chloroform-*d, δ* ppm) 8.41 (s, 1H), 7.99 (m, 2H), 7.95 – 7.84 (m, 2H), 7.64 (m, 1H), 7.59 (m, 1H), 3.93 (q, *J* = 10.0 Hz, 2H); **^{19}F NMR** (376 MHz, Chloroform-*d, δ* ppm) -61.89 (t, *J* = 10.0 Hz); **^{13}C NMR** (101 MHz,

Chloroform-*d, δ* ppm) 189.6, 136.0, 133.2, 132.4, 130.5, 129.7, 129.2, 128.9, 127.9, 127.2, 124.9 (q, *J* = 277.7 Hz), 123.52, 42.2 (q, *J* = 28.2 Hz); **GCMS** (EI) $[M]^+$(m/z): calcd. for $[C_{13}H_9F_3O]^+$, 238.0, found 238.0. The analytical data are consistent with literature values[2].

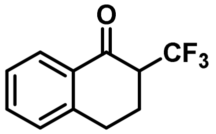

2-(trifluoromethyl)-3,4-dihydronaphthalen-1(2H)-one (**3q**): Obtained as a White solid in 28 % yield (36.0 mg) by silica gel flash column chromatography eluted with PE/EA=50:1 (v/v). **^{1}H NMR** (400 MHz, Chloroform-*d, δ* ppm) 8.08 (d, *J* = 7.9 Hz, 1H), 7.55 (t, *J* = 7.5 Hz, 1H), 7.37 (t, *J* = 7.6 Hz, 1H), 7.30 (d, *J* = 7.5 Hz, 1H), 3.25 – 3.24 (m, 1H), 3.17 – 3.05(m, 2H), 2.56 – 2.49(m, 1H), 2.39 – 2.20 (m, 1H); **^{19}F NMR** (376 MHz, Chloroform-*d*, *δ* ppm) -67.54 (d, *J* = 8.7 Hz); **^{13}C NMR** (101 MHz, Chloroform-*d, δ* ppm) 190.2, 143.1, 134.2, 131.9, 128.8, 127.8, 127.1, 125.1 (q, *J* = 279.77 Hz;), 50.9 (q, *J* = 26.26 Hz), 27.5, 23.4 (q, *J* = 3.03 Hz); **GCMS** (EI) $[M]^+$(m/z): calcd. for $[C_{11}H_9F_3O]^+$, 214.0, found 214.0. The analytical data are consistent with literature values[4].

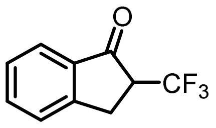

2-(trifluoromethyl)-2,3-dihydro-1H-inden-1-one (**3r**): Obtained as a white solid in 27 % yield (32.4 mg) by silica gel flash column chromatography eluted with PE/EA=10:1 (v/v). **^{1}H NMR** (400 MHz, Chloroform-*d, δ* ppm) 7.80 (d, *J* = 7.7 Hz, 1H), 7.66 (t, *J* = 7.5 Hz, 1H), 7.52 (d, *J* = 7.7 Hz, 1H), 7.43 (t, *J* = 7.5 Hz, 1H), 3.68 – 2.98 (m, 3H); **^{19}F NMR** (376 MHz, Chloroform-*d*, *δ* ppm) -67.76 (d, *J* = 9.3 Hz); **^{13}C NMR**

(101 MHz, Chloroform-*d*, *δ* ppm) 196.9, 152.1, 135.8,135.7, 128.2, 126.5, 124.7, 122.2 (q, *J* = 278.4 Hz), 49.8 (q, *J* = 27.3Hz）, 27.6 (d, *J* = 2 Hz); **GCMS** (EI) $[M]^+$(m/z): calcd. for $[C_{10}H_7F_3O]^+$, 200.0, found 200.0. The analytical data are consistent with literature values [4].

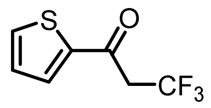

3,3,3-trifluoro-1-(thiophen-2-yl)propan-1-one (**3s**): Obtained as a colorless oil in 41 % yield (47.7 mg) by silica gel flash column chromatography eluted with PE/EA=10:1 (v/v) **^{1}H NMR** (400 MHz, Chloroform-*d*, *δ* ppm) 7.74 (m, *J* = 10.2, Hz, 2H), 7.18 (dd, *J* = 5.0, 3.9 Hz, 1H), 3.71 (q, *J* = 10.1 Hz, 2H); **^{19}F NMR** (376 MHz, Chloroform-*d*, *δ* ppm) -61.92 (t, *J* = 10.1 Hz); **GCMS** (EI) $[M]^+$(m/z): calcd. for $[C_7H_5F_3OS]^+$, 194.0, found 194.0. The analytical data are consistent with literature values [2].

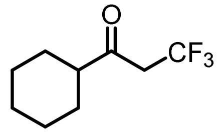

1-cyclohexyl-3,3,3-trifluoropropan-1-one (**3u**): **^{19}F NMR** (376 MHz, Chloroform-*d*, *δ* ppm) -62.76 (d, *J* = 9.3 Hz); **GCMS** (EI) $[M]^+$(m/z): calcd. for $[C_9H_{13}F_3O]^+$, 194.0, found 194.0. The analytical data are consistent with literature values[4].

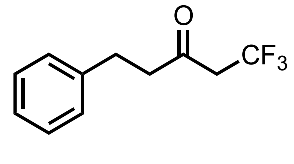

1,1,1-trifluoro-5-phenylpentan-3-one (**3v**): **^{19}F NMR** (376 MHz, Chloroform-*d*, *δ* ppm) -62.76 (d, *J* = 9.3 Hz); **GCMS** (EI) $[M]^+$(m/z): calcd. for $[C_{11}H_{11}F_3O]^+$, 216.0, found 216.0. The analytical data are consistent with literature values[8].

IV. Scale-up and derivatization reactions of trifluoromethyl ketone (3a)

1. Gram-scale synthesis of 1-([1,1'-biphenyl]-4-yl)-3,3,3-trifluoropropan-1-one (3a)

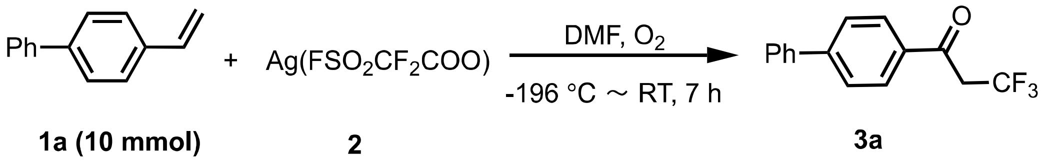

4-Vinylbiphenyl (1.80 g, 10.0 mmol) and $Ag(O_2CCF_2SO_2F)$ (11.40 g, 40.0 mmol) were added to an oven-dried sealed tube equipped with a magnetic stir bar under O_2 atmosphere. The mixture was cooled to -196 °C, ultra-dry *N*,*N*-dimethylformamide (40.0 mL) was added via syringe under Ar atmospher. The mixture was warmed to room temperature and stirred for 7 h. 1-methoxy-4-(trifluoromethoxy) benzene was added into the reaction mixture as an internal standard and the yield of the desired product was measured by ^{19}F NMR. The reaction mixture was then subjected to filtration. The filtrate was washed with water (20 mL) and saturated sodium chloride aqueous solution (20 mL), which was then extracted with EtOAc. The organic layer was dried over anhydrous Na_2SO_4 and then filtered. The filtrate was evaporated under reduced pressure. The resulting crude material was purified by flash column chromatography on silica gel (PE/EA) to afford the corresponding product (1.67 g, 63%).

2. Derivatization of 1-([1,1'-biphenyl]-4-yl)-3,3,3-trifluoropropan-1-one (3a)

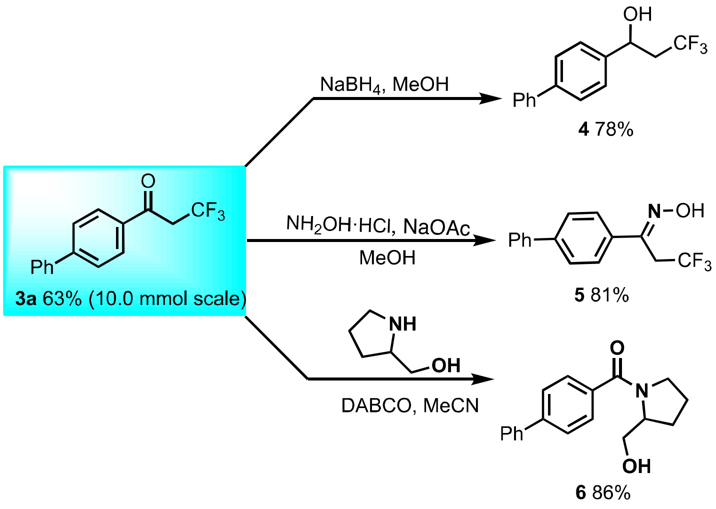

(a) The procedure for the preparation of 1-([1,1'-biphenyl]-4-yl)-3,3,3-trifluoropropan-1-ol (4)

A 10 mL schelenk equipped with a magnetic stirring bar was charged with **3a** (158.4 mg, 0.6 mmol), anhydrous methanol (1.0 mL), and then $NaBH_4$ (68 mg, 1.8 mmol) was added slowly. The reaction mixture was stirred vigorously at room temperature for 2 h. The residue was subjected to flash column chromatography to afford the desired product.

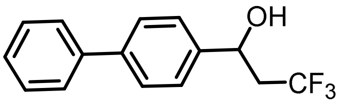

1-([1,1'-biphenyl]-4-yl)-3,3,3-trifluoropropan-1-ol (**4**): Obtained as a white solid in 78% yield (124.0 mg) by silica gel flash column chromatography eluted with PE/EA=10:1 v/v. **^{1}H NMR** (400 MHz, Chloroform-*d*, δ ppm) 7.65 – 7.56 (m, 4H), 7.51 – 7.34 (m, 5H), 5.13 (dd, J = 9.0, 3.6 Hz, 1H), 2.76 – 2.59 (m, 1H), 2.51 (m, 1H); **^{19}F NMR** (376 MHz, Chloroform-*d*, δ ppm) -63.62 (t, J = 10.6 Hz); **^{13}C NMR** (101 MHz, Chloroform-*d*, δ ppm) 141.42, 141.39, 140.56, 128.90, 127.59, 127.15, 126.19, 125.98 (q, J = 277.75 Hz) 68.62 (d, J = 3.03 Hz), 42.89 (q, J = 27.27Hz); GCMS (EI) [M]+(m/z): calcd. for $[C_{15}H_{13}F_3O]^+$, 266.0, found 266.0. The analytical data are consistent with literature values[5].

(b) The procedure for the preparation of 1-([1,1'-biphenyl]-4-yl)-3,3,3-trifluoropropan-1-one oxime (5)

A 10 mL sealed tube equipped with a magnetic stirring bar was charged with **3a** (158.40 mg, 0.6 mmol), hydroxylamine hydrochloride (125.08 mg, 1.8 mmol), NaOAc (64.77 mg, 0.78 mmol), and anhydrous methanol (1.0 mL). The reaction mixture was stirred vigorously at 60 ℃ for 3 h. The residue was subjected to flash column chromatography to afford the desired product.

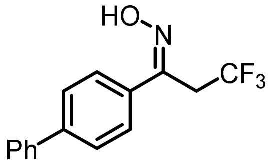

1-([1,1'-biphenyl]-4-yl)-3,3,3-trifluoropropan-1-one oxime (5): Obtained as a yellow solid in 81% yield (135.0 mg) by silica gel flash column chromatography eluted with PE/EA=5:1 v/v. ^{1}H NMR (401 MHz, Methanol-*d*4, *δ* ppm) 11.62, 7.79 – 7.73 (m, 2H), 7.62 (dd, *J* = 8.3, 2.2 Hz, 4H), 7.42 (dd, *J* = 8.5, 6.9 Hz, 2H), 7.37 – 7.28 (m, 1H), 3.89 (d, *J* = 10.9 Hz, 1H), 3.83 (d, *J* = 10.9 Hz, 1H); ^{19}F NMR (377 MHz, Methanol-*d*4, *δ* ppm) -62.91 (t, *J* = 10.8 Hz); ^{13}C NMR (101 MHz, Methanol-*d*4, *δ* ppm) 147.29, 141.83, 140.21, 134.18, 128.57, 127.32, 126.56, 126.46, 125.18 (q, *J* = 277.75 Hz), 28.79 (q, *J* = 31.31 Hz). The analytical data are consistent with literature values[6].

(c) The procedure for the preparation of [1,1'-biphenyl]-4-yl(2-(hydroxymethyl)pyrrolidin-1-yl)methanone (6)

A 10 mL sealed tube equipped with a magnetic stirring bar was charged with **3a** (158.4 mg, 0.6 mmol), DABCO (134.6 mg, 1.2 mmol), Prolinol (182.07 mg, 1.8 mmol). The tube was then evacuated and backfilled with Ar (3 times) and ultra-dry acetonitrile (4 mL) was added via syringe under Ar atmosphere. The reaction mixture was stirred vigorously at 50 ℃ for 12 h. The residue was subjected to flash column chromatography to afford the desired product.

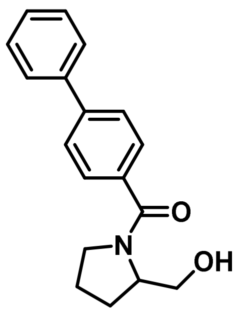

[1,1'-biphenyl]-4-yl(2-(hydroxymethyl)pyrrolidin-1-yl)methanone (7): Obtained as a yellow solid in 72% yield (121.0 mg) by silica gel flash column chromatography eluted with PE/EA=1:2 v/v. **^{1}H NMR** (400 MHz, Chloroform-*d, δ* ppm) 7.66 – 7.55 (m, 6H), 7.45 (dd, *J* = 8.3, 6.7 Hz, 2H), 7.41 – 7.33 (m, 1H), 4.48 – 4.37 (m, 1H), 3.82 (dd, *J* = 11.4, 2.7 Hz, 1H), 3.75 (dd, *J* = 11.5, 7.2 Hz, 1H), 3.63 – 3.55 (m, 1H), 3.58 – 3.47 (m, 1H), 3.35 (s, 1H), 2.18 (m, *J* = 12.8, 6.8, 5.7 Hz, 1H), 1.95 – 1.84 (m, 1H); **^{13}C NMR** (101 MHz, Chloroform-*d, δ* ppm) 172.05, 143.13, 140.19, 135.37, 128.94, 127.91, 127.72, 127.19, 127.06, 67.19, 61.58, 51.25, 28.56, 25.11. The analytical data are consistent with literature values [7].

V. Preliminary mechanistic studies

(1) Controlled experiment

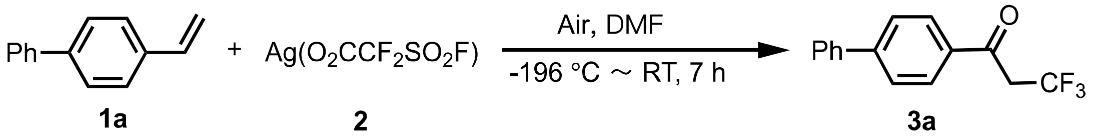

To an oven-dried sealed tube (10 mL) equipped with a magnetic stirring bar were added 4-vinylbiphenyl **1a** (0.2 mmol, 1.0 equiv.) and Ag($O_2CCF_2SO_2F$) (0.8 mmol, 4.0 equiv.). The mixture was cooled to -196 °C, ultra-dry *N*,*N*-dimethylformamide (4.0 mL) was added via syringe under Air atmosphere. The mixture was warmed to room temperature and stirred for 7 h under air. The crude reaction mixture was then analyzed by ^{19}F NMR spectroscopy with 1-methoxy-4-(trifluoromethoxy) benzene was added into the reaction mixture as an internal standard, and the yield of **3a** is 23%.

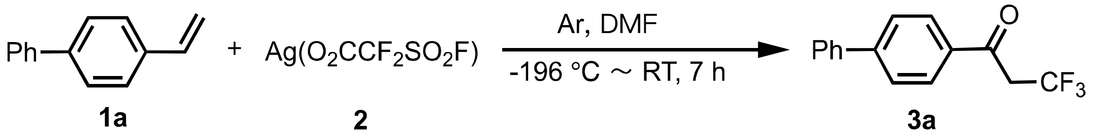

To an oven-dried sealed tube (10 mL) equipped with a magnetic stirring bar were added 4-vinylbiphenyl **1a** (0.2 mmol, 1.0 equiv.) and Ag($O_2CCF_2SO_2F$) (0.8 mmol, 4.0 equiv.) under Ar atmosphere. The mixture was cooled to -196°C, ultra-dry *N*,*N*-dimethylformamide (4.0 mL) was added via syringe under Ar atmosphere. The mixture was warmed to room temperature and stirred for 7 h. The crude reaction mixture was then analyzed by ^{19}F NMR spectroscopy with 1-methoxy-4-(trifluoromethoxy) benzene was added into the reaction mixture as an internal standard, and no target compound was obtained.

2. **Radical inhibition experiments**

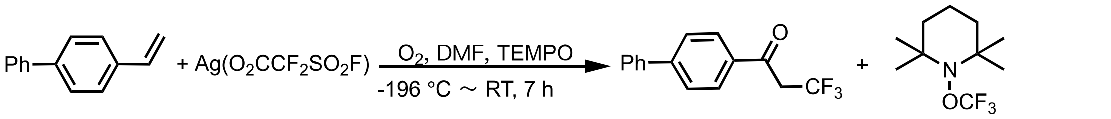

To an oven-dried sealed tube (10 mL) equipped with a magnetic stirring bar were added 4-vinylbiphenyl **1a** (0.2 mmol, 1.0 equiv.), $Ag(O_2CCF_2SO_2F)$ (0.8 mmol, 4.0 equiv.), TEMPO (0.4 mmol, 2.0 equiv.) under O_2 atmosphere. The mixture was cooled to -198 °C, ultra-dry *N*,*N*-Dimethylformamide (4.0 mL) was added via syringe under Ar atmosphere. The mixture was warmed to room temperature and stirred for 7 h under air. The crude reaction mixture was then analyzed by ^{19}F NMR spectroscopy with 1-methoxy-4-(trifluoromethoxy) benzene was added into the reaction mixture as an internal standard.

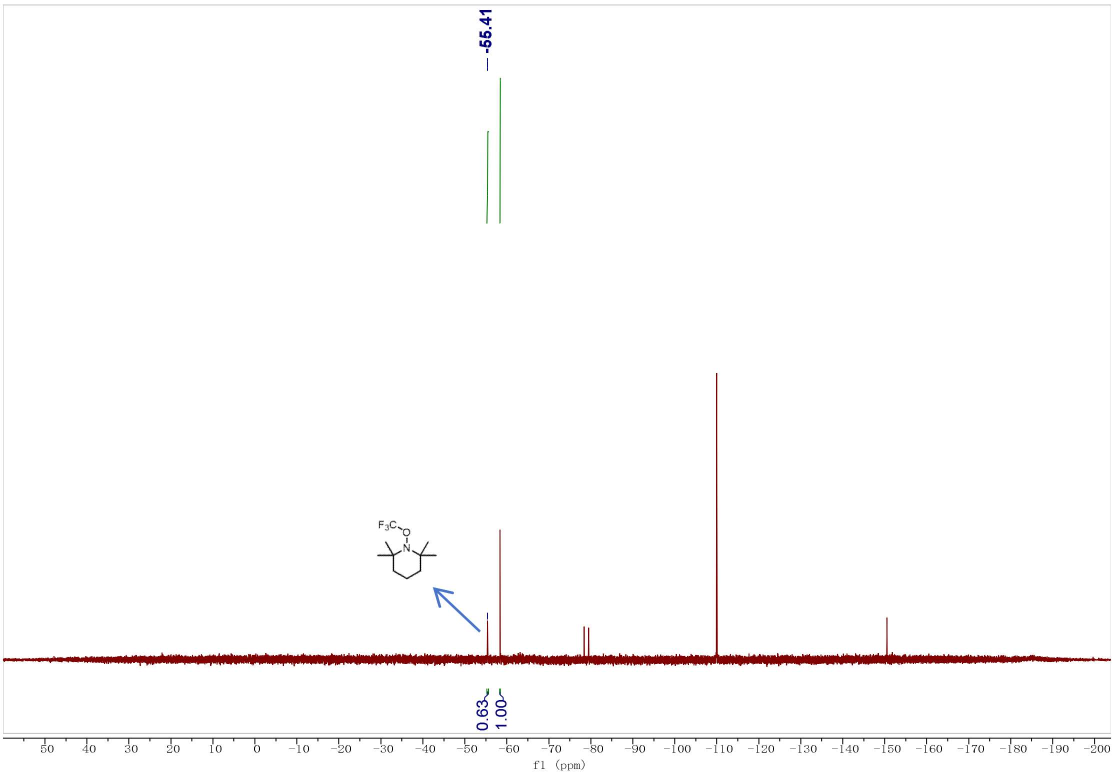

Figure S1 ^{19}F NMR spectroscopy of **7**

VI. References

1. Xu, J.; Li, Y.; Zhu, X.; Lv, S.; Xu, Y.; Cheng, T.; Liu, G.; Liu, R., Pyridinium-Masked Enol as a Precursor for Constructing Alpha-Fluoromethyl Ketones. *Org. Lett.* **2023,** *25*, 6211-6216.

2. Wu, Y. B.; Lu, G. P.; Yuan, T.; Xu, Z. B.; Wan, L.; Cai, C., Oxidative trifluoromethylation and fluoroolefination of unactivated olefins. *Chem. Commun.* **2016,** *52*, 13668-13670.

3. Li, Y.-J.; Liu, D.-G.; Ren, J.-H.; Gong, T.-J.; Fu, Y., Photocatalytic Alkyl Radical Addition Tandem Oxidation of Alkenyl Borates. *J. Org. Chem.* **2023,** *88*, 4325-4333.

4. Su, X.; Huang, H.; Yuan, Y.; Li, Y., Radical Desulfur-Fragmentation and Reconstruction of Enol Triflates: Facile Access to alpha-Trifluoromethyl Ketones. *Angew. Chem. Int. Ed.* **2017,** *56*, 1338-1341.

5. Li, Q.; Fan, W.; Peng, D.; Meng, B.; Wang, S.; Huang, R.; Liu, S.; Li, S., Cobalt–Tertiary-Amine-Mediated Hydroxytrifluoromethylation of Alkenes with CF3Br and Atmospheric Oxygen. *ACS Catal.* **2020,** *10*, 4012-4018.

6. Lu, K.; Wei, X.; Li, Q.; Li, Y.; Ji, L.; Hua, E.; Dai, Y.; Zhao, X., Synthesis of α-trifluoromethyl ethanone oximes via the three-component reaction of aryl-substituted ethylenes, tert-butyl nitrite, and the Langlois reagent. *Org. Chem. Front.* **2019,** *6*, 3766-3770.

7. Mao, K.; Lv, L.; Li, Z., Amine-Induced Selective C-C Bond Cleavage of 2,2,2-Trifluoroethyl Carbonyls for the Synthesis of Ureas and Amides. *J. Org. Chem.* **2023,** *88*, 10137-10146.

8. Gauthier, R.; Tzouras, N. V.; Nolan, S. P.; Paquin, J.-F., Exploiting the inductive effect of the trifluoromethyl group: regioselective gold-catalyzed hydration of 2,2,2-trifluoroethyl-substituted alkynes. *Chem. Commun.* **2023,** *59*, 9138-9141.

VII. Copies of ^{1}H, ^{19}F and ^{13}C NMR spectra of products

^{1}H NMR spectrum of 1-([1,1'-biphenyl]-4-yl)-3,3,3-trifluoropropan-1-one (**3a**) (400 MHz, Chloroform-*d*):

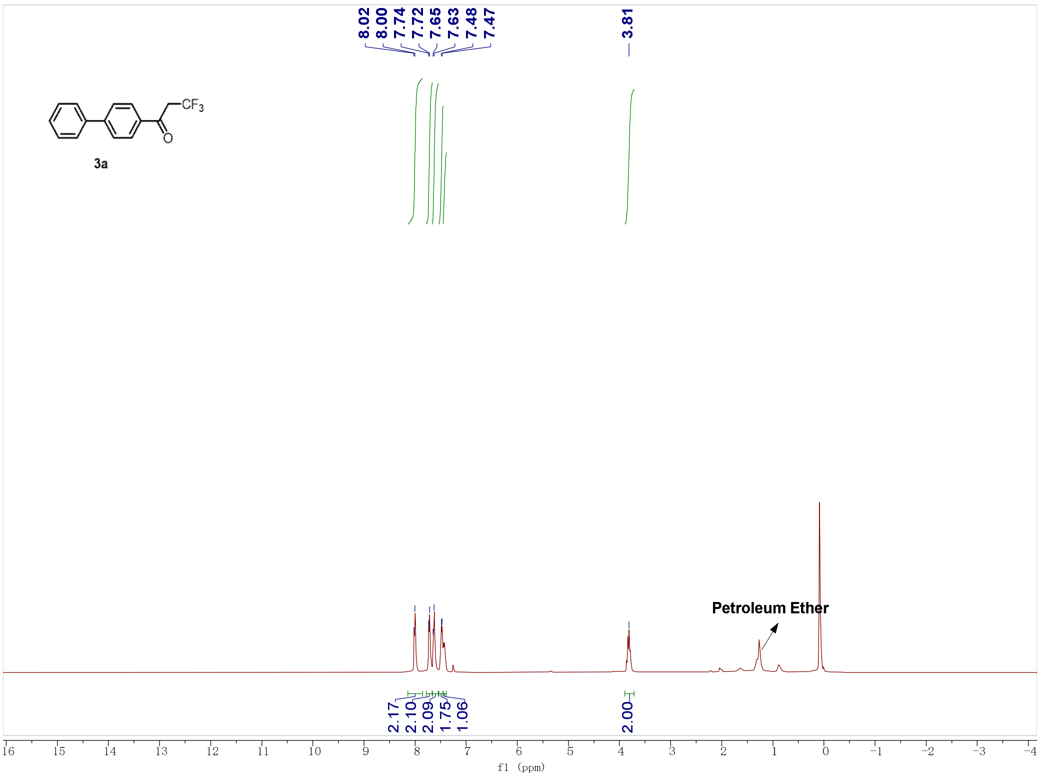

^{19}F NMR spectrum of 1-([1,1'-biphenyl]-4-yl)-3,3,3-trifluoropropan-1-one (**3a**) (376 MHz, Chloroform-*d*):

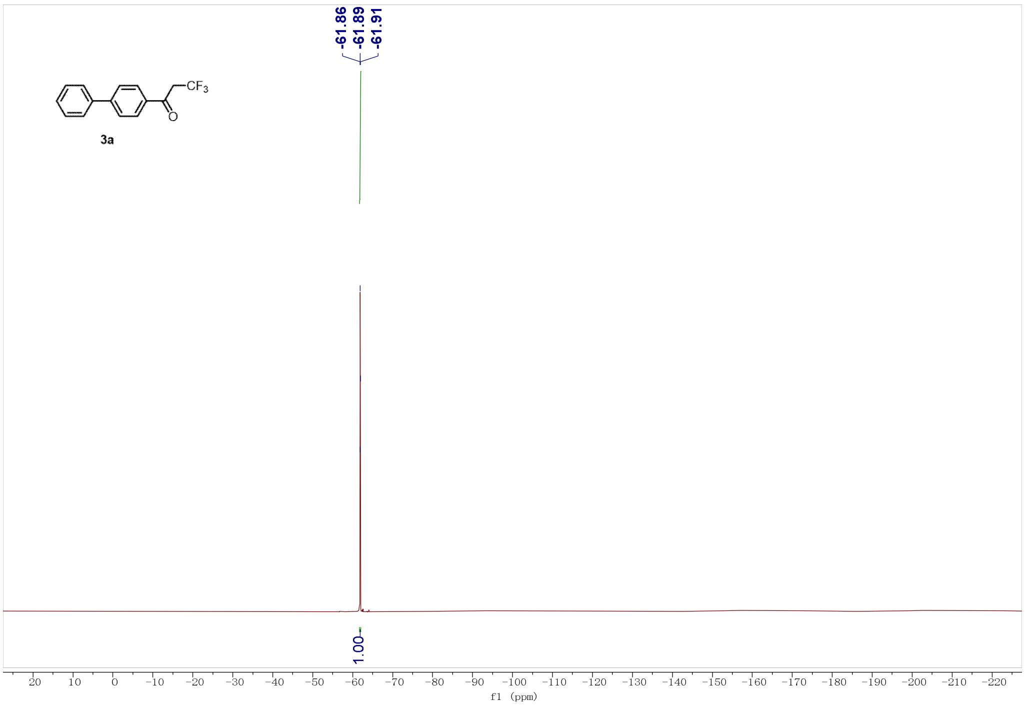

^{13}C NMR spectrum of 1-([1,1'-biphenyl]-4-yl)-3,3,3-trifluoropropan-1-one (**3a**) (101 MHz, Chloroform-*d*):

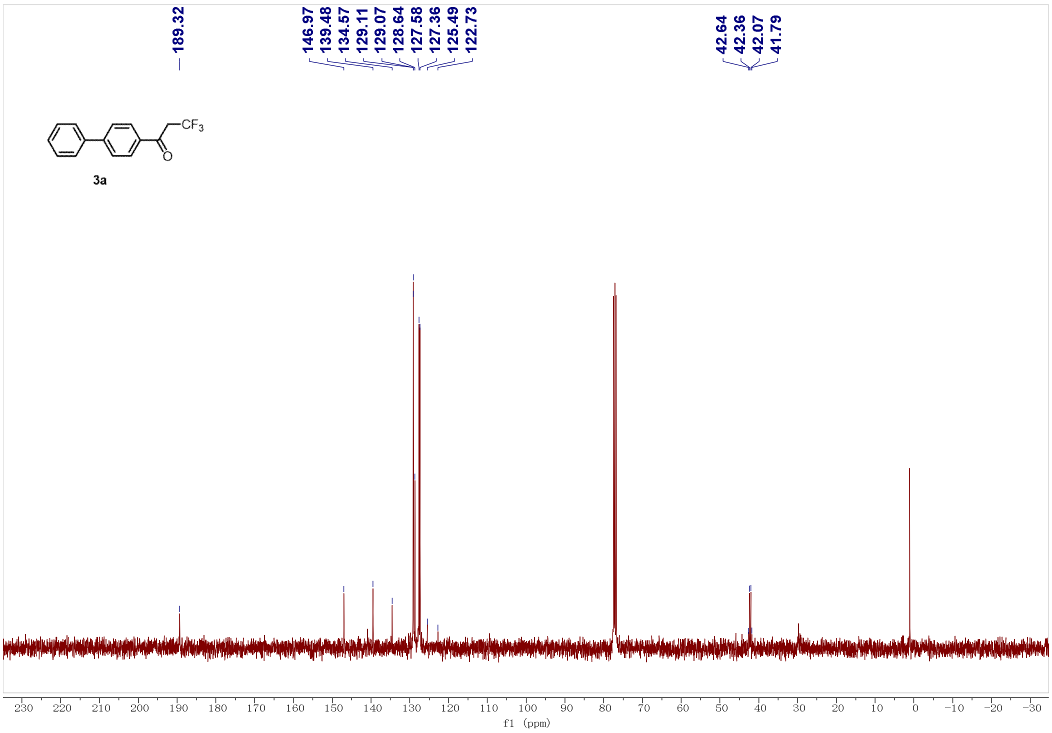

^{1}H NMR spectrum of 3,3,3-trifluoro-1-phenylpropan-1-one (**3b**) (400 MHz, Chloroform-*d*):

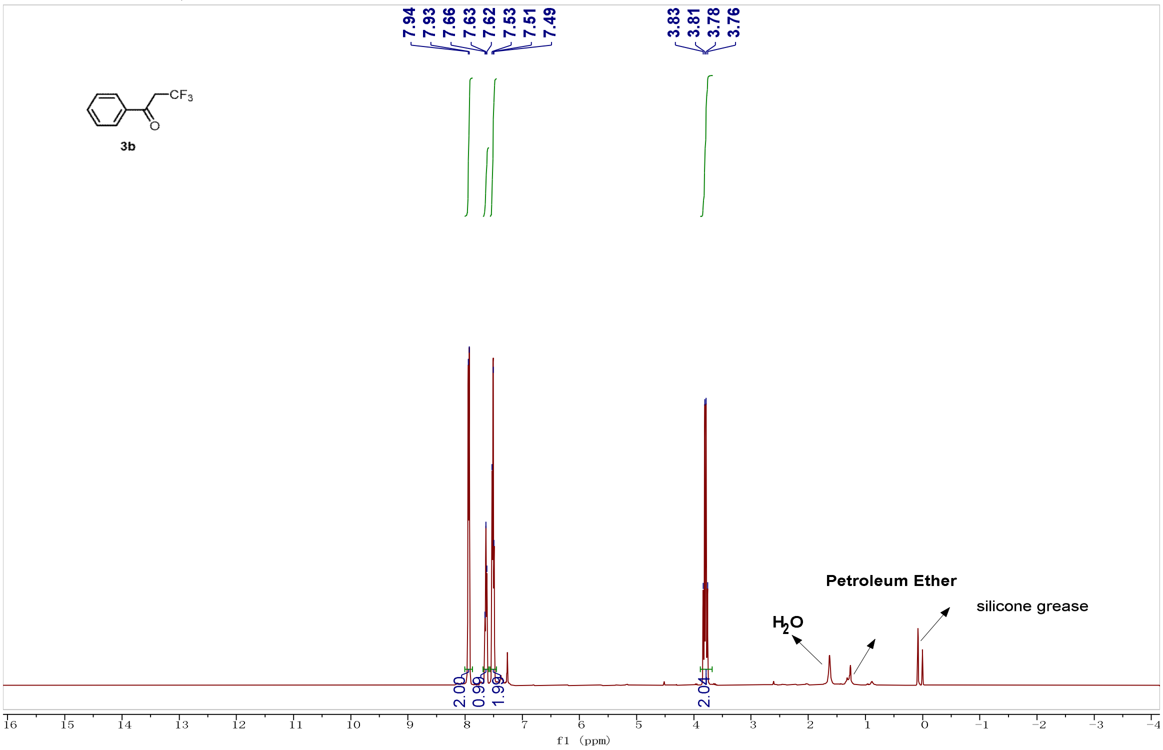

^{19}F NMR spectrum of 3,3,3-trifluoro-1-phenylpropan-1-one (**3b**) (376 MHz, Chloroform-*d*):

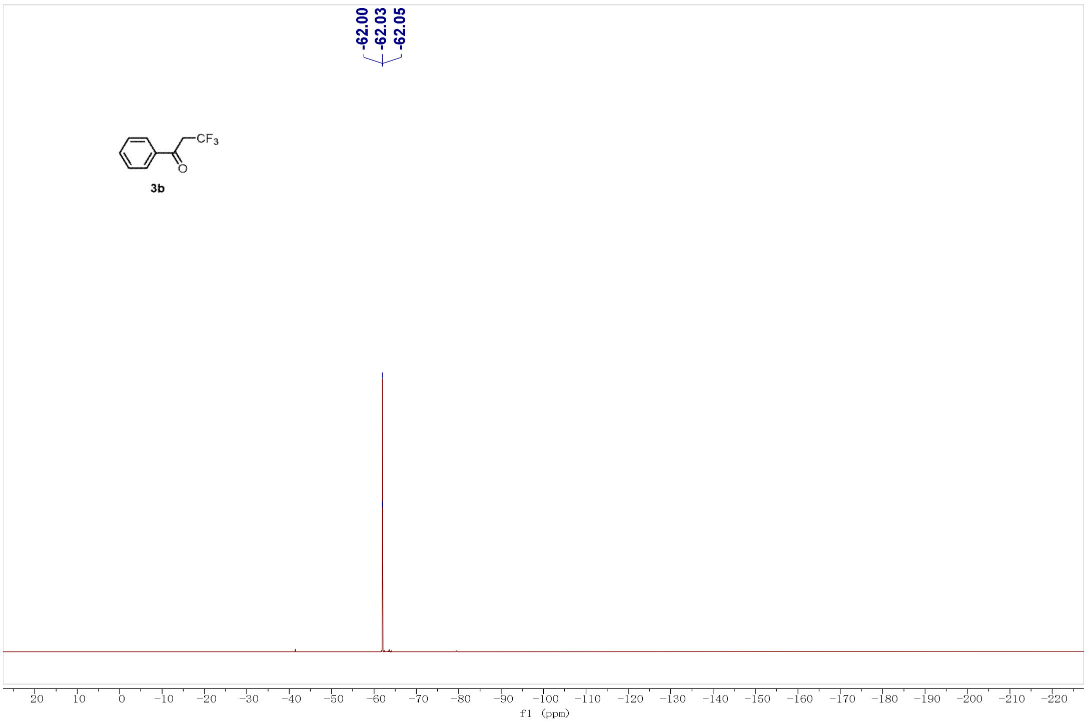

^{13}C NMR spectrum of 3,3,3-trifluoro-1-phenylpropan-1-one (**3b**) (101 MHz, Chloroform-*d*):

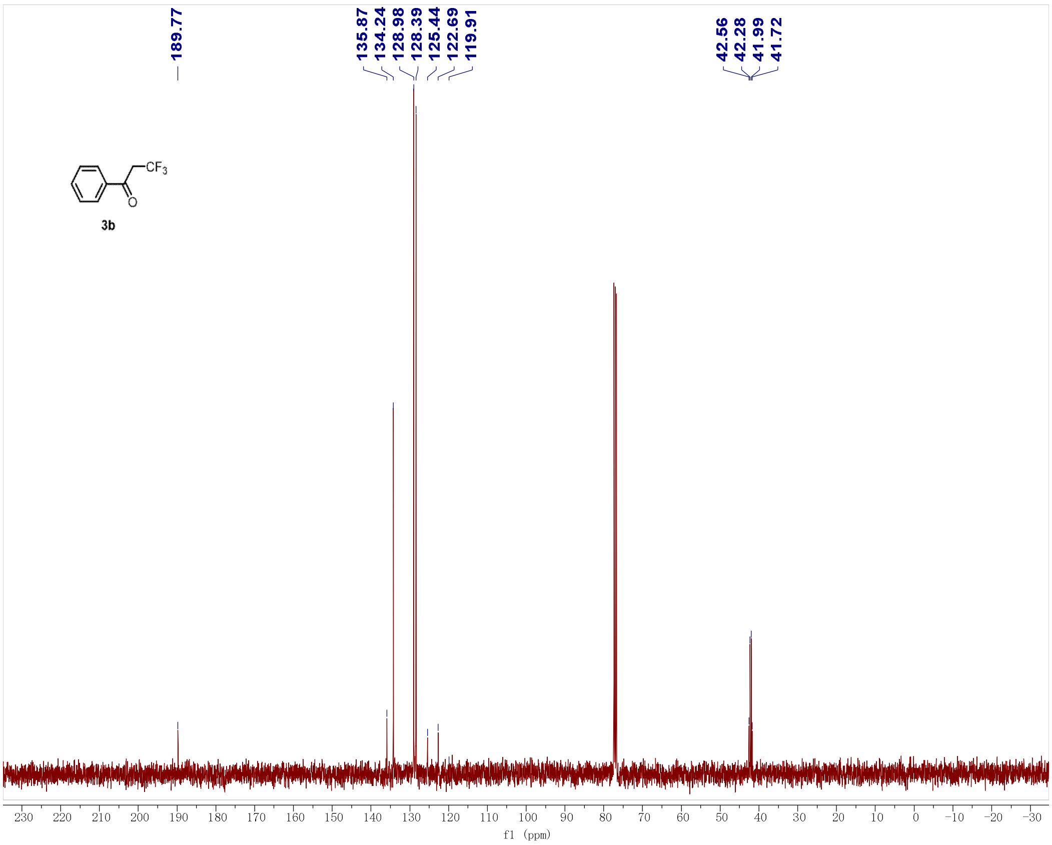

^1H NMR spectrum of 3,3,3-trifluoro-1-(p-tolyl)propan-1-one (**3c**) (400 MHz, Chloroform-*d*):

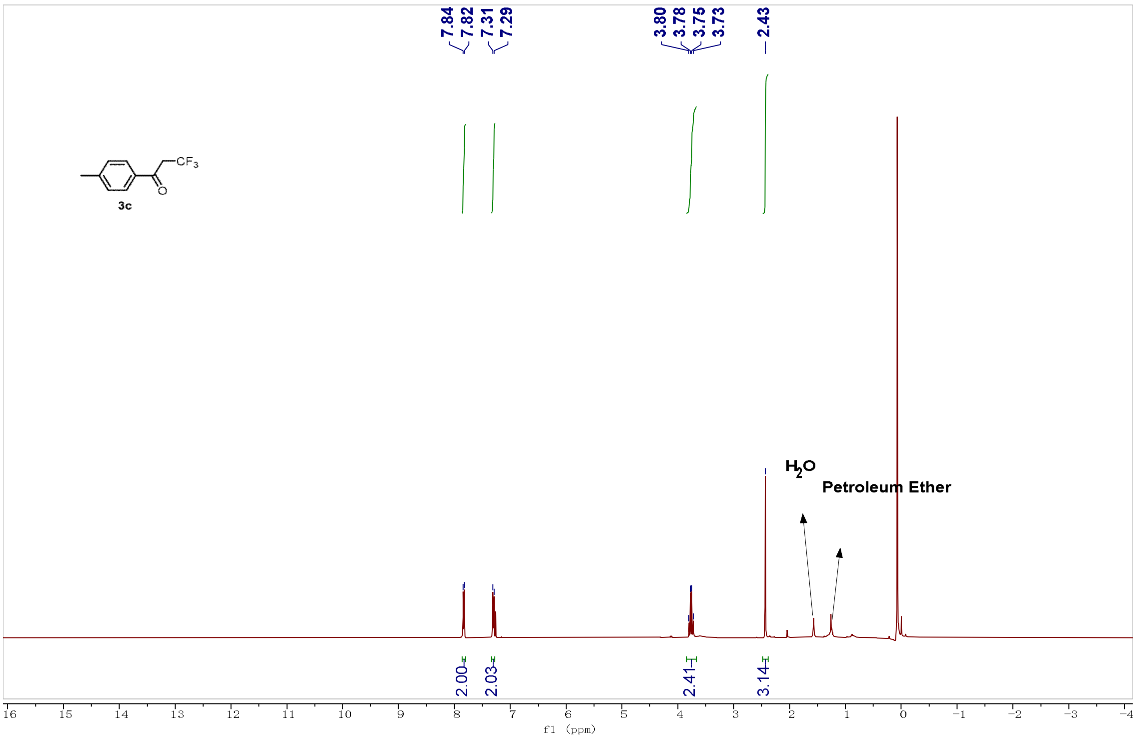

^{19}F NMR spectrum of 3,3,3-trifluoro-1-(p-tolyl)propan-1-one (**3c**) (376 MHz, Chloroform-*d*):

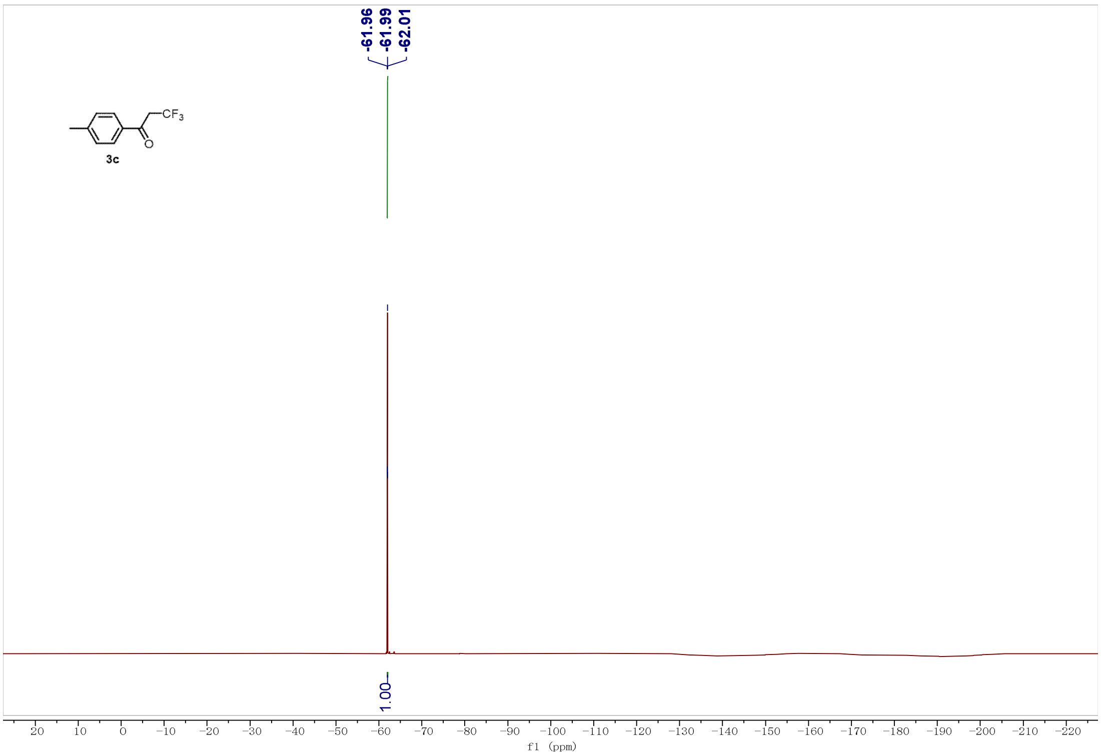

^{13}C NMR spectrum of 3,3,3-trifluoro-1-(p-tolyl)propan-1-one (**3c**) (101 MHz, Chloroform-*d*):

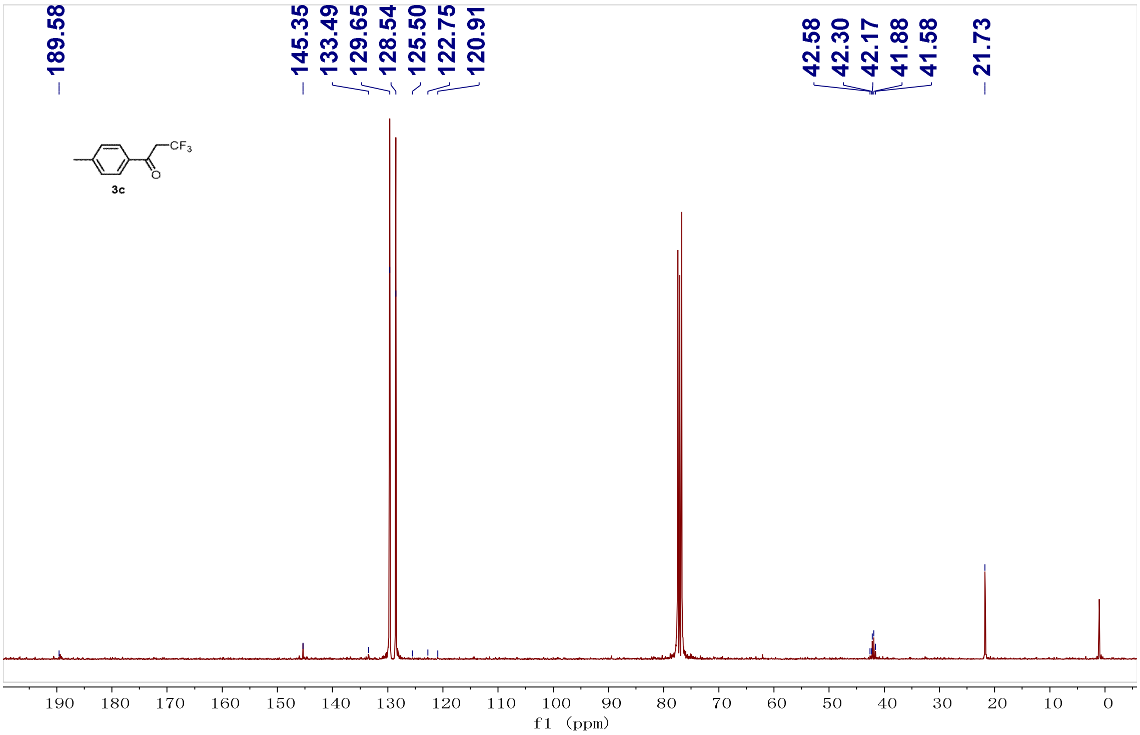

^{1}H NMR spectrum of 3,3,3-trifluoro-1-(m-tolyl)propan-1-one (**3d**) (400 MHz, Chloroform-*d*):

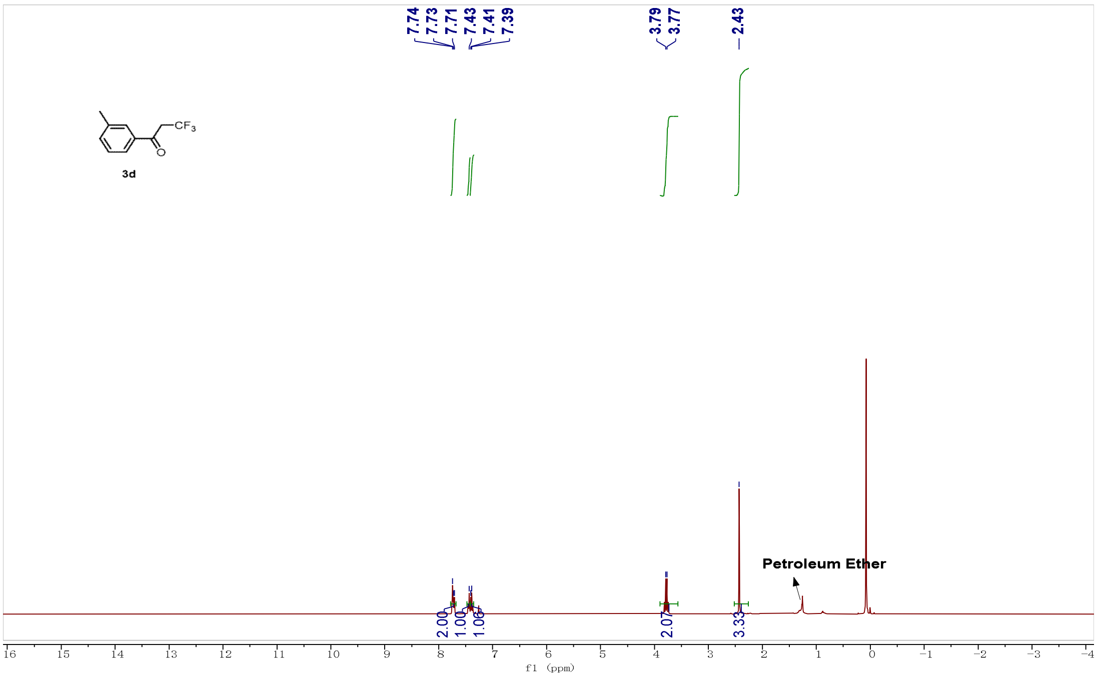

^{19}F NMR spectrum of 3,3,3-trifluoro-1-(m-tolyl)propan-1-one (**3d**) (376 MHz, Chloroform-*d*):

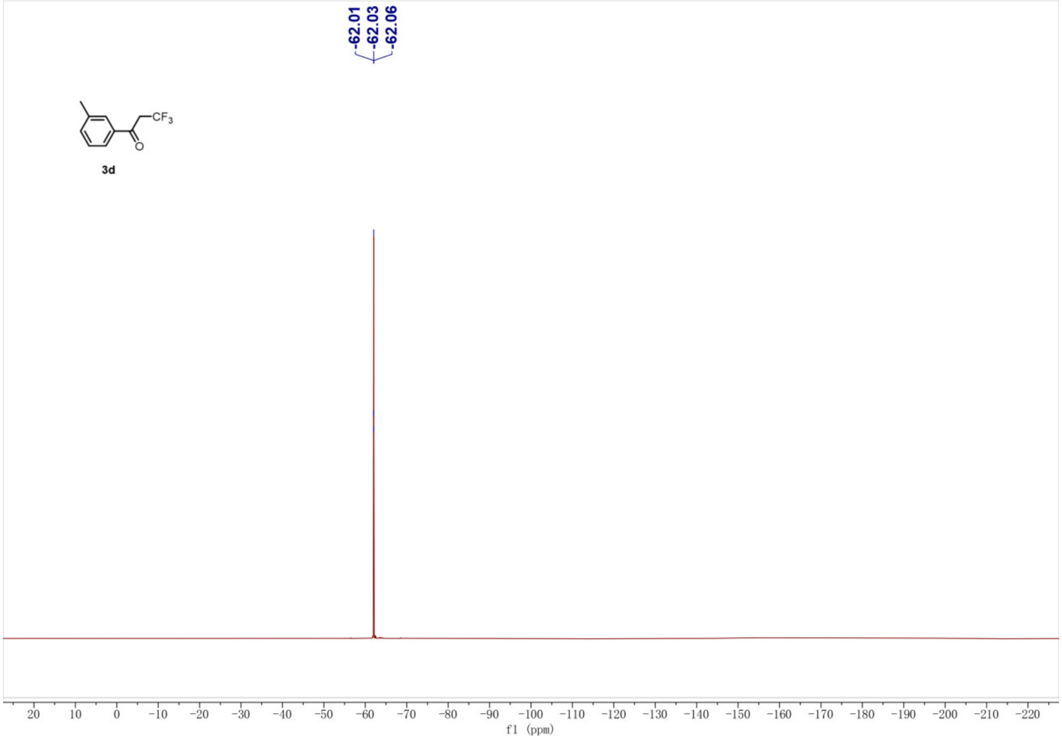

^{13}C NMR spectrum of 3,3,3-trifluoro-1-(m-tolyl)propan-1-one (**3d**) (101 MHz, Chloroform-*d*):

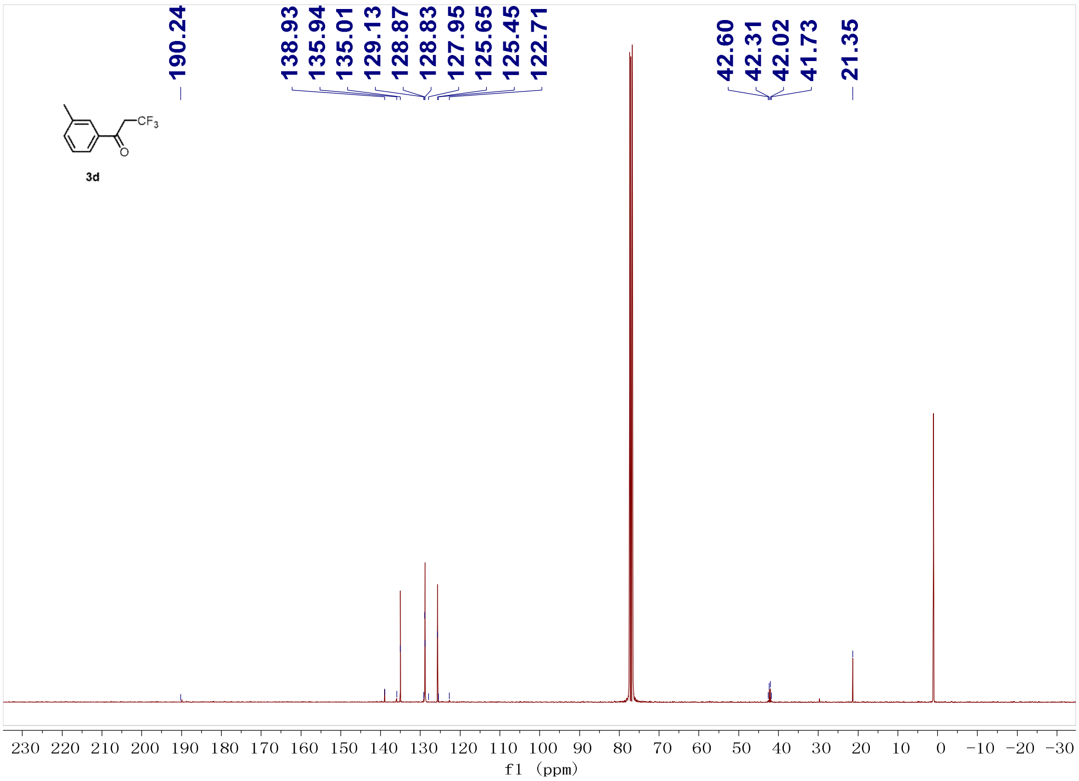

^{1}H NMR spectrum of 3,3,3-trifluoro-1-(4-methoxyphenyl)propan-1-one (**3e**) (400 MHz, Chloroform-*d*):

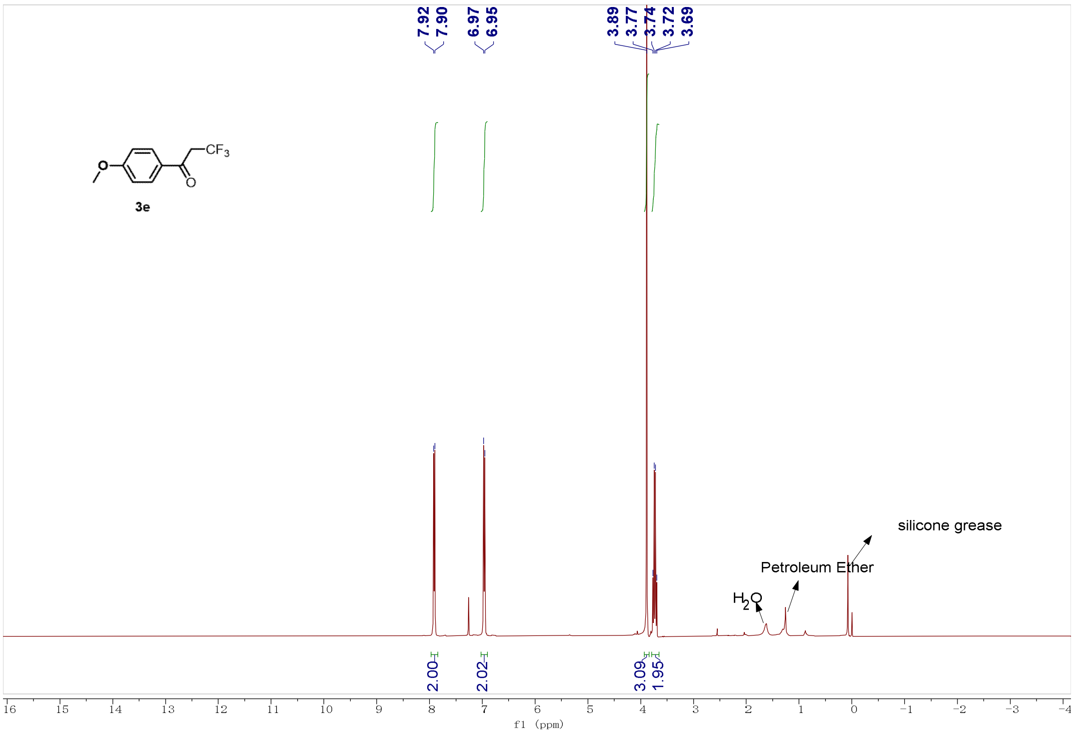

^{19}F NMR spectrum of 3,3,3-trifluoro-1-(4-methoxyphenyl)propan-1-one (**3e**) (376MHz, Chloroform-*d*):

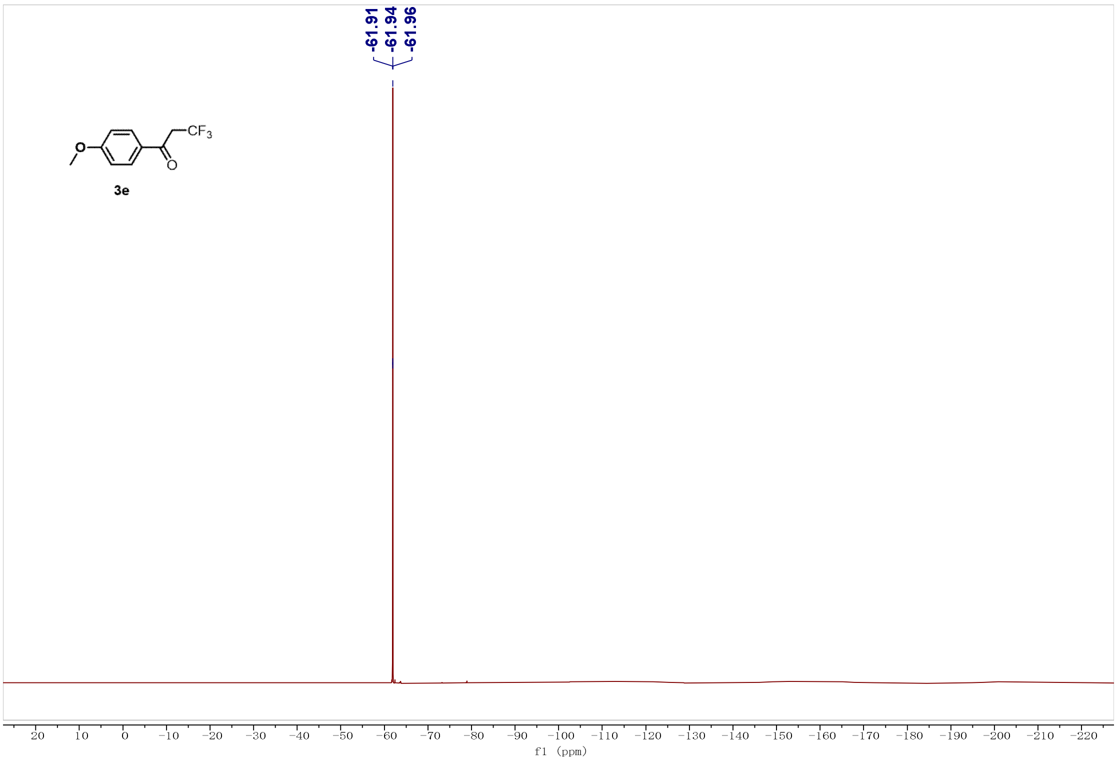

^{13}C NMR spectrum of 3,3,3-trifluoro-1-(4-methoxyphenyl)propan-1-one (**3e**) (101 MHz, Chloroform-*d*):

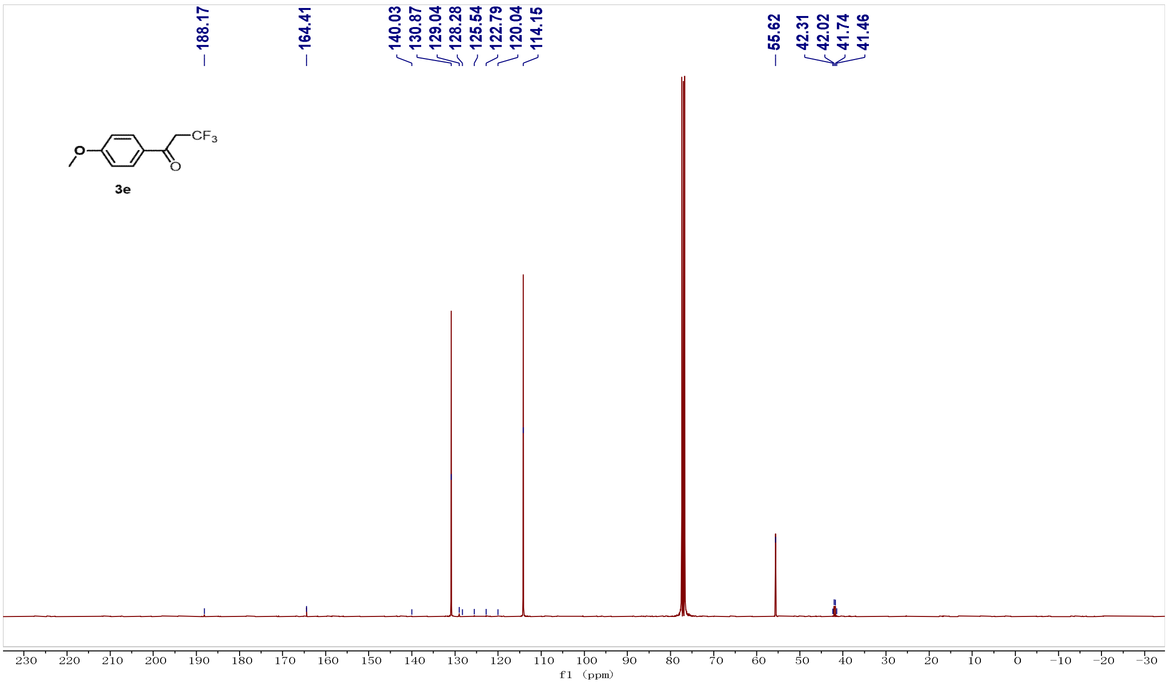

^{1}H NMR spectrum of 3,3,3-trifluoro-1-(3-methoxyphenyl)propan-1-one (**3f**) (400 MHz, Chloroform-*d*):

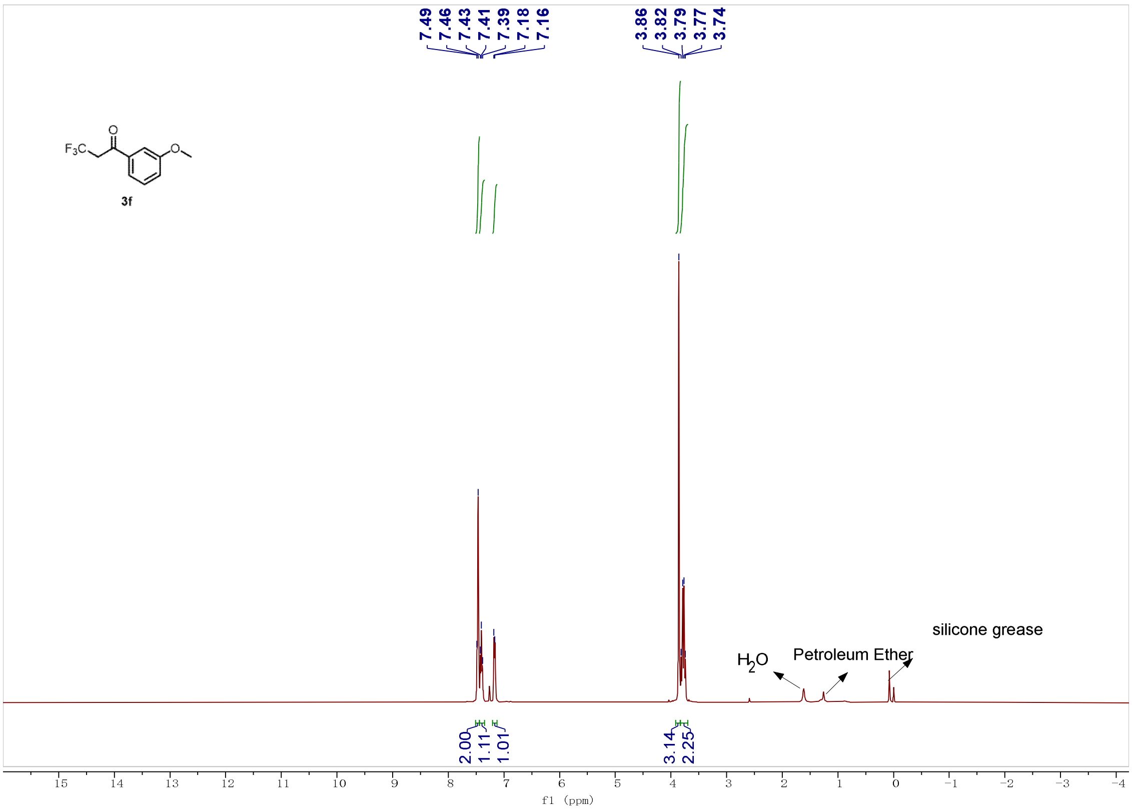

^{19}F NMR spectrum of 3,3,3-trifluoro-1-(3-methoxyphenyl)propan-1-one (**3f**) (376 MHz, Chloroform-*d*):

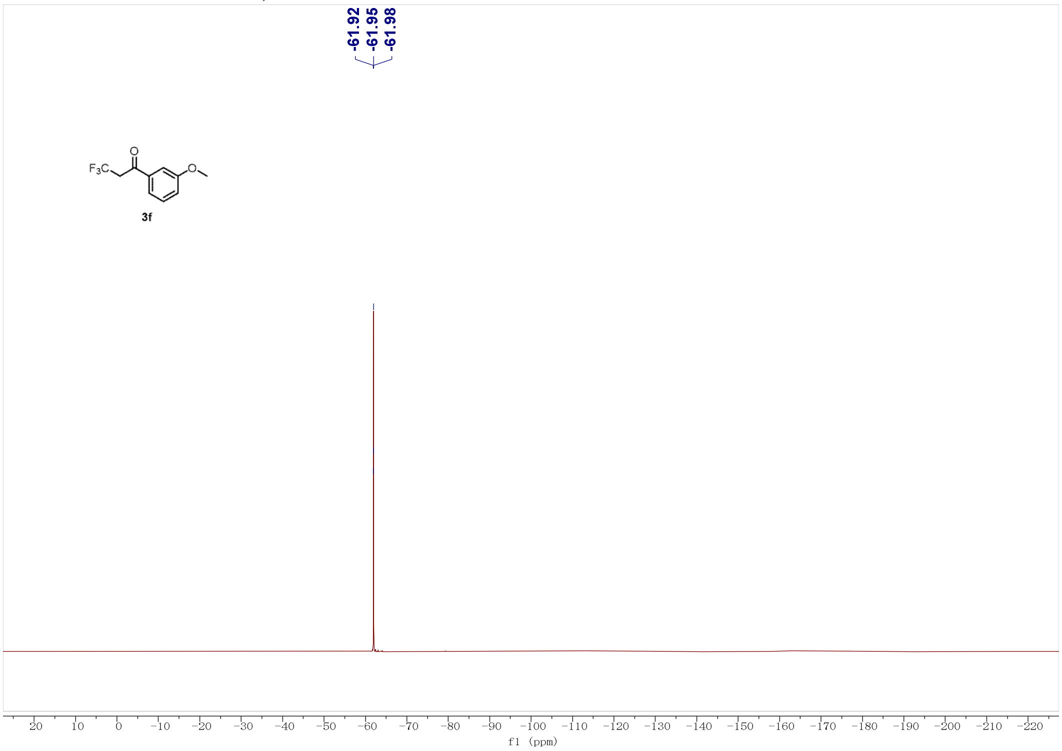

^{13}C NMR spectrum of 3,3,3-trifluoro-1-(3-methoxyphenyl)propan-1-one (**3f**) (101 MHz, Chloroform-*d*):

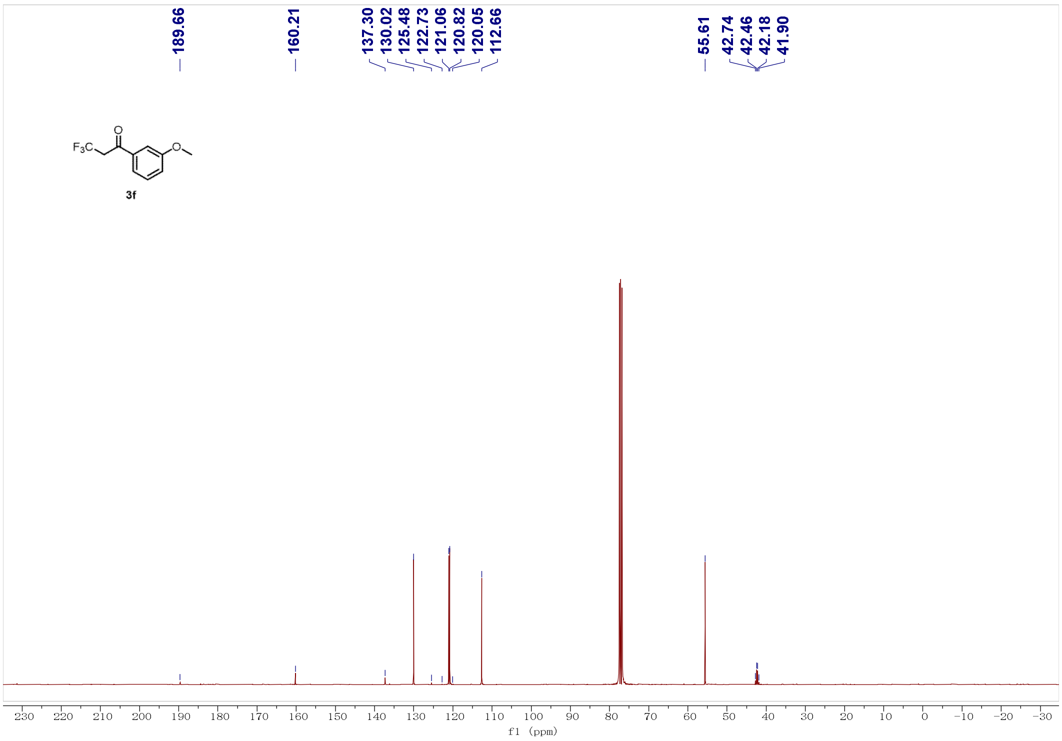

^{1}H NMR spectrum of 4-(3,3,3-trifluoropropanoyl)phenyl acetate (**3g**) (400 MHz, Chloroform-*d*):

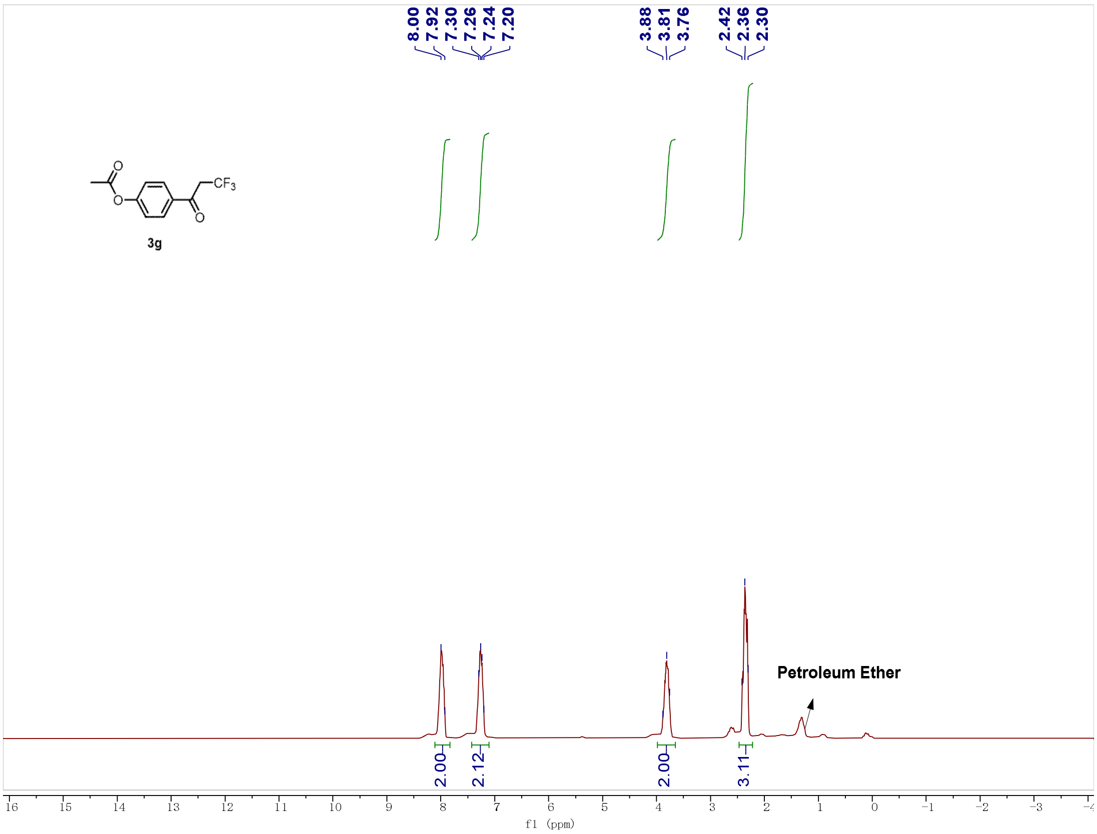

^{19}F NMR spectrum of 4-(3,3,3-trifluoropropanoyl)phenyl acetate (**3g**) (376 MHz, Chloroform-*d*):

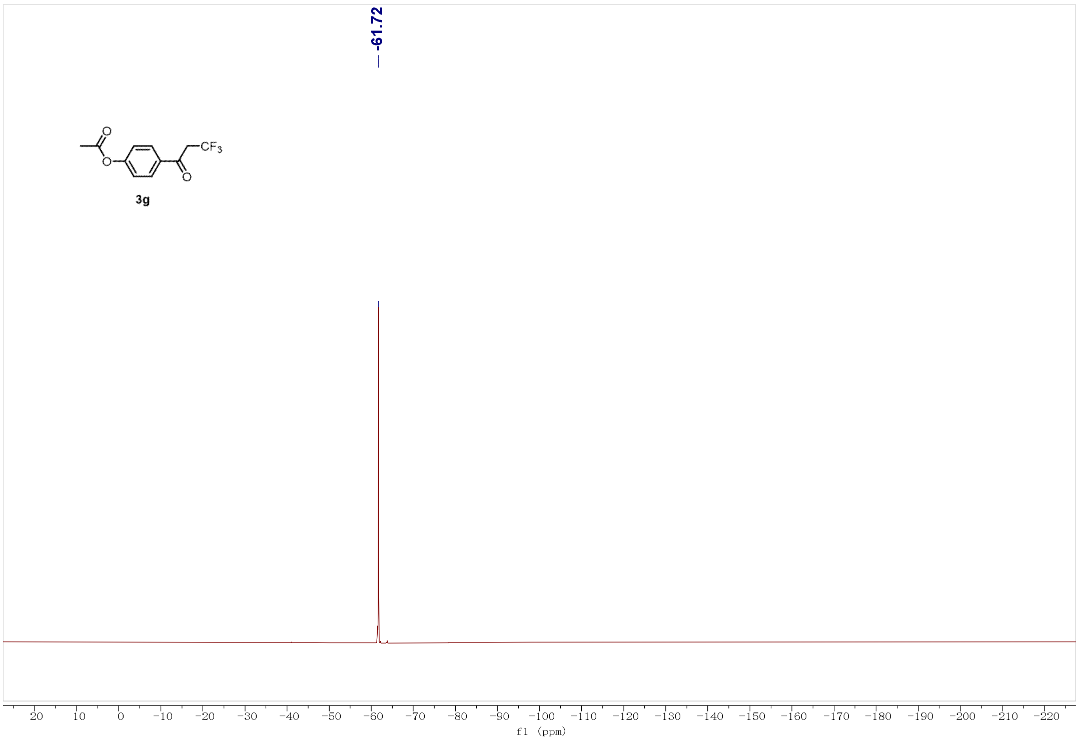

^{13}C NMR spectrum of 4-(3,3,3-trifluoropropanoyl)phenyl acetate (**3g**) (101 MHz, Chloroform-*d*):

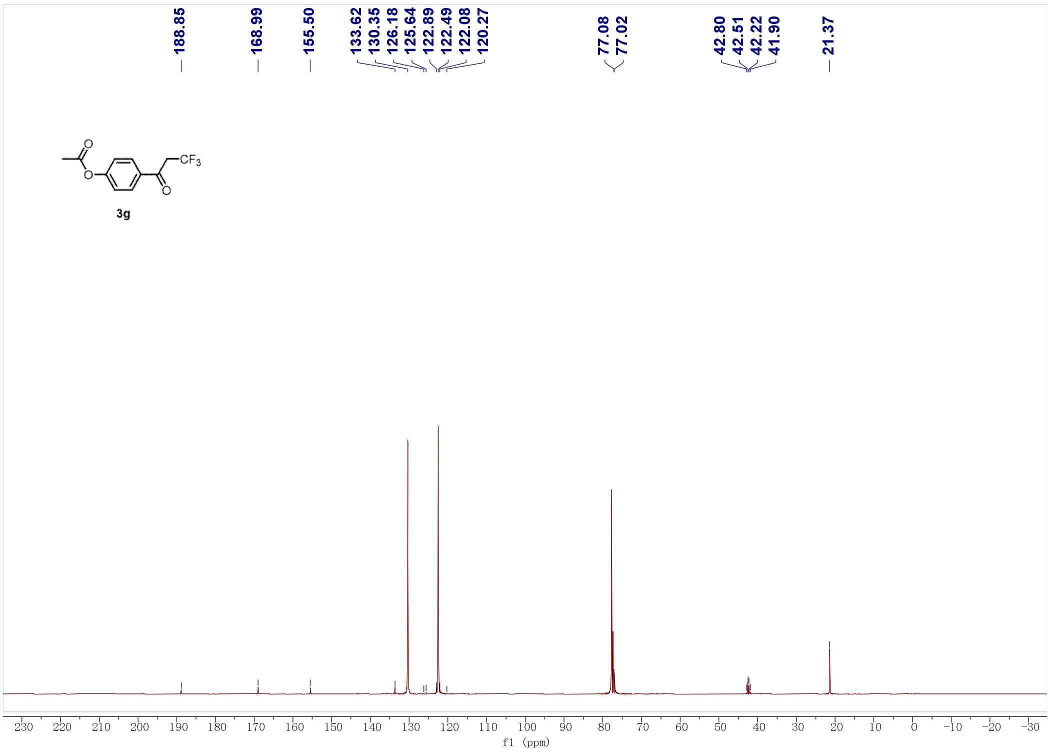

^{1}H NMR spectrum of 3,3,3-trifluoro-1-(4-fluorophenyl)propan-1-one (**3h**) (400 MHz, Chloroform-*d*):

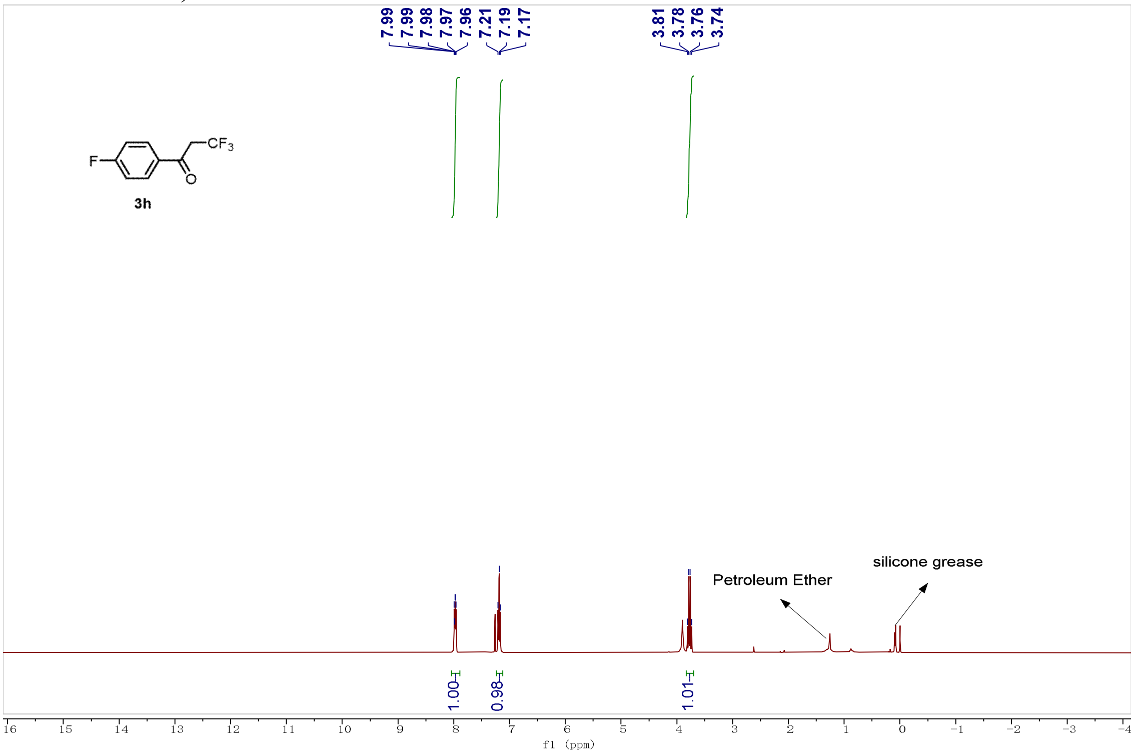

^{19}F NMR spectrum of 3,3,3-trifluoro-1-(4-fluorophenyl)propan-1-one (**3h**) (376 MHz, Chloroform-*d*):

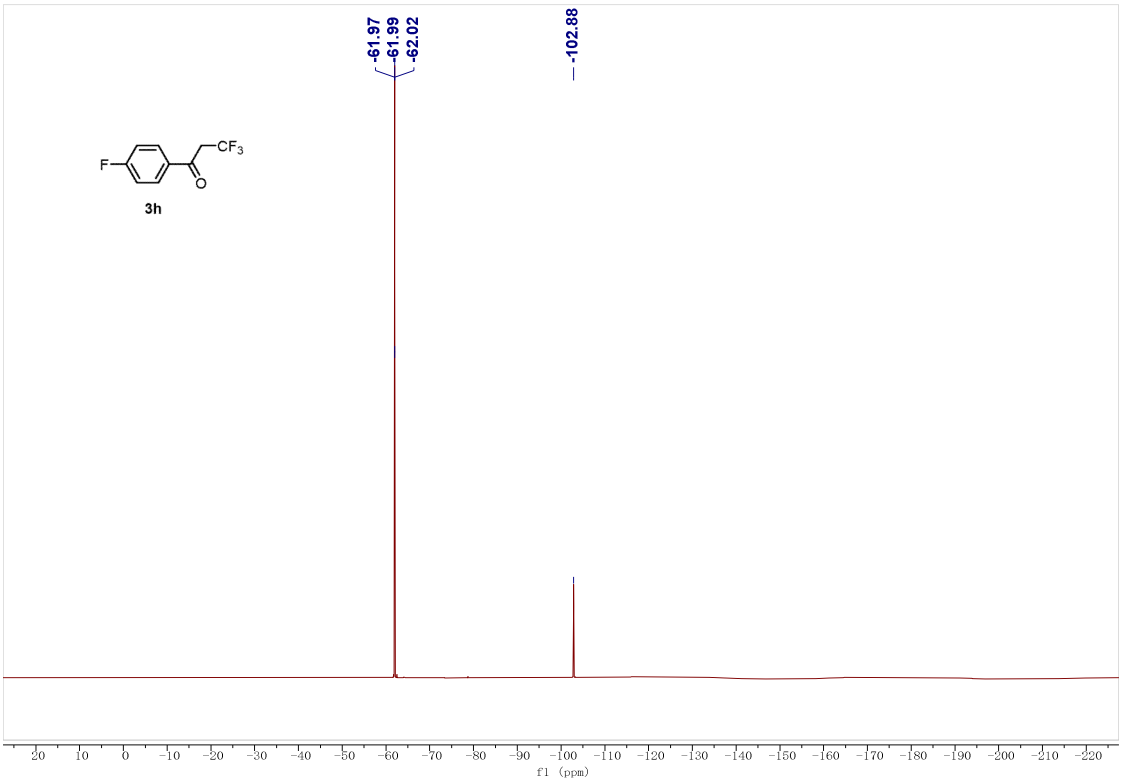

^{13}C NMR spectrum of 3,3,3-trifluoro-1-(4-fluorophenyl)propan-1-one (**3h**) (101 MHz, Chloroform-*d*):

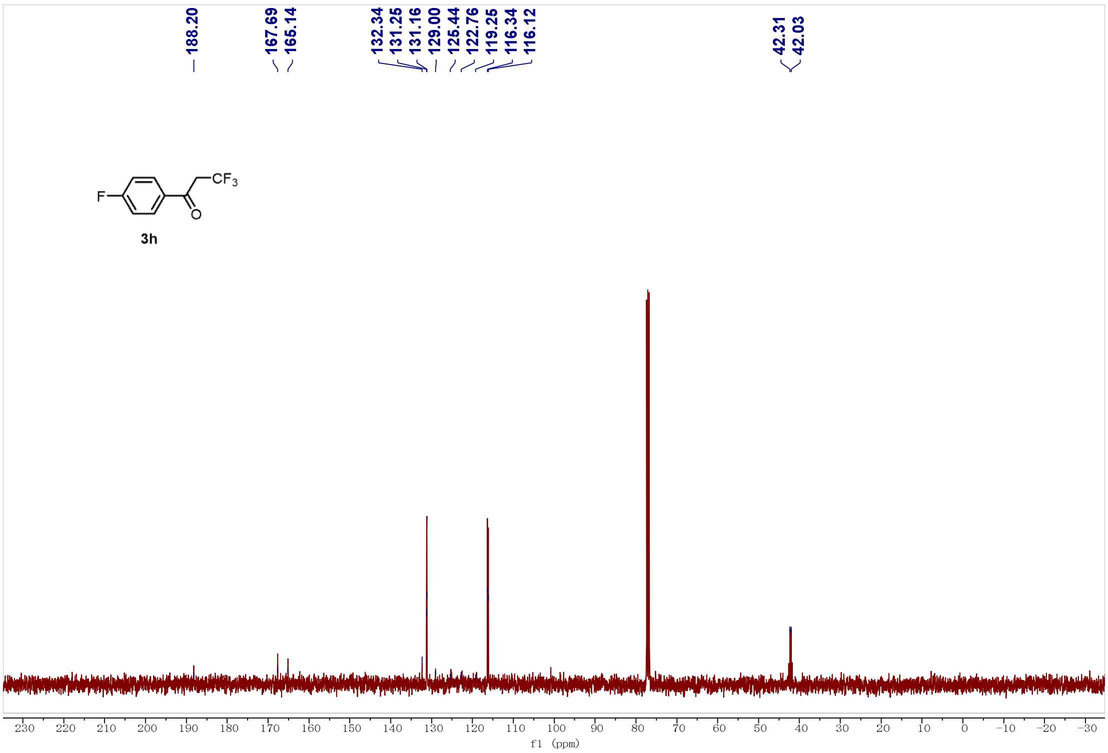

^{1}H NMR spectrum of 3,3,3-trifluoro-1-(3-fluorophenyl)propan-1-one (**3i**) (400 MHz, Chloroform-*d*):

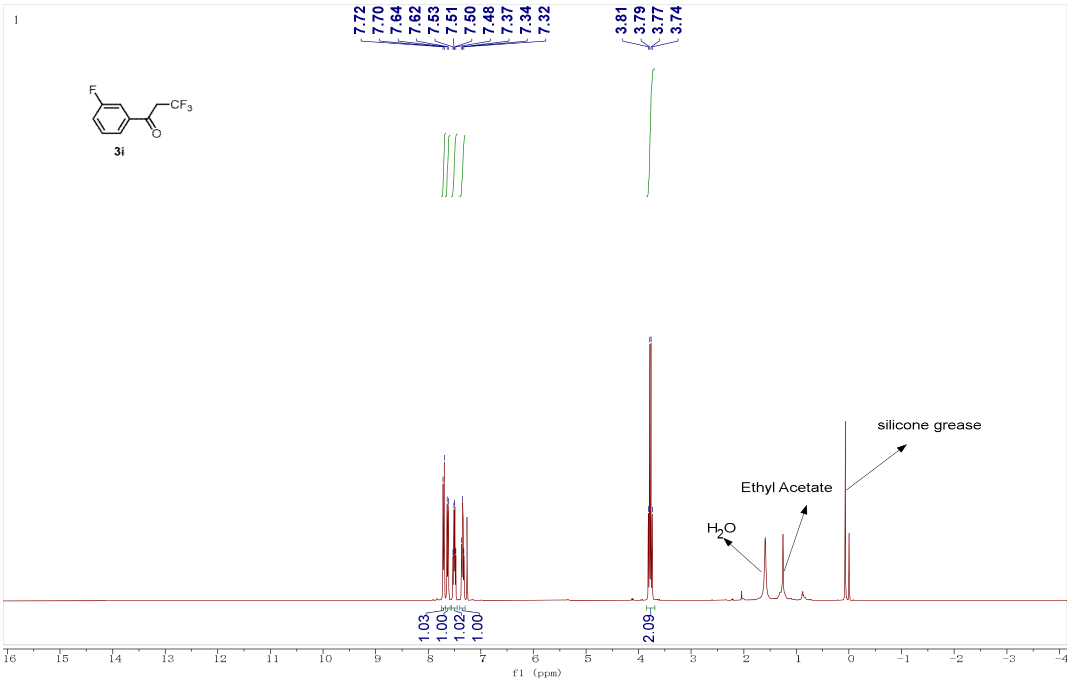

^{19}F NMR spectrum of 3,3,3-trifluoro-1-(3-fluorophenyl)propan-1-one (**3i**) (376 MHz, Chloroform-*d*):

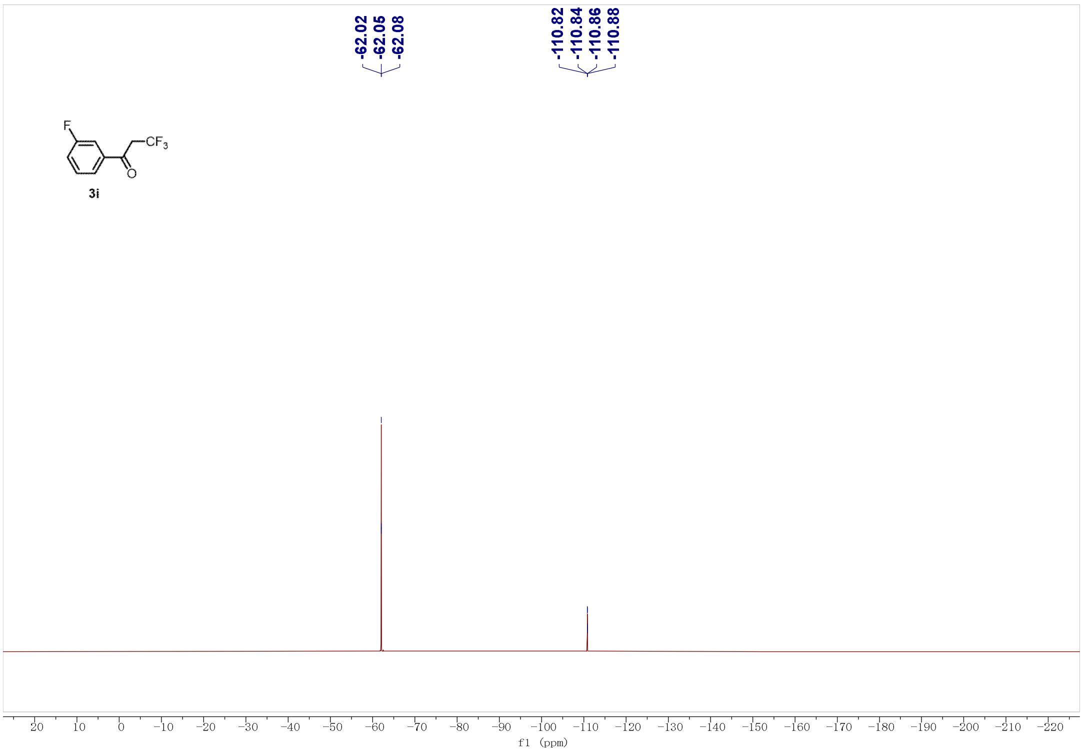

^{13}C NMR spectrum of 3,3,3-trifluoro-1-(3-fluorophenyl)propan-1-one (**3i**) (101 MHz, $CDCl_3$):

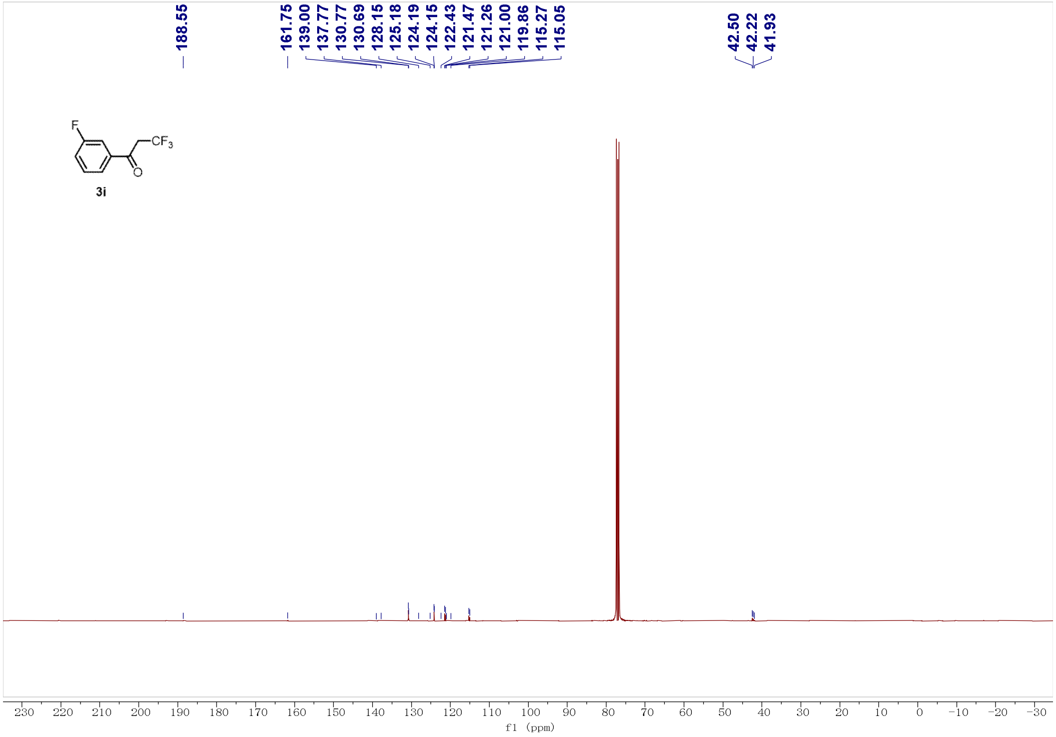

^{1}H NMR spectrum of 1-(4-chlorophenyl)-3,3,3-trifluoropropan-1-one (**3j**) (400 MHz, Chloroform-*d*):

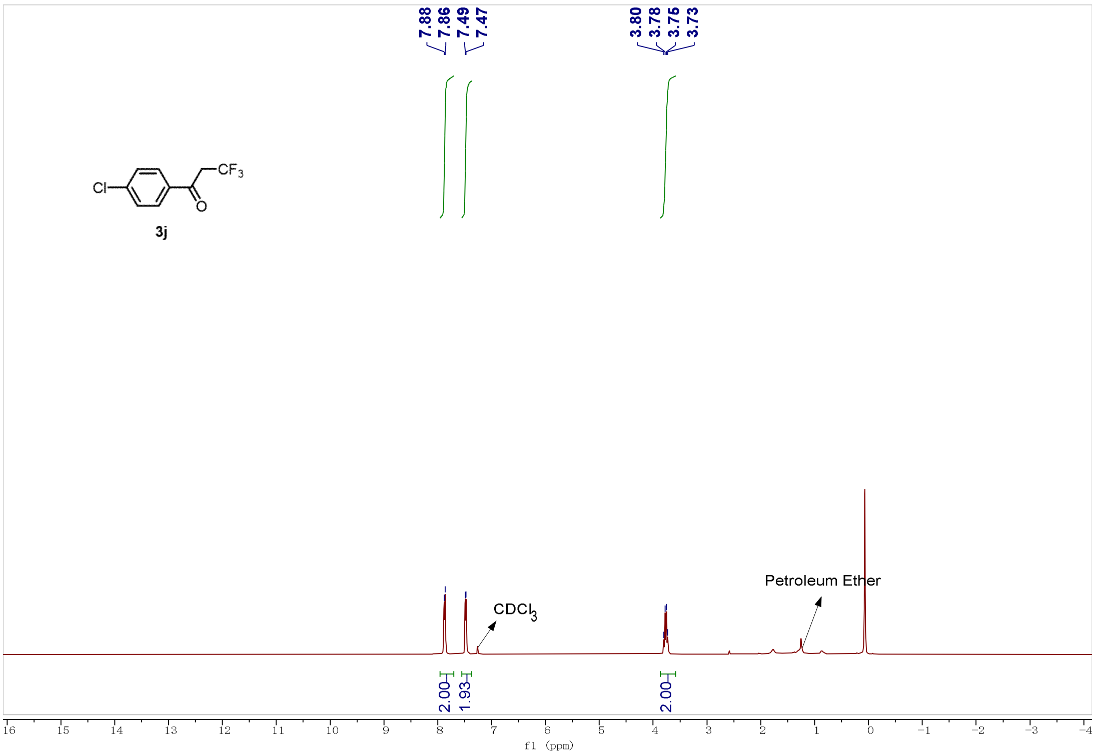

^{19}F NMR spectrum of 1-(4-chlorophenyl)-3,3,3-trifluoropropan-1-one (**3j**) (376 MHz, Chloroform-*d*):

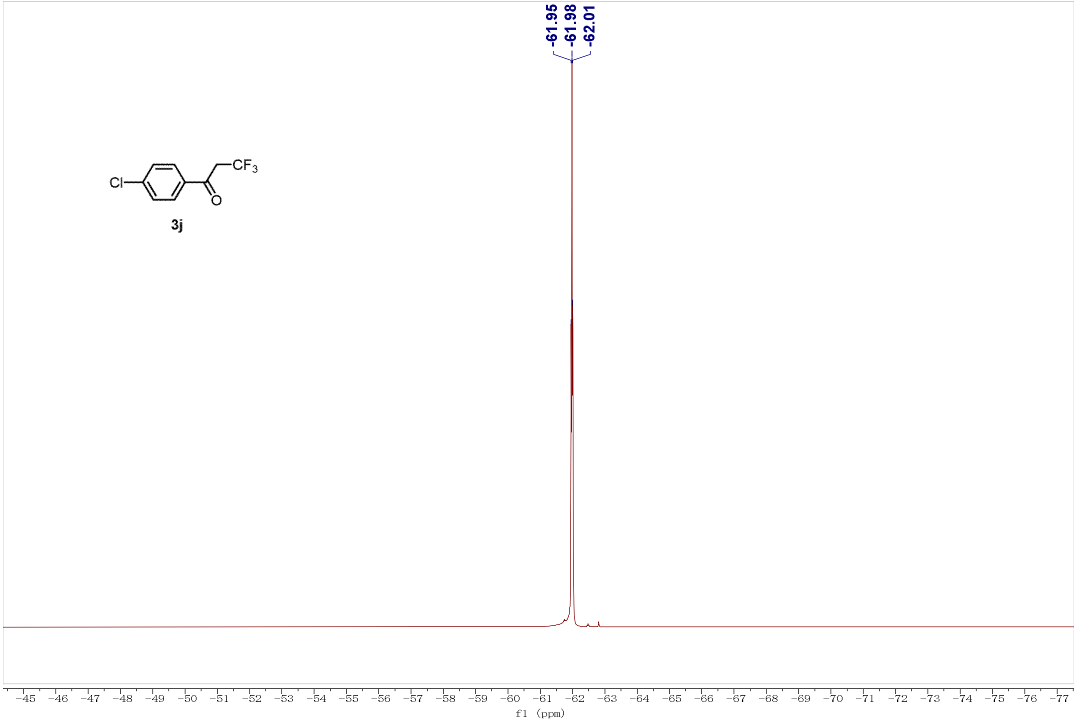

^{13}C NMR spectrum of 1-(4-chlorophenyl)-3,3,3-trifluoropropan-1-one (**3j**) (101 MHz, Chloroform-*d*):

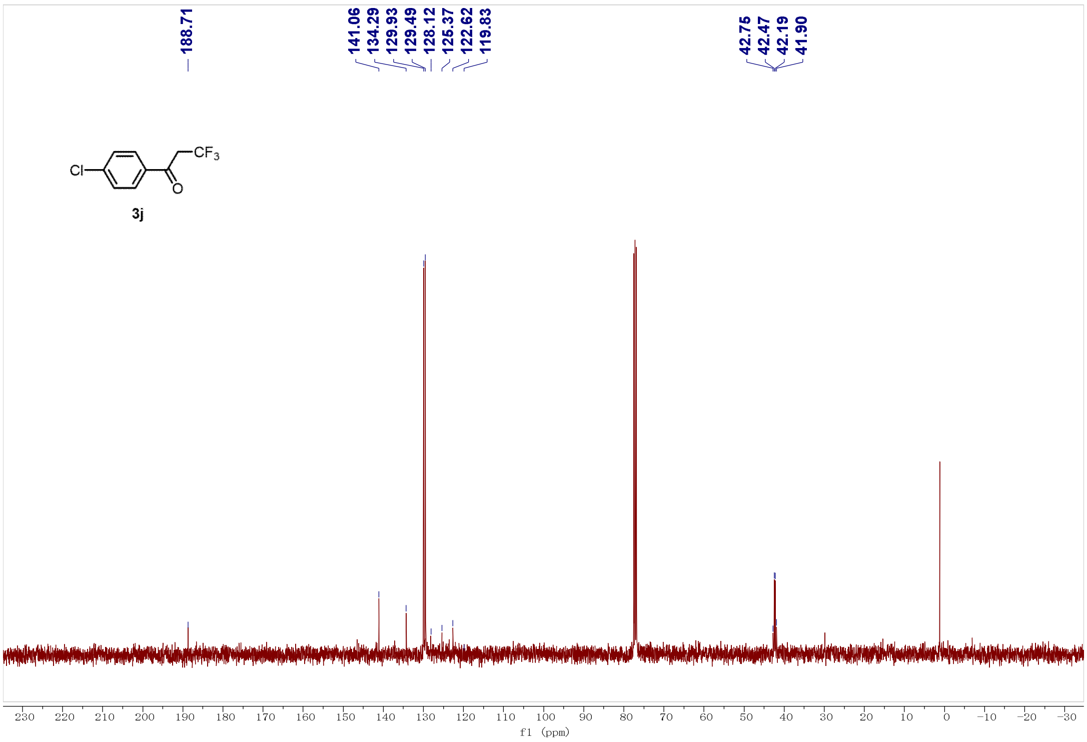

^1H NMR spectrum of 1-(4-bromophenyl)-3,3,3-trifluoropropan-1-one (**3k**) (400 MHz, Chloroform-*d*):

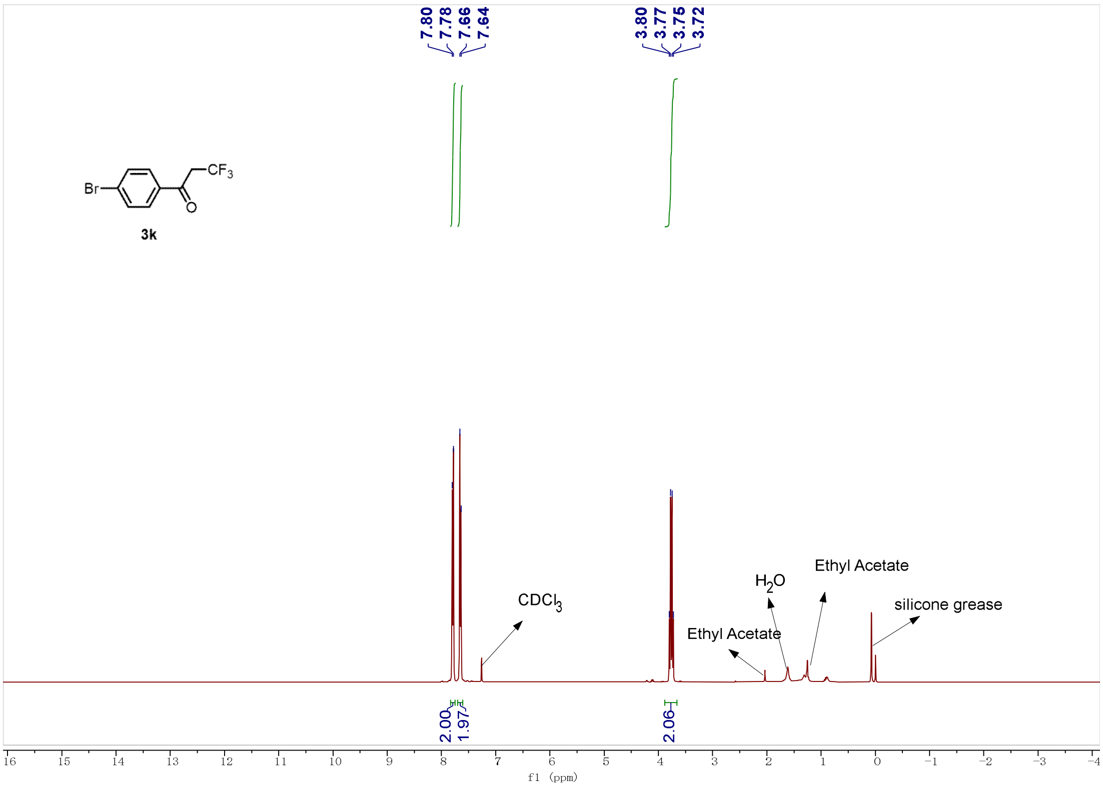

^{19}F NMR spectrum of 1-(4-bromophenyl)-3,3,3-trifluoropropan-1-one (**3k**) (376 MHz, Chloroform-*d*):

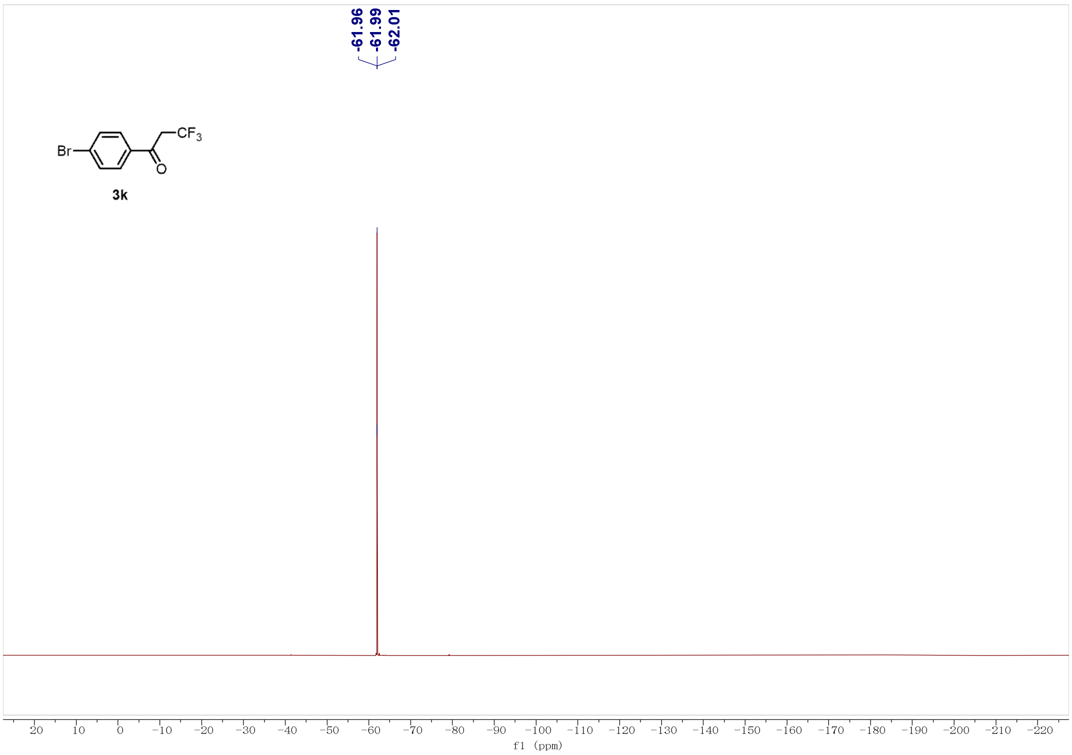

^{13}C NMR spectrum of 1-(4-bromophenyl)-3,3,3-trifluoropropan-1-one (**3k**) (101 MHz, $CDCl_3$):

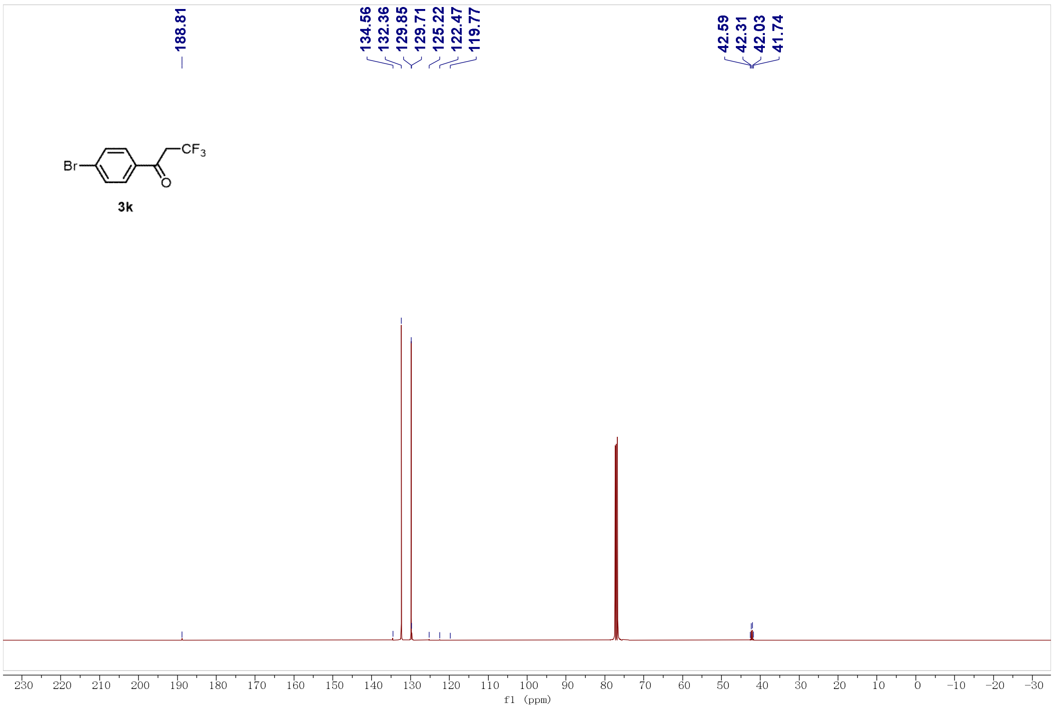

^{1}H NMR spectrum of 3,3,3-trifluoro-1-(4-(trifluoromethyl)phenyl)propan-1-one (**3l**) (400 MHz, Chloroform-*d*):

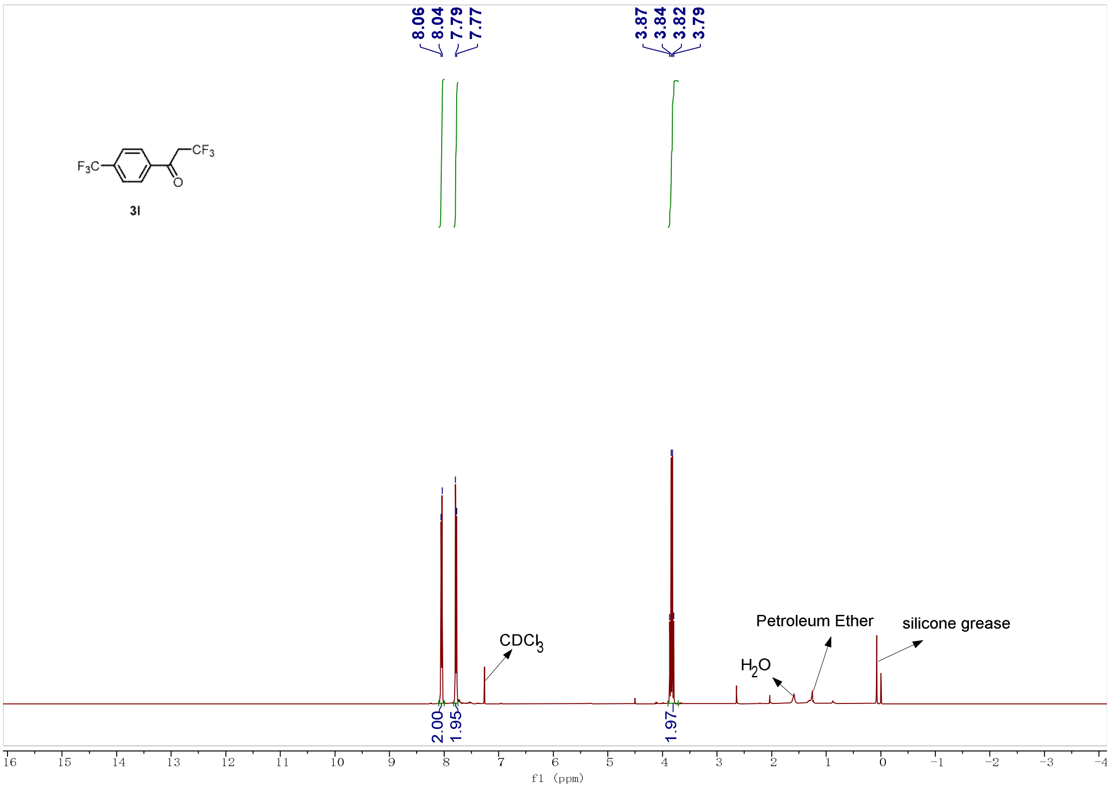

^{19}F NMR spectrum of 3,3,3-trifluoro-1-(4-(trifluoromethyl)phenyl)propan-1-one (**3l**) (376MHz,Chloroform-*d*):

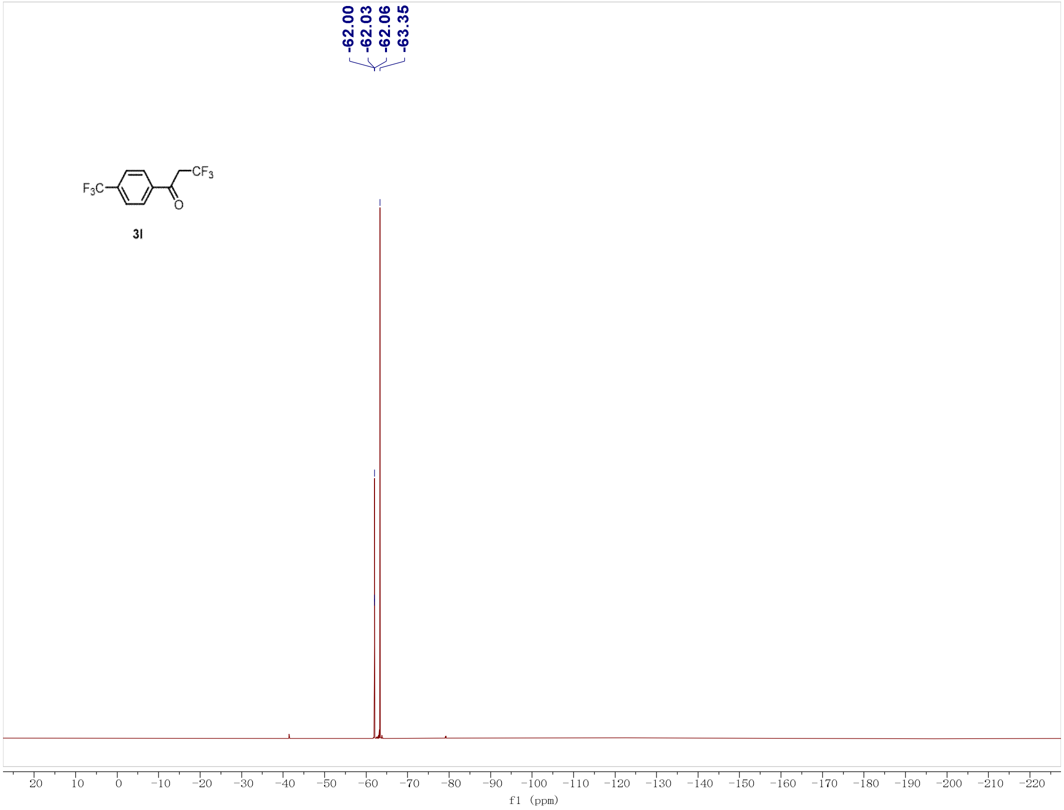

^{13}C NMR spectrum of 3,3,3-trifluoro-1-(4-(trifluoromethyl)phenyl)propan-1-one (**3l**) (101 MHz, Chloroform-*d*):

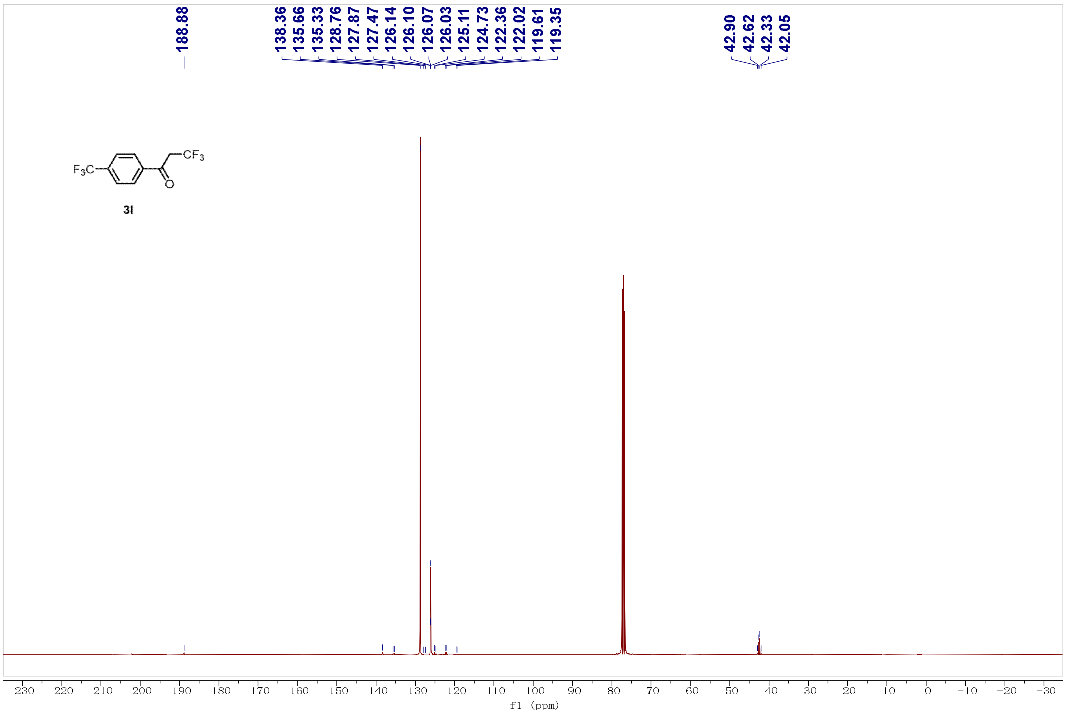

^{1}H NMR spectrum of 3,3,3-trifluoro-1-(4-isocyanophenyl)propan-1-one (**3m**) (400 MHz, Chloroform-*d*):

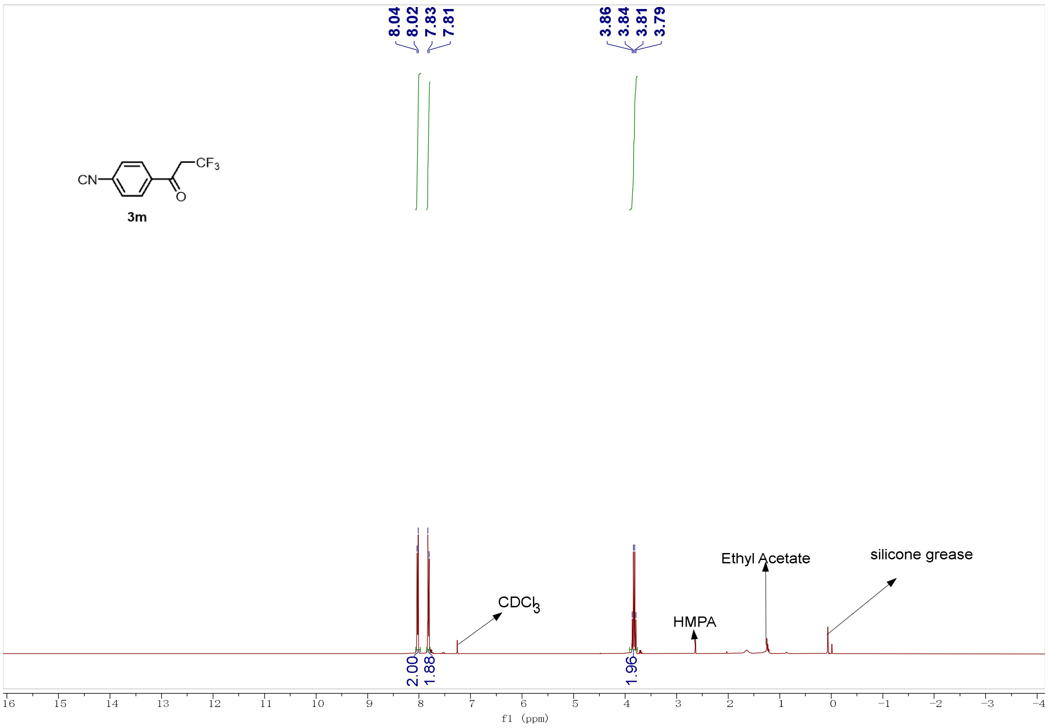

^{19}F NMR spectrum of 3,3,3-trifluoro-1-(4-isocyanophenyl)propan-1-one (**3m**) (376 MHz, Chloroform-*d*):

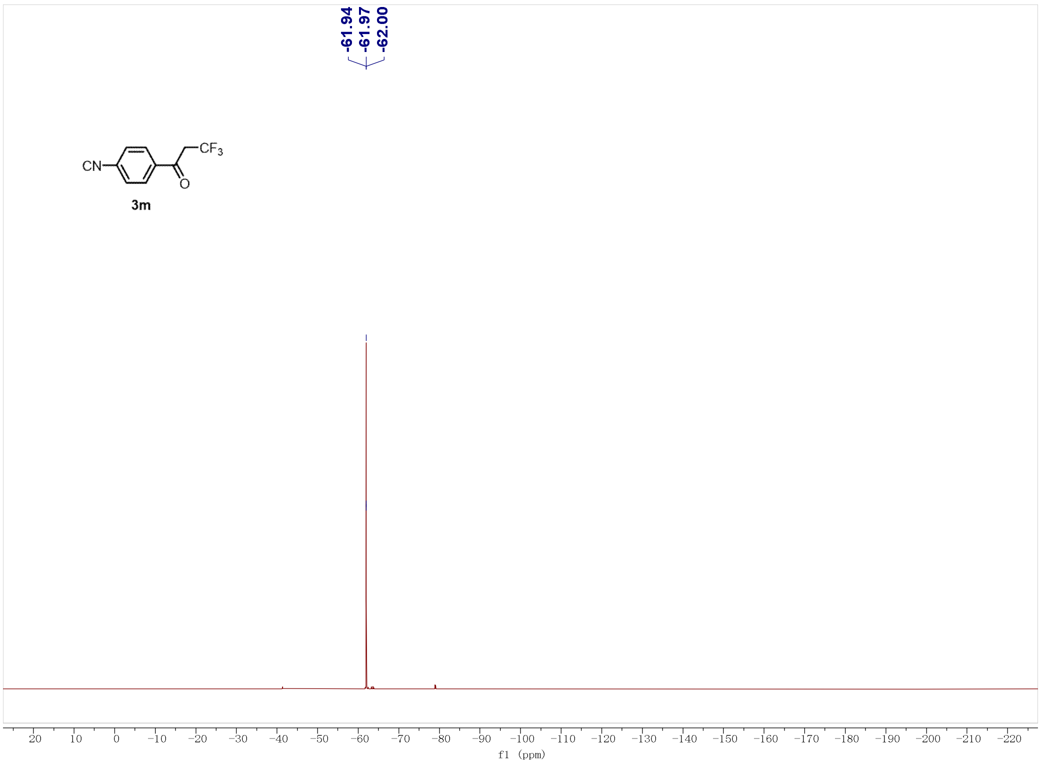

^{13}C NMR spectrum of 3,3,3-trifluoro-1-(4-isocyanophenyl)propan-1-one (**3m**) (101 MHz, Chloroform-*d*):

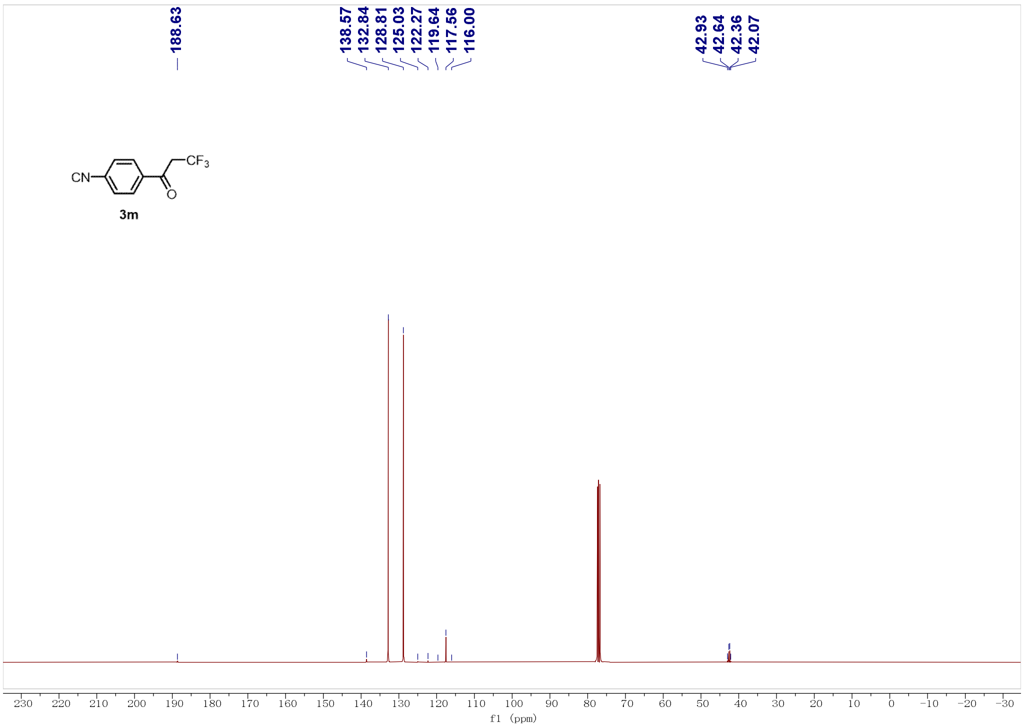

^1H NMR spectrum of 3,3,3-trifluoro-1-(4-nitrophenyl)propan-1-one (**3n**) (400 MHz, Chloroform-*d*):

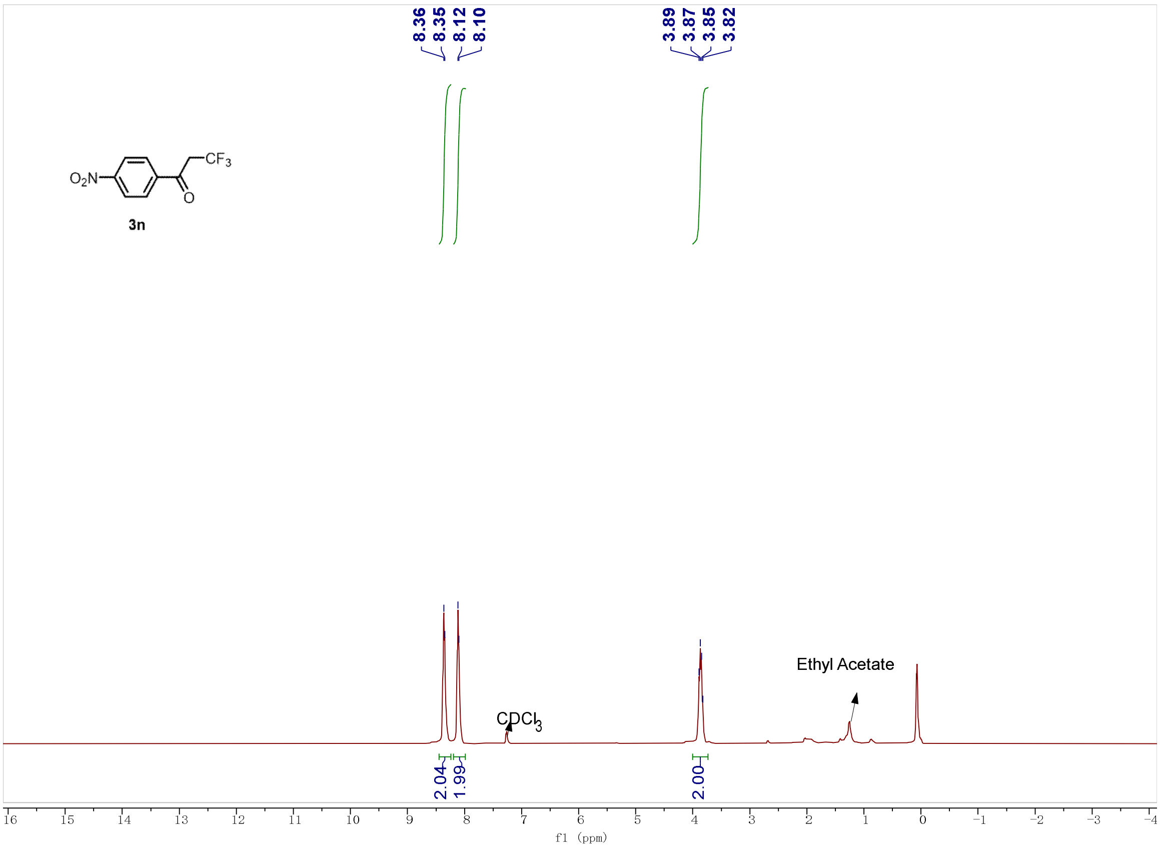

^{19}F NMR spectrum of 3,3,3-trifluoro-1-(4-nitrophenyl)propan-1-one (**3n**) (376 MHz, Chloroform-*d*):

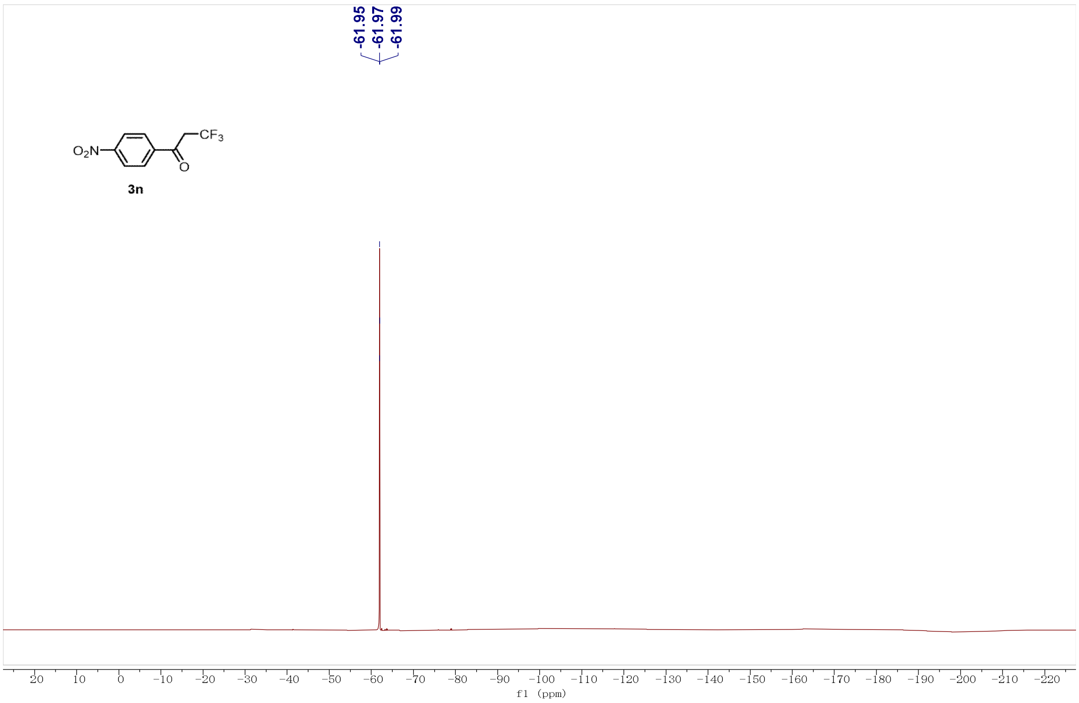

^{13}C NMR spectrum of 3,3,3-trifluoro-1-(4-nitrophenyl)propan-1-one (**3n**) (101 MHz, Chloroform-*d*):

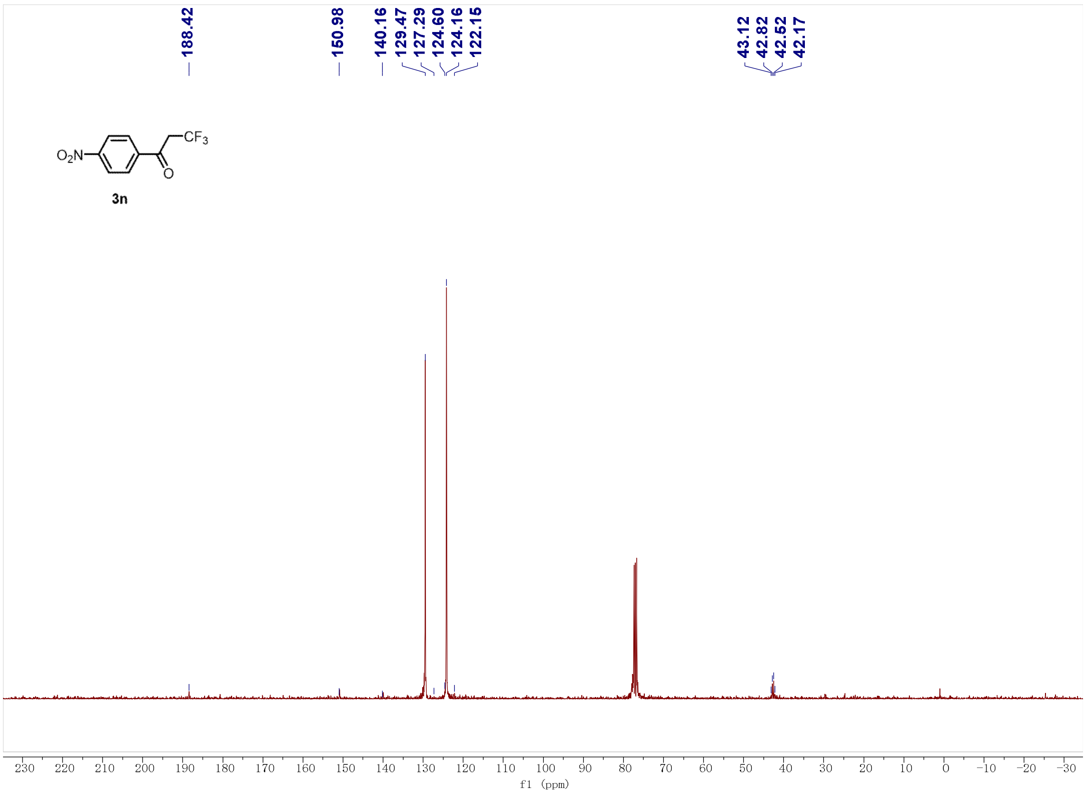

^{1}H NMR spectrum of methyl 4-(3,3,3-trifluoropropanoyl)benzoate (**3o**) (400 MHz, Chloroform-*d*):

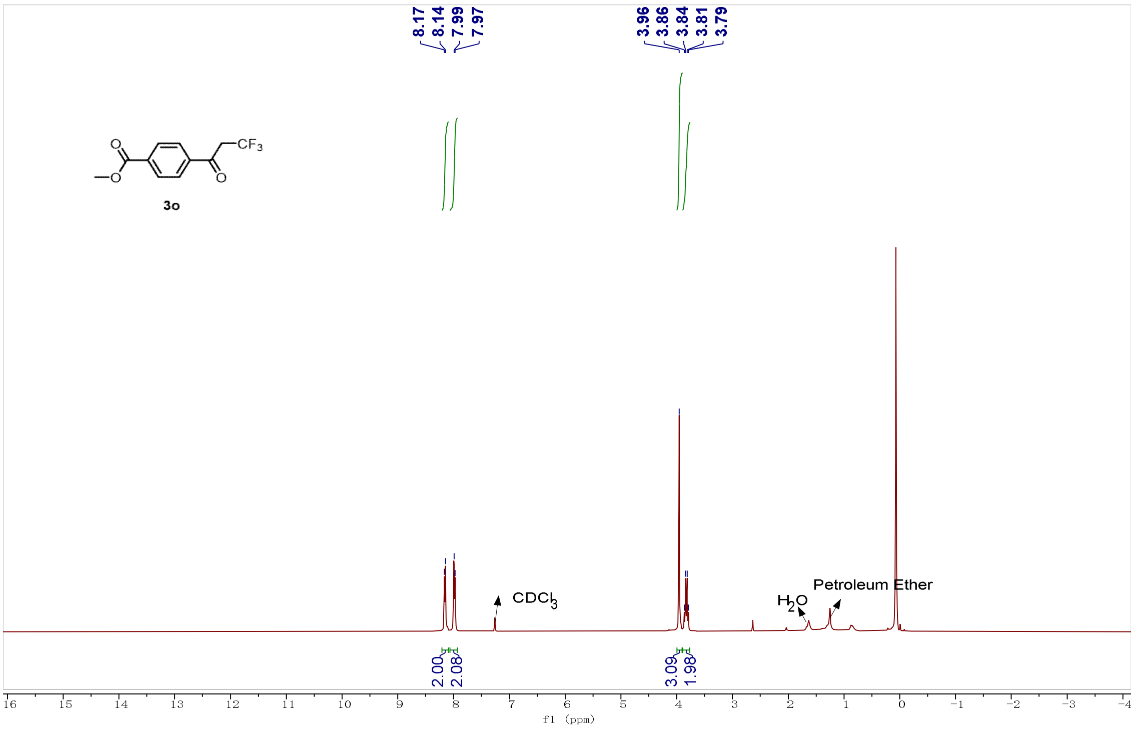

^{19}F NMR spectrum of methyl 4-(3,3,3-trifluoropropanoyl)benzoate (**3o**) (376 MHz, Chloroform-*d*):

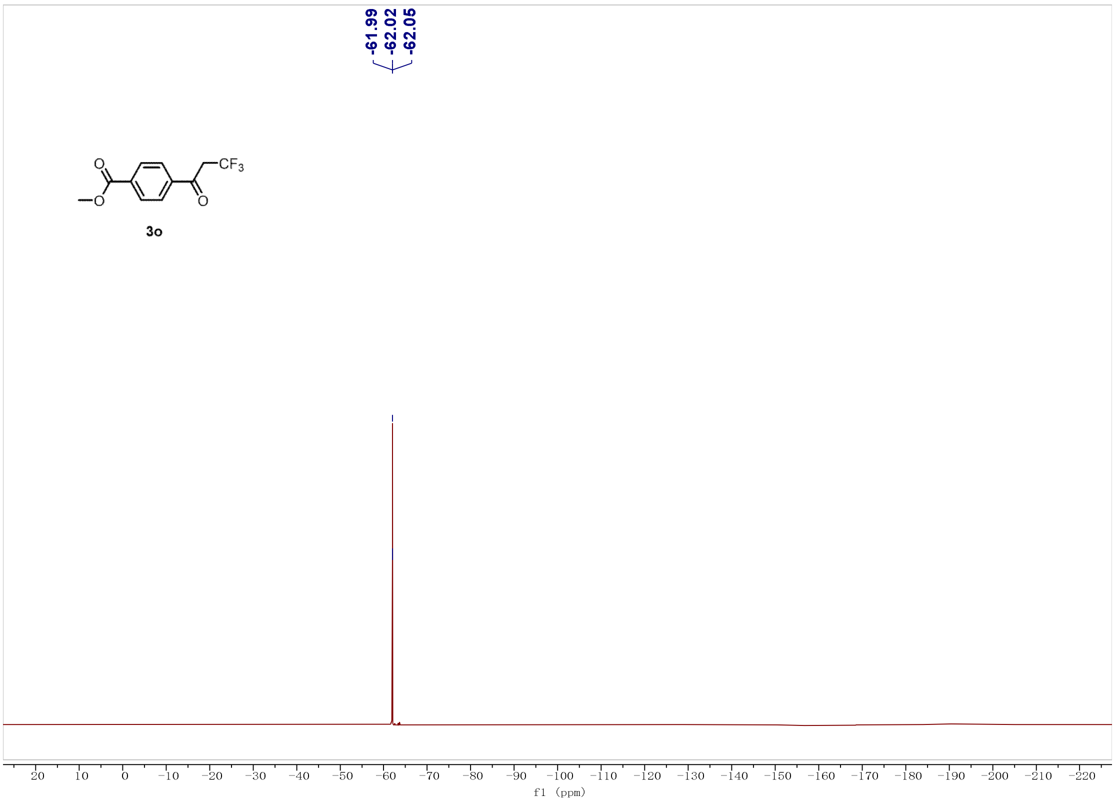

^{13}C NMR spectrum of methyl 4-(3,3,3-trifluoropropanoyl)benzoate (**3o**) (101 MHz, Chloroform-*d*):

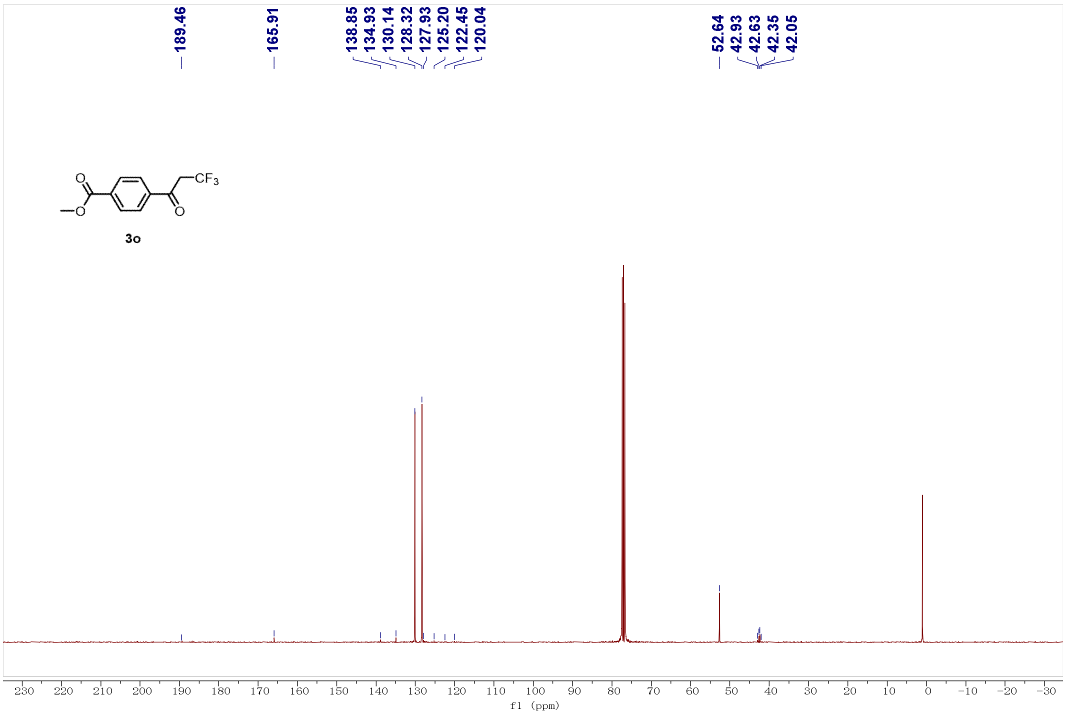

^{1}HNMR spectrum of 3,3,3-trifluoro-1-(naphthalen-2-yl)propan-1-one (**3p**) (400 MHz, Chloroform-*d*):

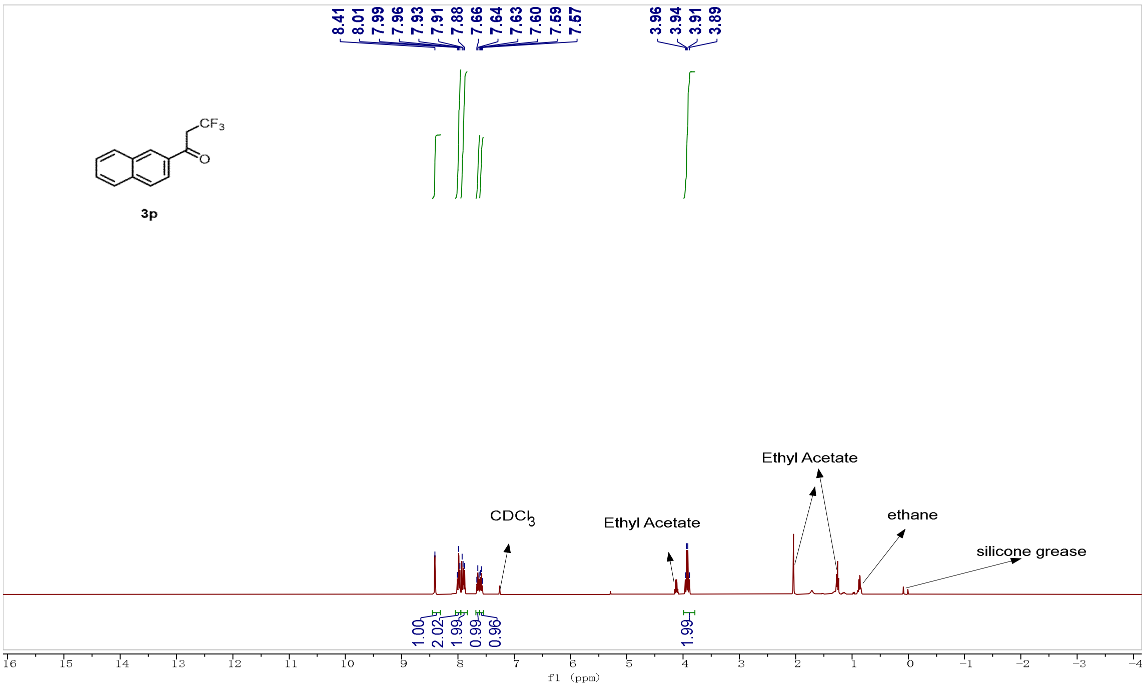

^{19}F NMR spectrum of 3,3,3-trifluoro-1-(naphthalen-2-yl)propan-1-one (**3p**) (376 MHz, Chloroform-*d*):

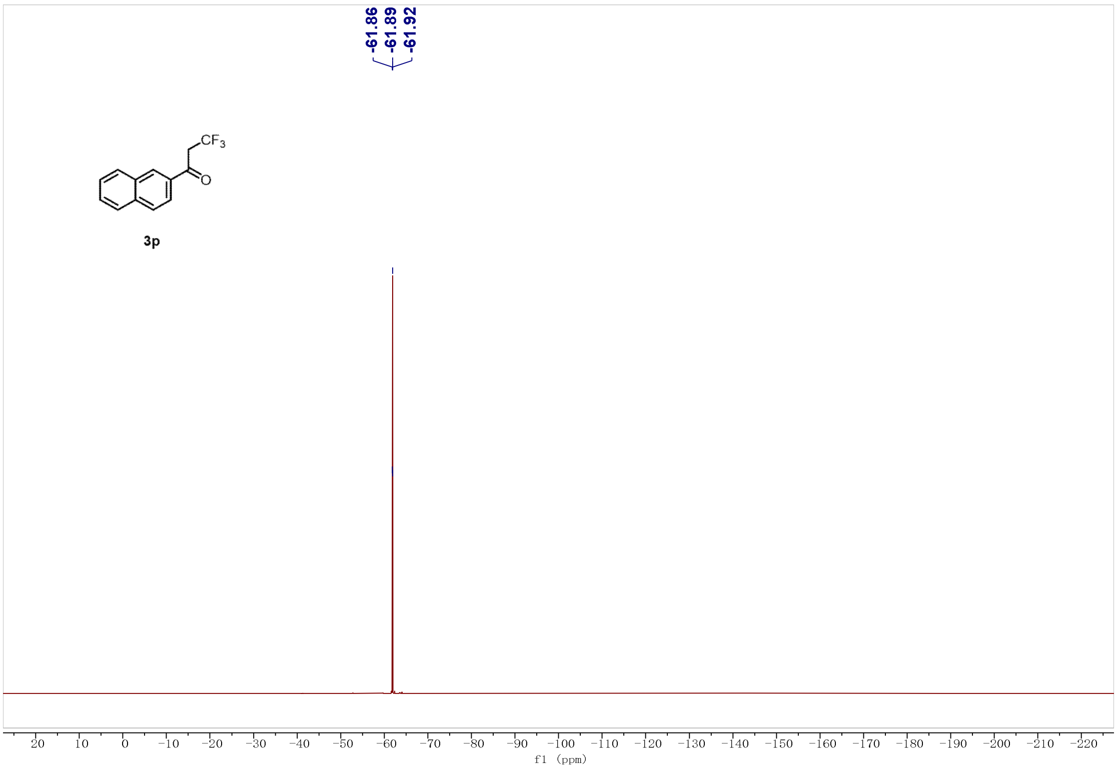

^{13}C NMR spectrum of 3,3,3-trifluoro-1-(naphthalen-2-yl)propan-1-one (**3p**) (101 MHz, Chloroform-*d*):

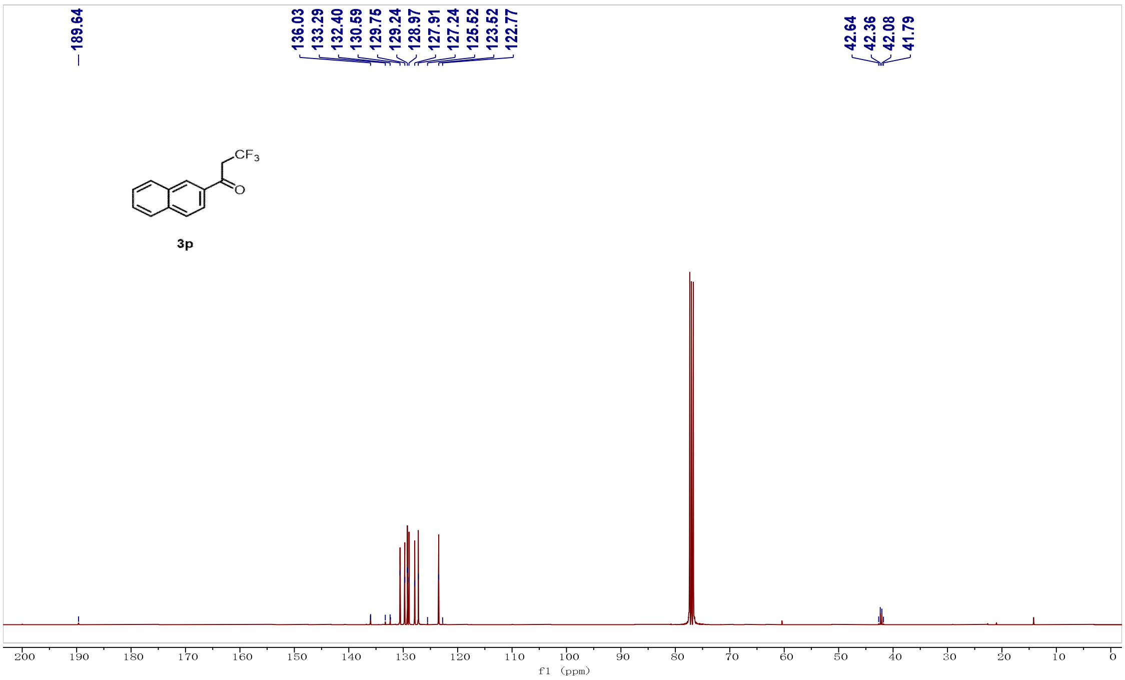

^{1}H NMR spectrum of 2-(trifluoromethyl)-3,4-dihydronaphthalen-1(2H)-one (**3q**) (400 MHz, Chloroform-*d*):

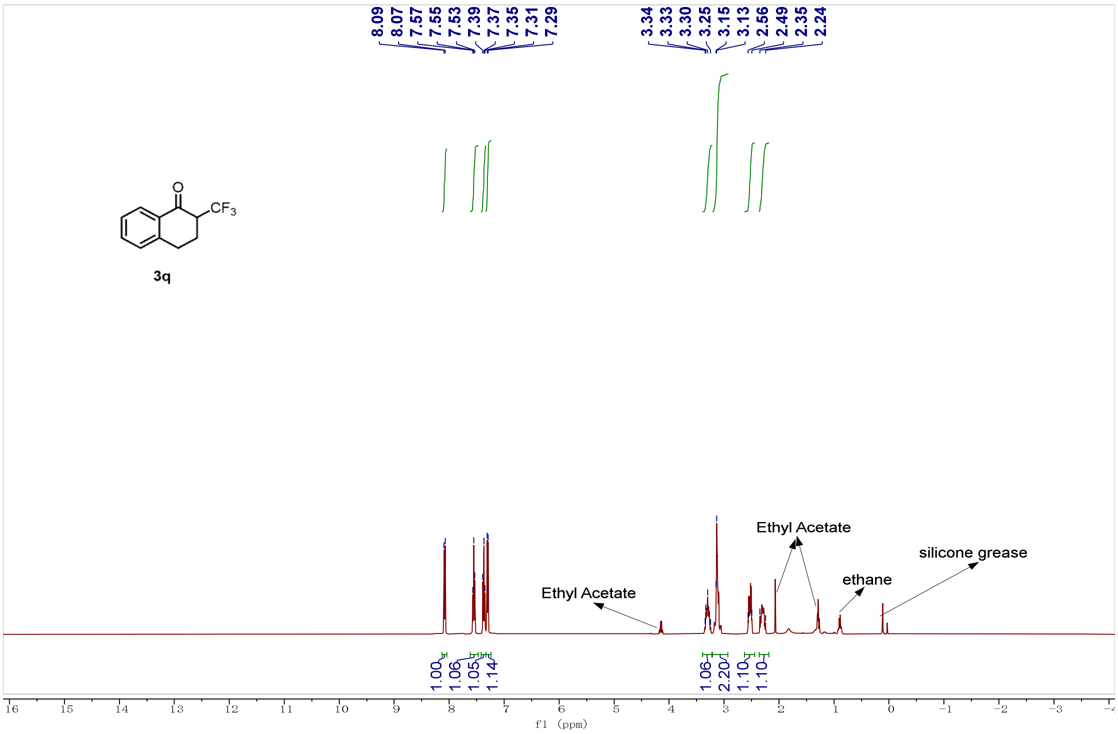

^{19}F NMR spectrum of 2-(trifluoromethyl)-3,4-dihydronaphthalen-1(2H)-one (**3q**) (376 MHz, Chloroform-*d*):

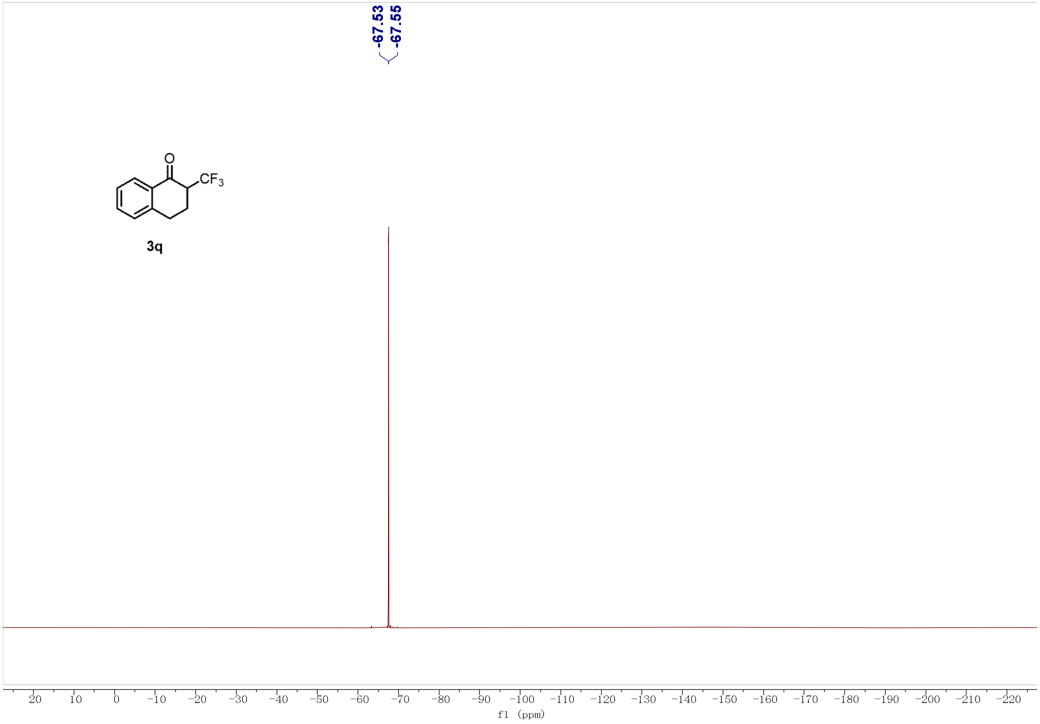

^{13}C NMR spectrum of 2-(trifluoromethyl)-3,4-dihydronaphthalen-1(2H)-one (**3q**) (101 MHz, Chloroform-*d*):

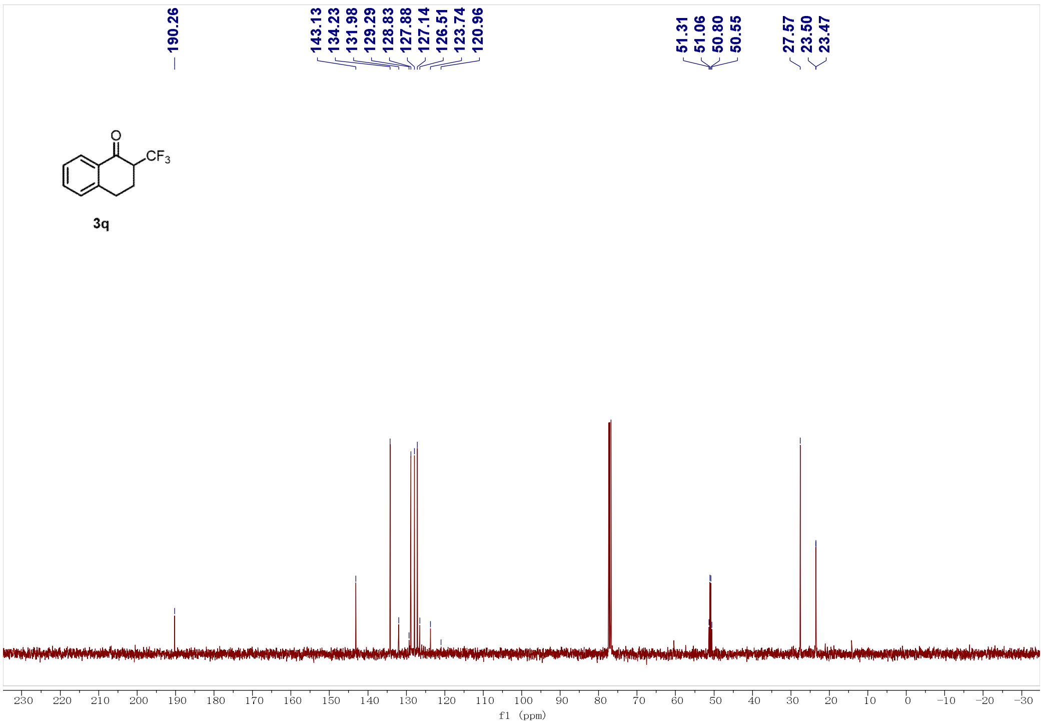

^1H NMR spectrum of 2-(trifluoromethyl)-2,3-dihydro-1H-inden-1-one (**3r**) (400 MHz, Chloroform-*d*):

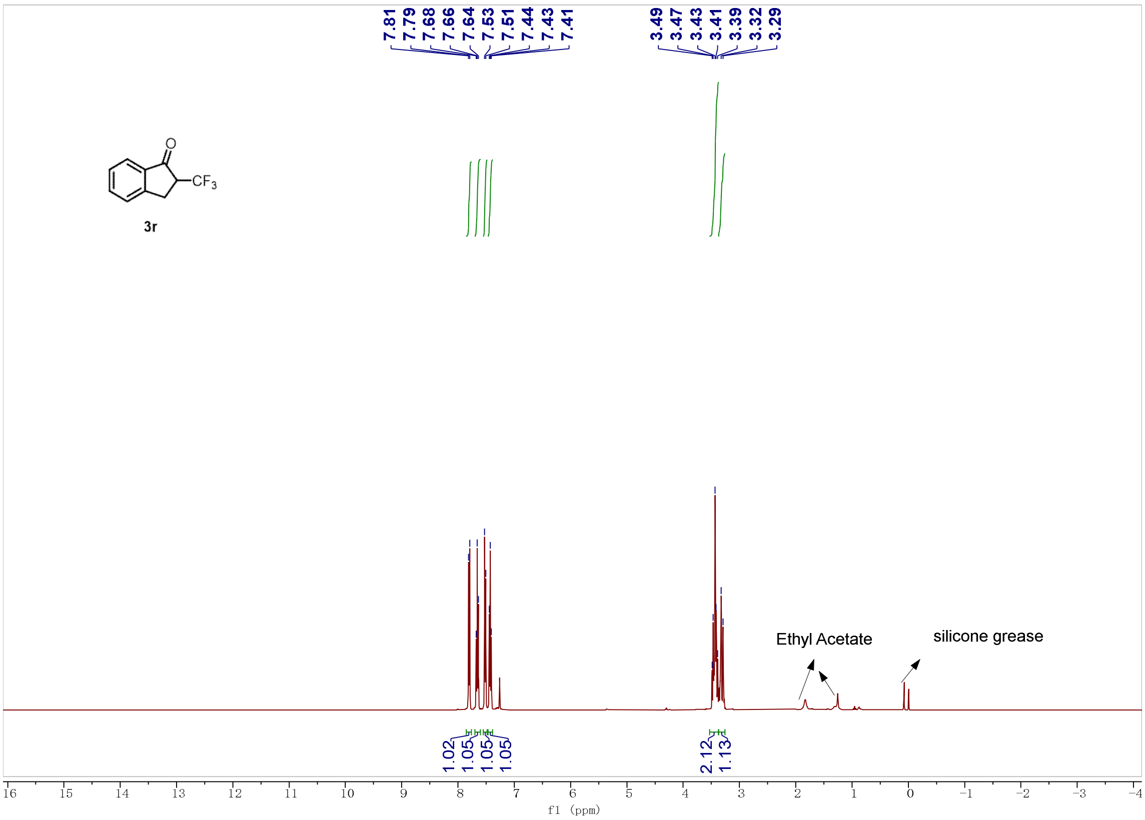

^{19}F NMR spectrum of 2-(trifluoromethyl)-2,3-dihydro-1H-inden-1-one (**3r**) (376 MHz, Chloroform-*d*):

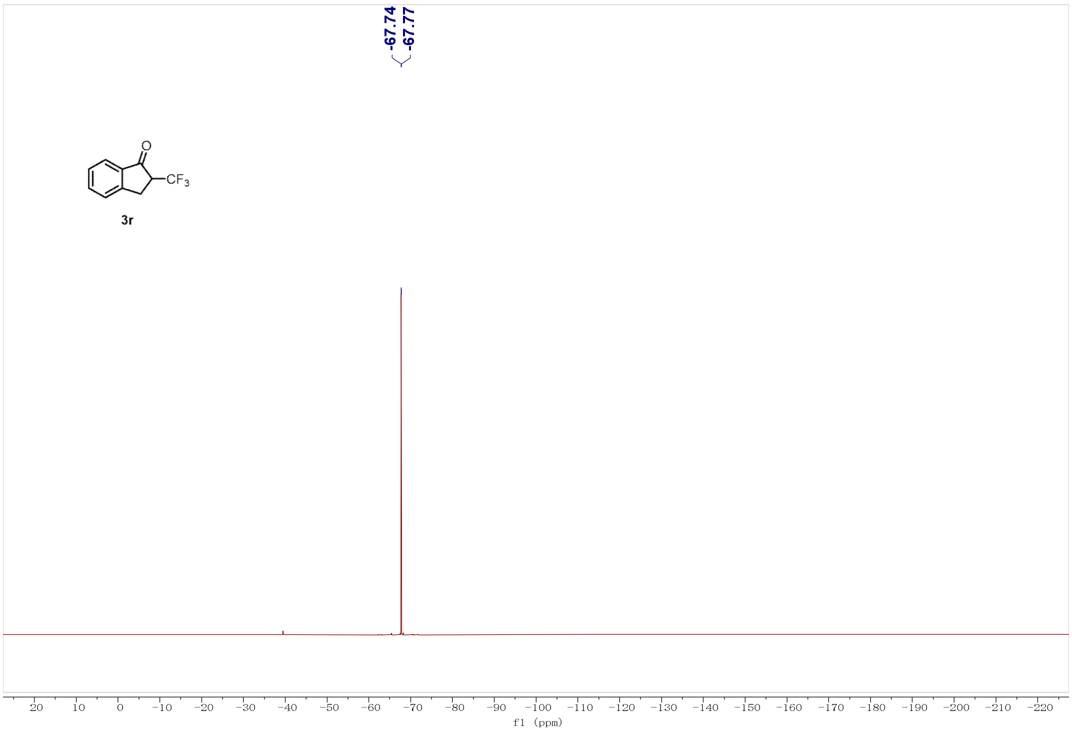

^{13}C NMR spectrum of 2-(trifluoromethyl)-2,3-dihydro-1H-inden-1-one (**3r**) (101 MHz, Chloroform-*d*):

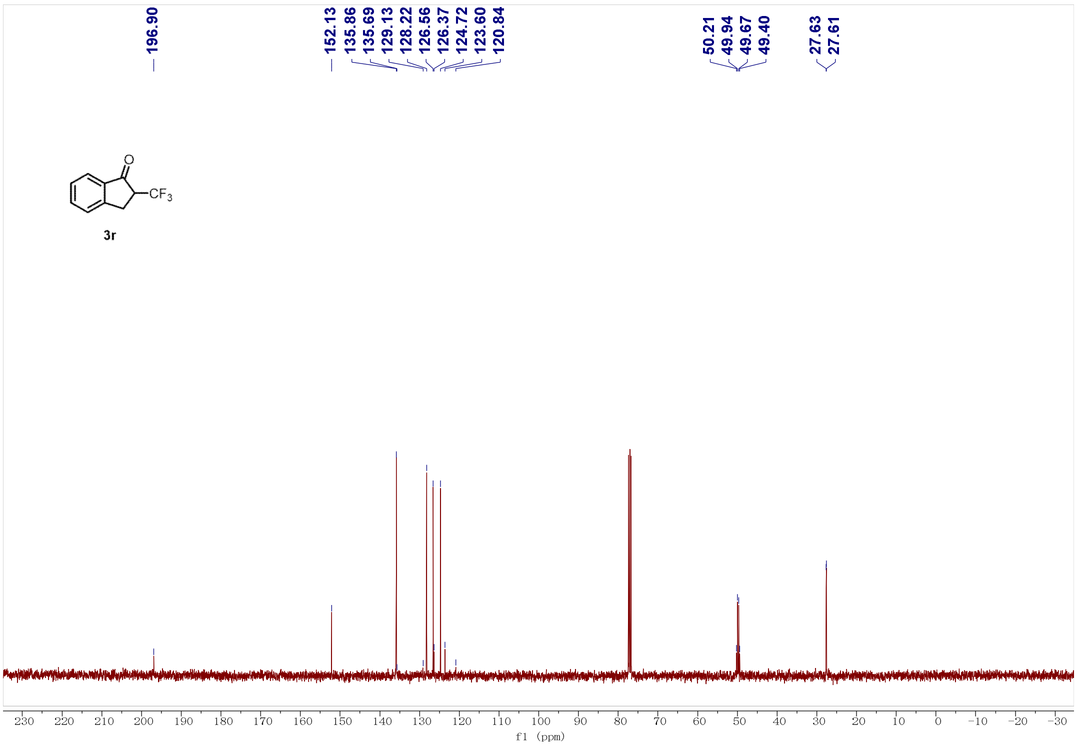

^{1}H NMR spectrum of 3,3,3-trifluoro-1-(thiophen-2-yl)propan-1-one (**3s**) (400 MHz, Chloroform-*d*):

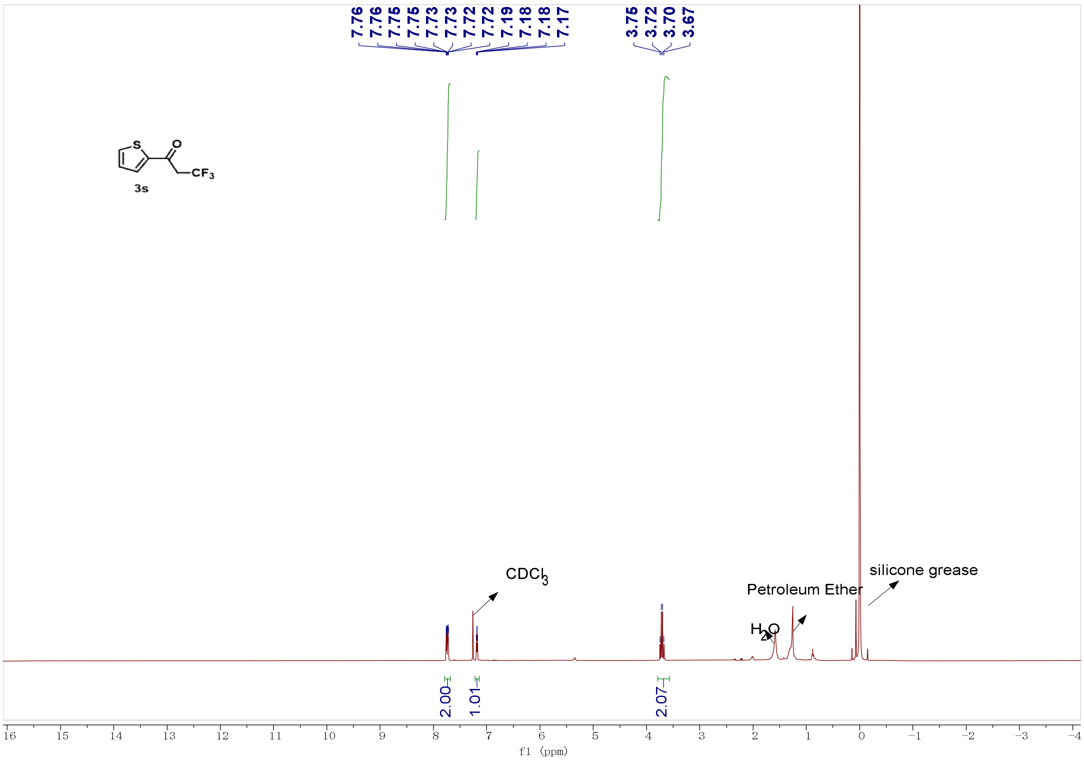

^{19}F NMR spectrum of 3,3,3-trifluoro-1-(thiophen-2-yl)propan-1-one (**3s**) (376 MHz, Chloroform-*d*):

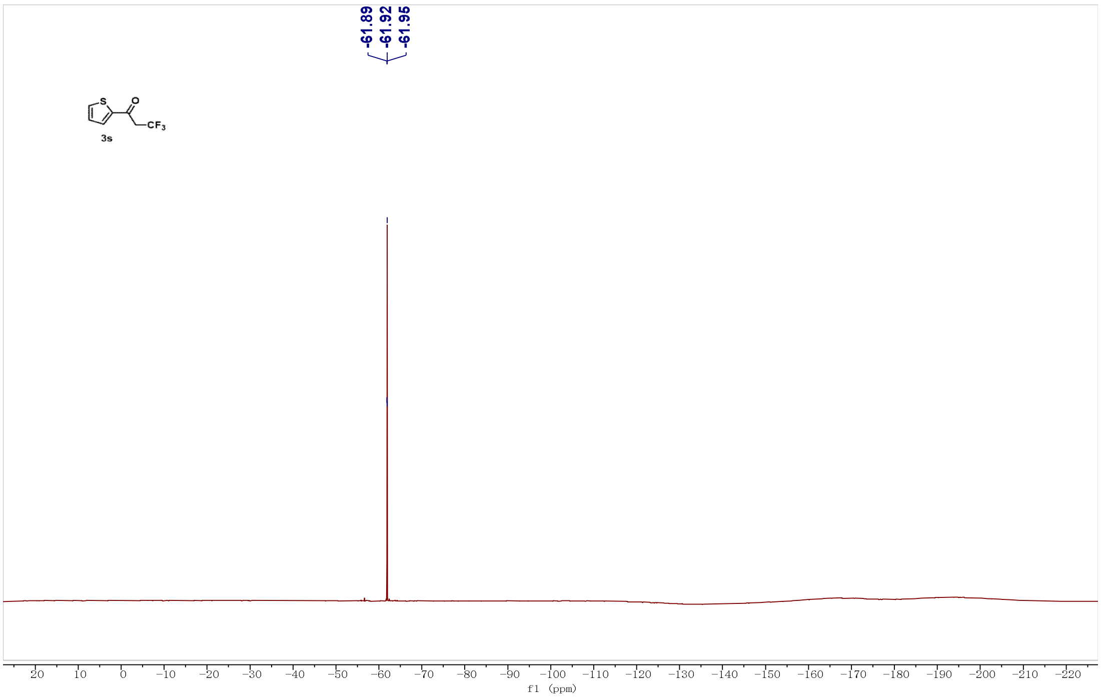

^{19}F NMR spectrum of 1-cyclohexyl-3,3,3-trifluoropropan-1-one (**3t**) (376 MHz, Chloroform-*d*):

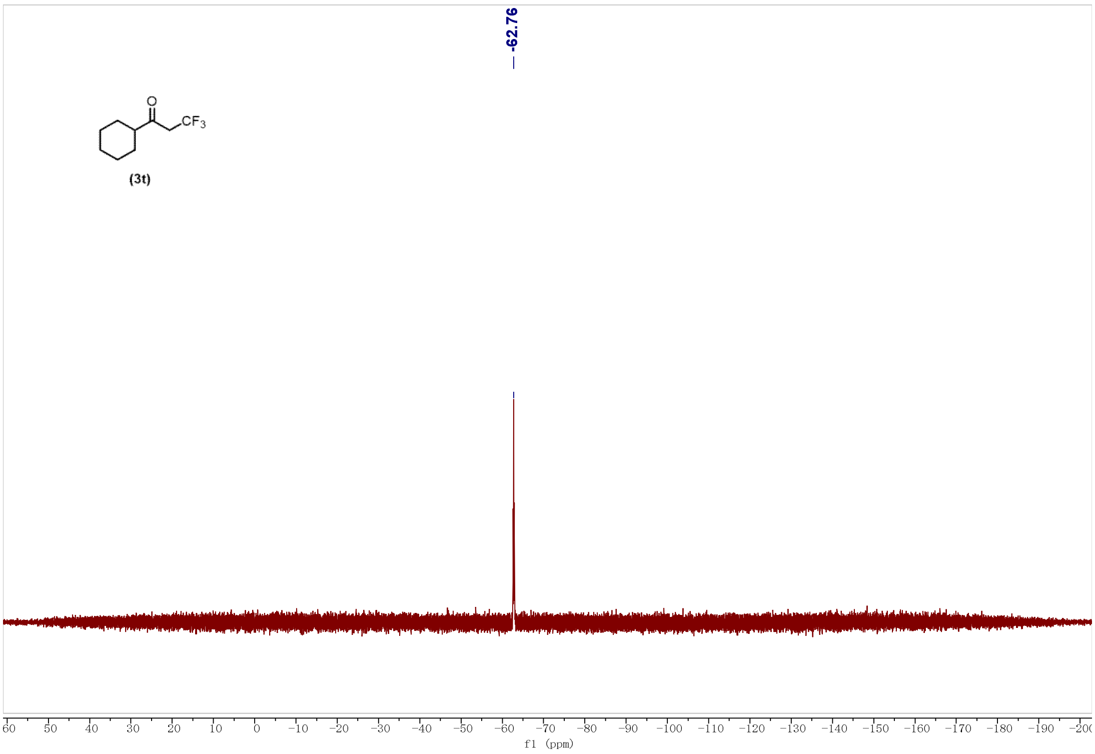

^{19}F NMR spectrum of 1,1,1-trifluoro-5-phenylpentan-3-one (**3v**) (376 MHz, Chloroform-*d*) :

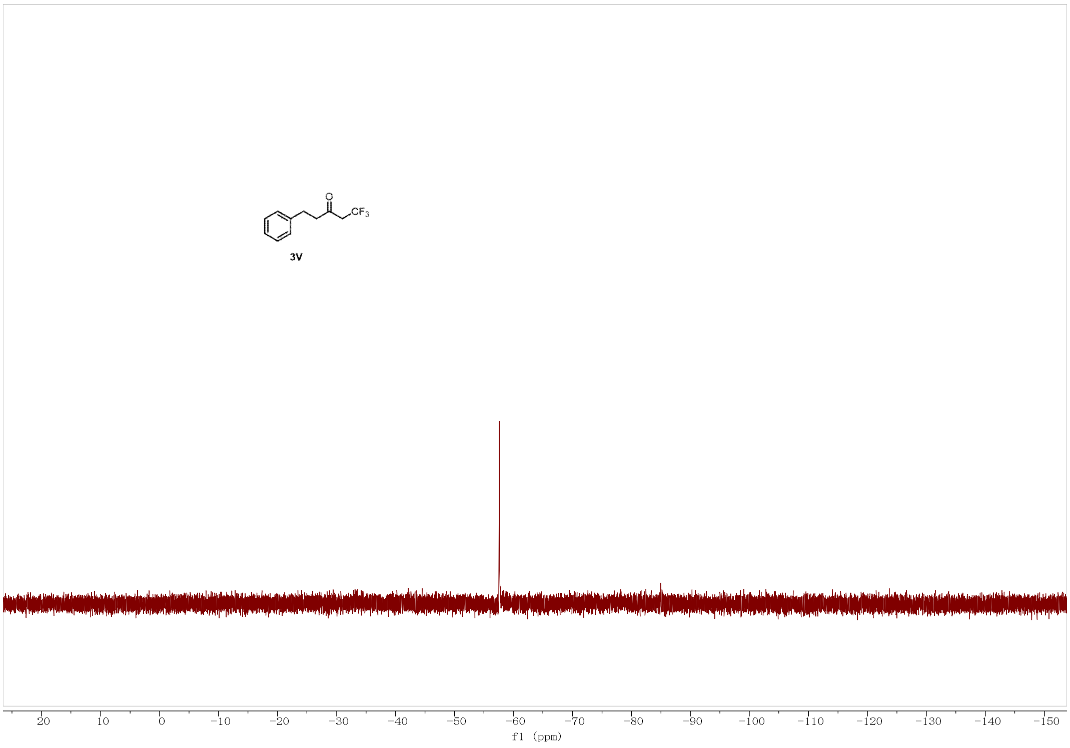

^{1}H NMR spectrum of 1-([1,1'-biphenyl]-4-yl)-3,3,3-trifluoropropan-1-ol **(4)**(400 MHz, Chloroform-*d*):

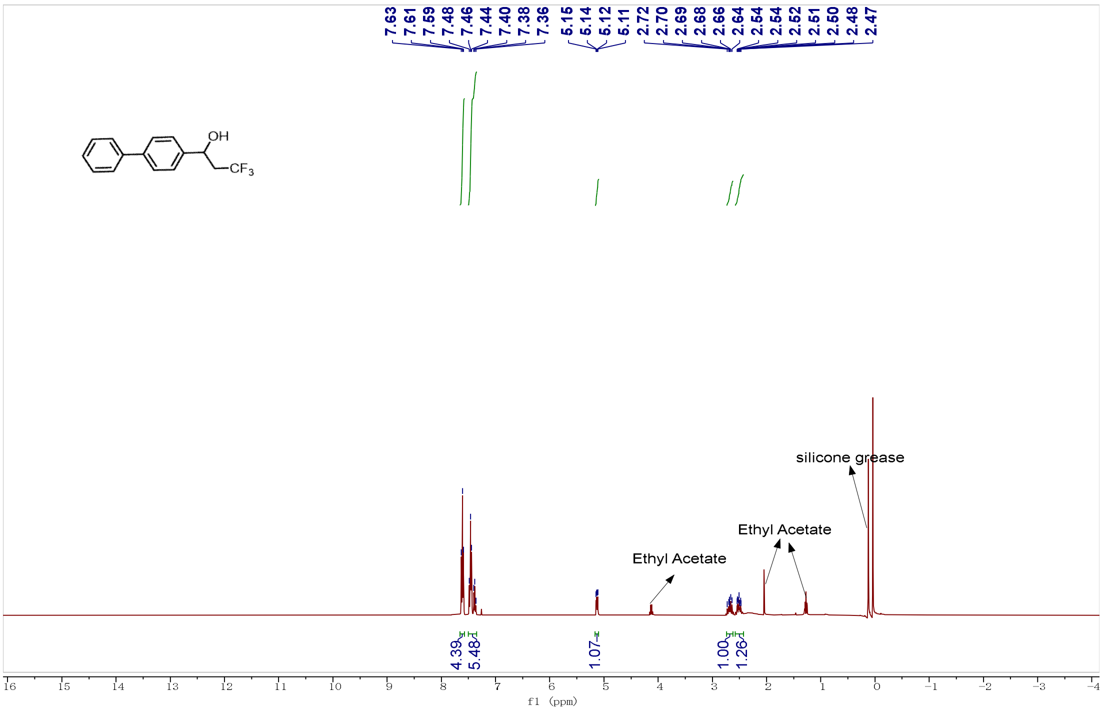

^{19}F NMR spectrum of 1-([1,1'-biphenyl]-4-yl)-3,3,3-trifluoropropan-1-ol **(4)** (376 MHz, Chloroform-*d*):

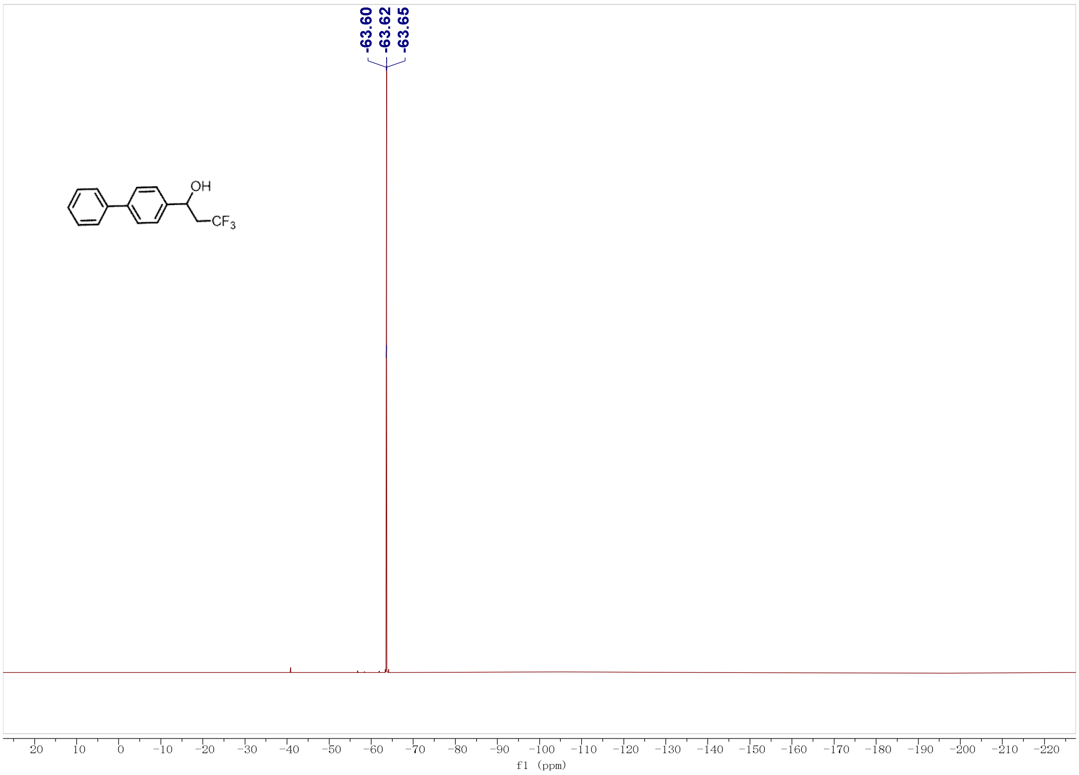

^{13}C NMR spectrum of 1-([1,1'-biphenyl]-4-yl)-3,3,3-trifluoropropan-1-ol **(4)** (101 MHz, Chloroform-*d*):

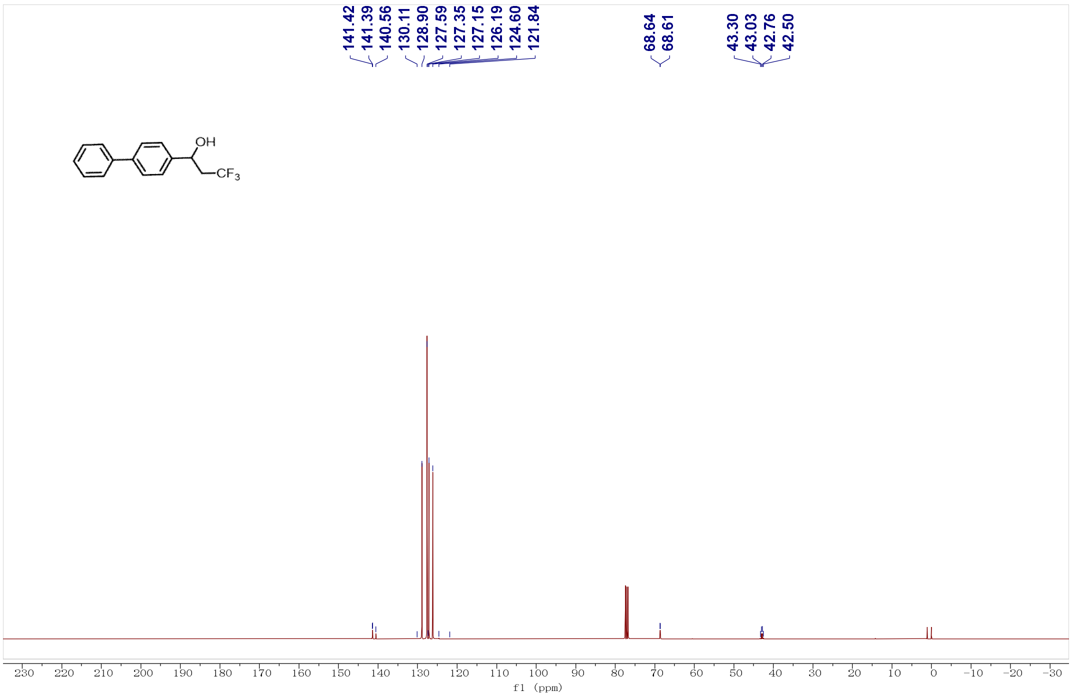

^1H NMR spectrum of 1-([1,1'-biphenyl]-4-yl)-3,3,3-trifluoropropan-1-one oxime **(5)** (400 MHz, Methanol-d_4) ：

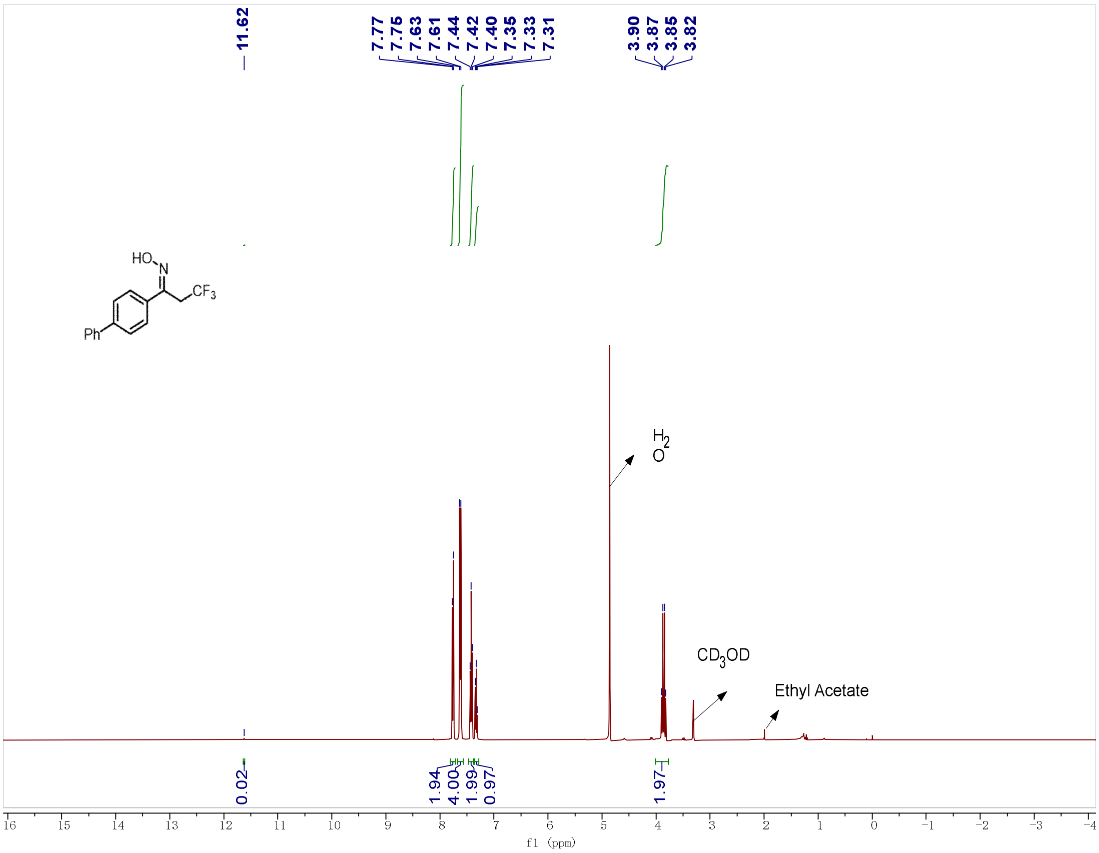

^{19}F NMR spectrum of (Z)-1-([1,1'-biphenyl]-4-yl)-3,3,3-trifluoropropan-1-one oxime **(5)** (376MHz, Methanol-d_4):

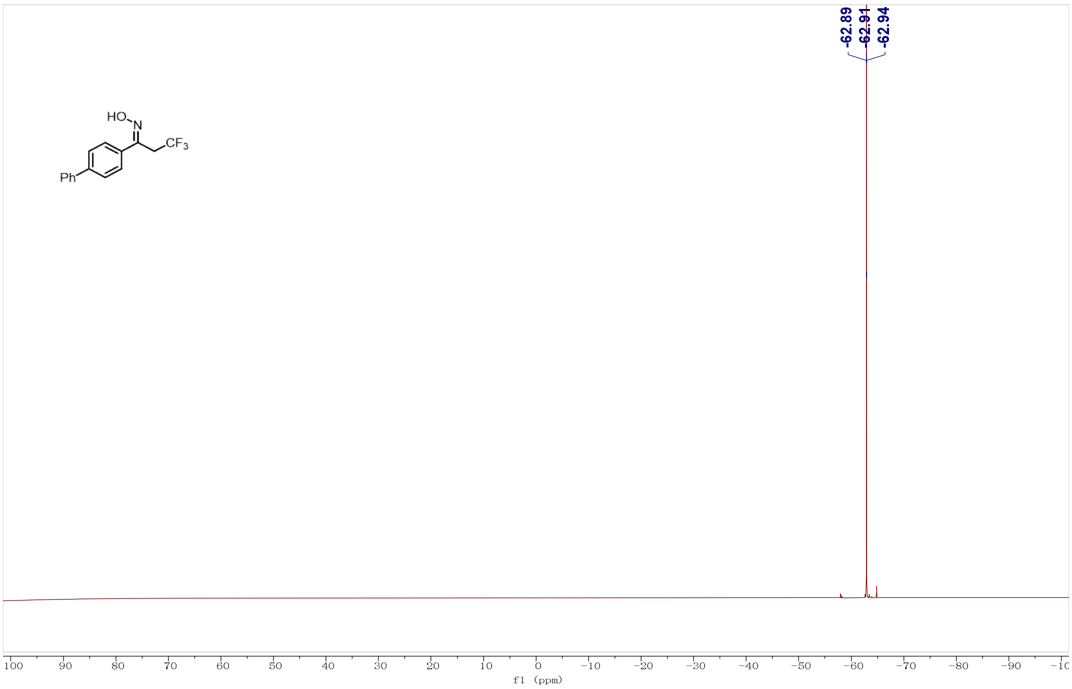

^{13}C NMR spectrum of 1-([1,1'-biphenyl]-4-yl)-3,3,3-trifluoropropan-1-one oxime **(5)** (101 MHz, Methanol-*d*4)：

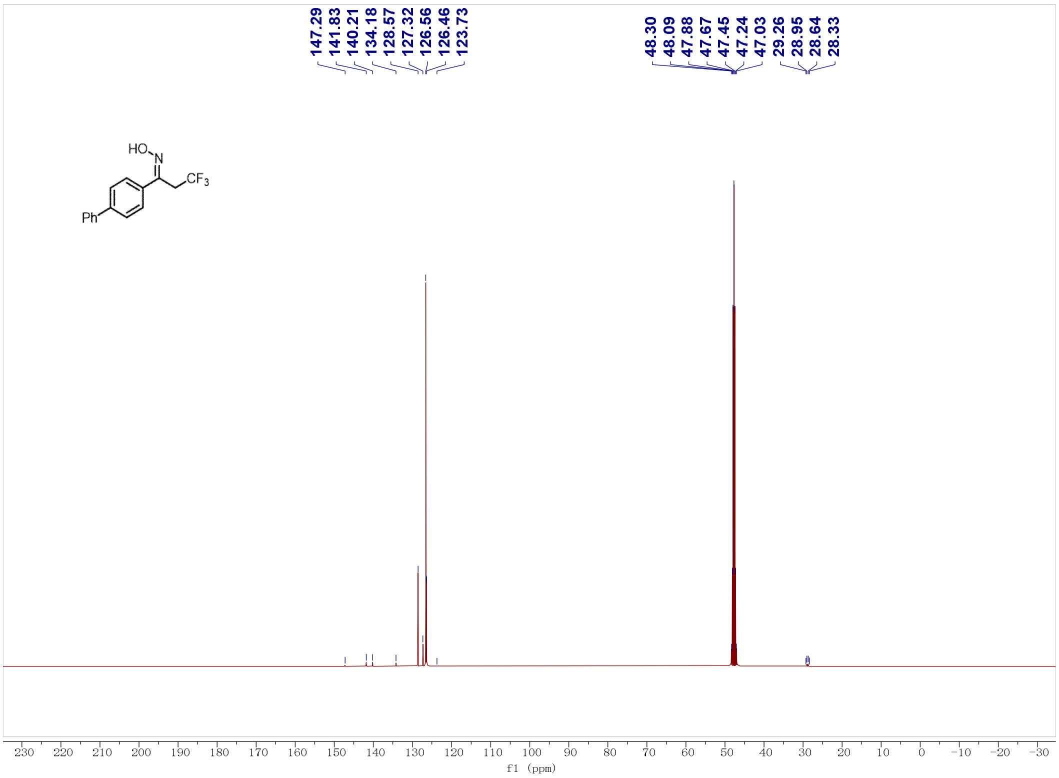

^{1}H NMR spectrum of [1,1'-biphenyl]-4-yl(2-(hydroxymethyl)pyrrolidin-1-yl)methanone **(6)** (400 MHz, Chloroform-*d*)：

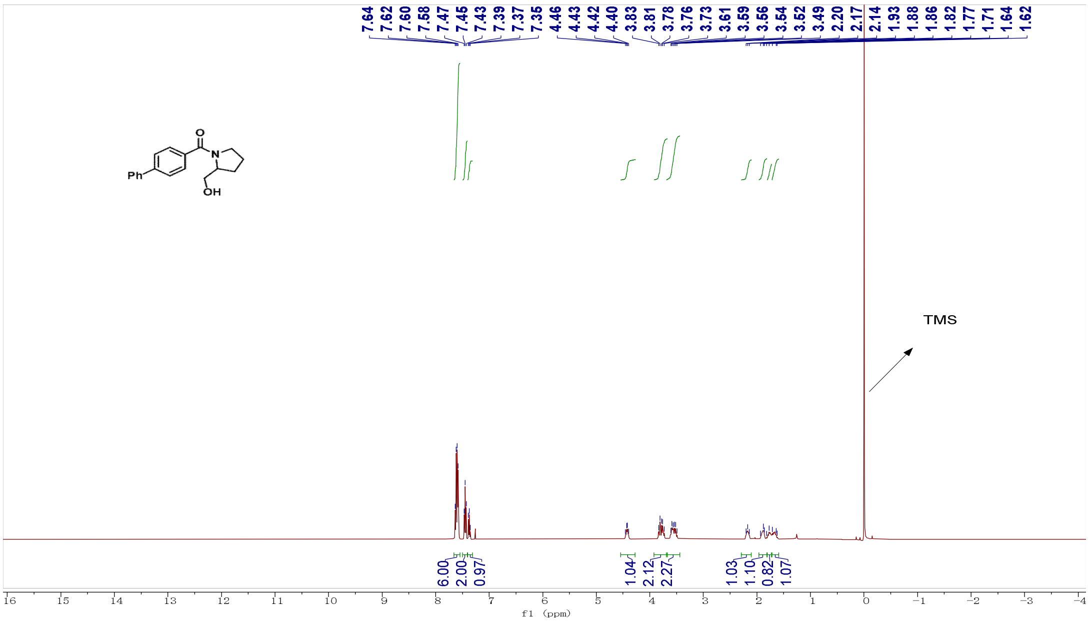

^{13}C NMR spectrum of [1,1'-biphenyl]-4-yl(2-(hydroxymethyl)pyrrolidin-1-yl)methanone **(6)** (101 MHz, Chloroform-*d*):

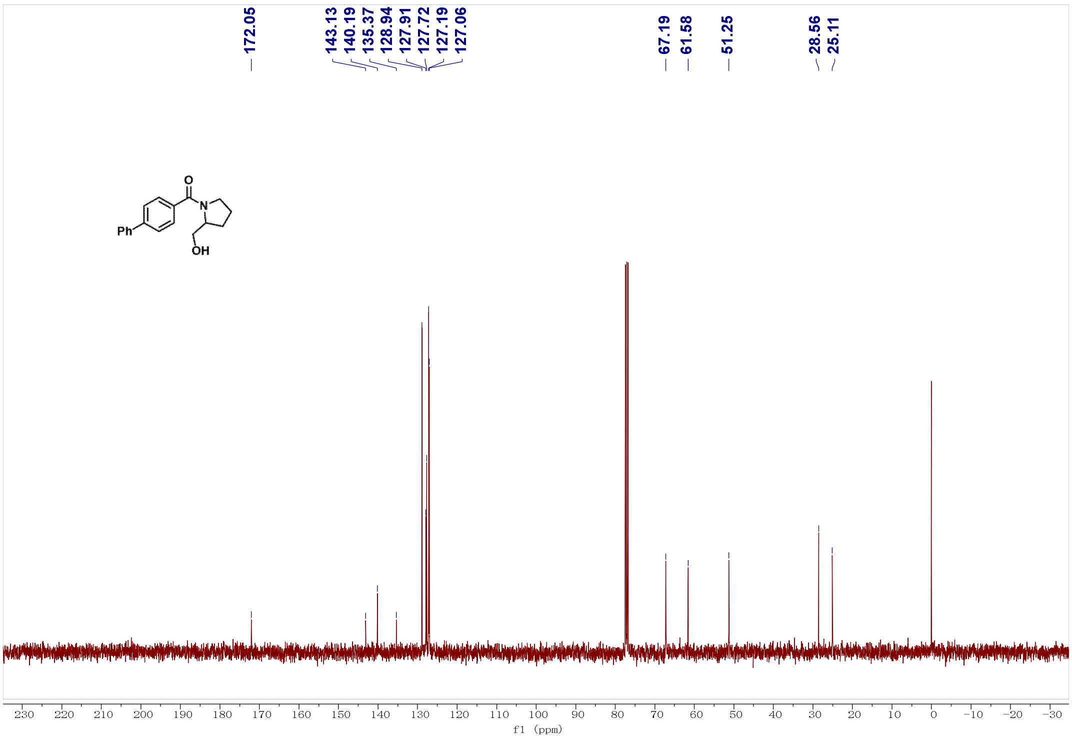

Supplement: Supplementary file 1 [file molecules-29-05622-s001.zip › molecules-3269871-supplementary.pdf]
